# iCRBP-LKHA: Large convolutional kernel and hybrid channel-spatial attention for identifying circRNA-RBP interaction sites

**DOI:** 10.1371/journal.pcbi.1012399

**Published:** 2024-08-22

**Authors:** Lin Yuan, Ling Zhao, Jinling Lai, Yufeng Jiang, Qinhu Zhang, Zhen Shen, Chun-Hou Zheng, De-Shuang Huang

**Affiliations:** 1 Key Laboratory of Computing Power Network and Information Security, Ministry of Education, Shandong Computer Science Center, Qilu University of Technology (Shandong Academy of Sciences), Jinan, China; 2 Shandong Engineering Research Center of Big Data Applied Technology, Faculty of Computer Science and Technology, Qilu University of Technology (Shandong Academy of Sciences), Jinan, China; 3 Shandong Provincial Key Laboratory of Computer Networks, Shandong Fundamental Research Center for Computer Science, Jinan, China; 4 Eastern Institute for Advanced Study, Eastern Institute of Technology, Ningbo, China; 5 School of Computer and Software, Nanyang Institute of Technology, Nanyang, China; 6 Key Lab of Intelligent Computing and Signal Processing of Ministry of Education, School of Artificial Intelligence, Anhui University, Hefei, China; Shandong University, CHINA

## Abstract

Circular RNAs (circRNAs) play vital roles in transcription and translation. Identification of circRNA-RBP (RNA-binding protein) interaction sites has become a fundamental step in molecular and cell biology. Deep learning (DL)-based methods have been proposed to predict circRNA-RBP interaction sites and achieved impressive identification performance. However, those methods cannot effectively capture long-distance dependencies, and cannot effectively utilize the interaction information of multiple features. To overcome those limitations, we propose a DL-based model iCRBP-LKHA using deep hybrid networks for identifying circRNA-RBP interaction sites. iCRBP-LKHA adopts five encoding schemes. Meanwhile, the neural network architecture, which consists of large kernel convolutional neural network (LKCNN), convolutional block attention module with one-dimensional convolution (CBAM-1D) and bidirectional gating recurrent unit (BiGRU), can explore local information, global context information and multiple features interaction information automatically. To verify the effectiveness of iCRBP-LKHA, we compared its performance with shallow learning algorithms on 37 circRNAs datasets and 37 circRNAs stringent datasets. And we compared its performance with state-of-the-art DL-based methods on 37 circRNAs datasets, 37 circRNAs stringent datasets and 31 linear RNAs datasets. The experimental results not only show that iCRBP-LKHA outperforms other competing methods, but also demonstrate the potential of this model in identifying other RNA-RBP interaction sites.

## Author summary

The interaction between circRNAs and RBPs is one of the main activities of circRNAs. CircRNAs participate in the occurrence and development of diseases by interacting with RBPs. Identifying circRNA-RBP interaction sites have become a fundamental step for exploring the role of circRNA in the occurrence and progression of diseases. Many computational methods have been proposed to predict circRNA-RBP interaction sites. Nevertheless, they still have several limitations. For long nucleotide sequence data of circRNA, traditional CNN or LSTM cannot effectively capture long-distance dependencies (relationships between non-adjacent nucleotides in a circRNA). Furthermore, existing methods fail to effectively utilize the interaction information of multiple features, and insufficient consideration of interaction information leads to biased circRNA-RBP interaction relationships. To overcome these limitations, we propose iCRBP-LKHA, based on a large convolutional kernel and hybrid channel-spatial attention for identifying circRNA-RBP interaction sites. We compared its performance with state-of-the-art DL-based methods on 37 circRNAs datasets, 37 circRNAs stringent datasets and 31 linear RNAs datasets. Experimental results not only show that iCRBP-LKHA outperforms competing methods, but also demonstrate the potential of this model in identifying other RNA-RBP interaction sites.

## Introduction

Circular RNAs (circRNAs) are a large class of non-coding RNAs that ubiquitously exist in many species [1,2], which have the characteristics of stable structure and high tissue-specific expression [3,4]. CircRNAs affect transcription and translation processes by acting as transcriptional regulators, microRNA (miR) sponges and interacting with RNA binding proteins (RBPs) [5]. The interaction with RBPs is one of the main activities of circRNAs. CircRNAs participate in the occurrence and development of diseases by interacting with RBPs. For example, circCwc27 plays a critical role in Alzheimer’s disease pathogenesis by binding the purine-rich element-binding protein A (Pur-α) [6]. The interaction of circFndc3b and RBP FUS improves the function reconstruction of myocardium after infarction [7]. Identifying circRNA-RBP interaction sites have become a fundamental step for exploring the role of circRNA in the occurrence and progression of diseases [8–12].

Since high-throughput sequencing technology is expensive and time-consuming, researchers have proposed many computational methods to predict circRNA-RBP interaction sites [13–15]. Recently, many DL-based methods have achieved remarkable results on predicting circRNA-RBP interaction sites. For example, CRIP [16] used a stacked codon-based encoding scheme and a hybrid deep learning architecture incorporating CNN and LSTM to predict circRNA-RBP interaction sites. CircSLNN [17] predicted circRNA-RBP interaction sites by combining CNN, LSTM and conditional random field (CRF). PASSION [18] selected optimal feature subset from six feature encoding schemes using XGBoost algorithm, then applied CNN and BiLSTM to identify the interactions between circRNAs and RBPs. iCircRBP-DHN [19] proposed a novel encoding schemes CircRNA2Vec and used deep multi-scale residual network (MSRN) and self-attention BiGRUs to predict circRNA-RBP interaction sites. Inspired by iCircRBP-DHN, CRBPDL identified circRNA-RBP interaction sites by introducing five feature encoding schemes and AdaBoost algorithm [20]. ASCRB used five feature encoding schemes and channel attention mechanisms to identify circRNA-RBP interaction sites [21]. These methods have achieved impressive results in predicting circRNA-RBP interaction sites. Nevertheless, they still have several limitations. For long nucleotide sequence data of circRNA, traditional CNN or LSTM cannot effectively capture long-distance dependencies (relationships between non-adjacent nucleotides in a circRNA). Furthermore, existing methods fail to effectively utilize the interaction information of multiple features, and insufficient consideration of interaction information leads to biased circRNA-RBP interaction relationships.

**Fig 1:**
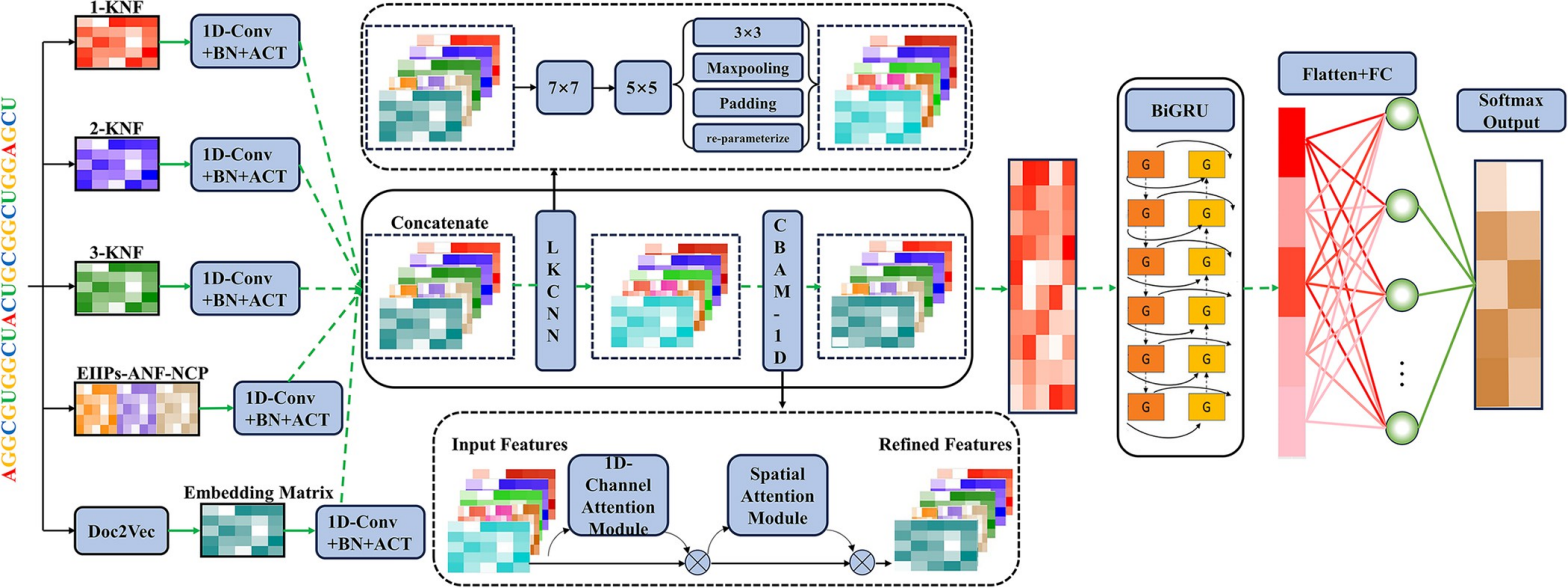
Schematic overview of iCRBP-LKHA. The input circRNA sequence is encoded by five schemes: KNF, Doc2Vec, EIIP, CCN and ANF. Then, the concatenated features are fed to a deep neural network architecture formed by LKCNN, CBAM-1D and BiGRU to extract local information, global context information and multiple features interaction information. Finally, a flattened layer integrates the resulting information followed by a fully connected layer with softmax for label classification.

To overcome these limitations, we propose iCRBP-LKHA, based on a large convolutional kernel and hybrid channel-spatial attention for identifying circRNA-RBP interaction sites. iCRBP-LKHA adopts five sequence encoding schemes, including k-nucleotide frequency (KNF), Doc2Vec, electron-ion interaction pseudopotential (EIIP) [22], chemical characteristic of nucleotide (CCN) and accumulated nucleotide frequency (ANF) to extract comprehensive feature information. Subsequently, the large kernel convolutional neural network (LKCNN) is applied to capture long-distance dependencies and update the feature maps [23]. Then, the updated feature maps are fed to a modified hybrid channel-spatial attention module CBAM-1D (convolutional block attention module with one-dimensional (1D) convolution) [24], which focuses on important features, multiple features interaction information and suppresses unnecessary features. Finally, the refined feature is fed to a bidirectional gated recurrent unit (BiGRU) network to identify circRNA-RBP interaction sites [25,26]. The schematic overview of iCRBP-LKHA is shown in Fig 1. To verify the effectiveness and generalizability of iCRBP-LKHA, we compared the performance of iCRBP-LKHA with state-of-the-art methods on 37 circRNAs and 31 liearRNAs datasets, respectively. Experimental results show that iCRBP-LKHA outperforms other competing methods. Moreover, we observe that iCRBP-LKHA can accurately identify linear RNA-RBP binding sites.

## Results

### Model performance under different network layers

The performance of a neural network depends heavily on its architecture, especially the network depth. Compared with shallow neural networks, deep neural networks exhibited stronger ability to extract features and learn complex representations. However, too many layers can lead to overfitting and reduce model performance. In this section, we analyze the impact of network depth by reducing or increasing the convolutional blocks in LKCNN. iCRBP-LKHA adds two 1x1 convolutional layers or reduces two 1x1 convolutional layers, and the modified models are called iCRBP-LKHA+2 and iCRBP-LKHA-2 respectively.

**Fig 2:**
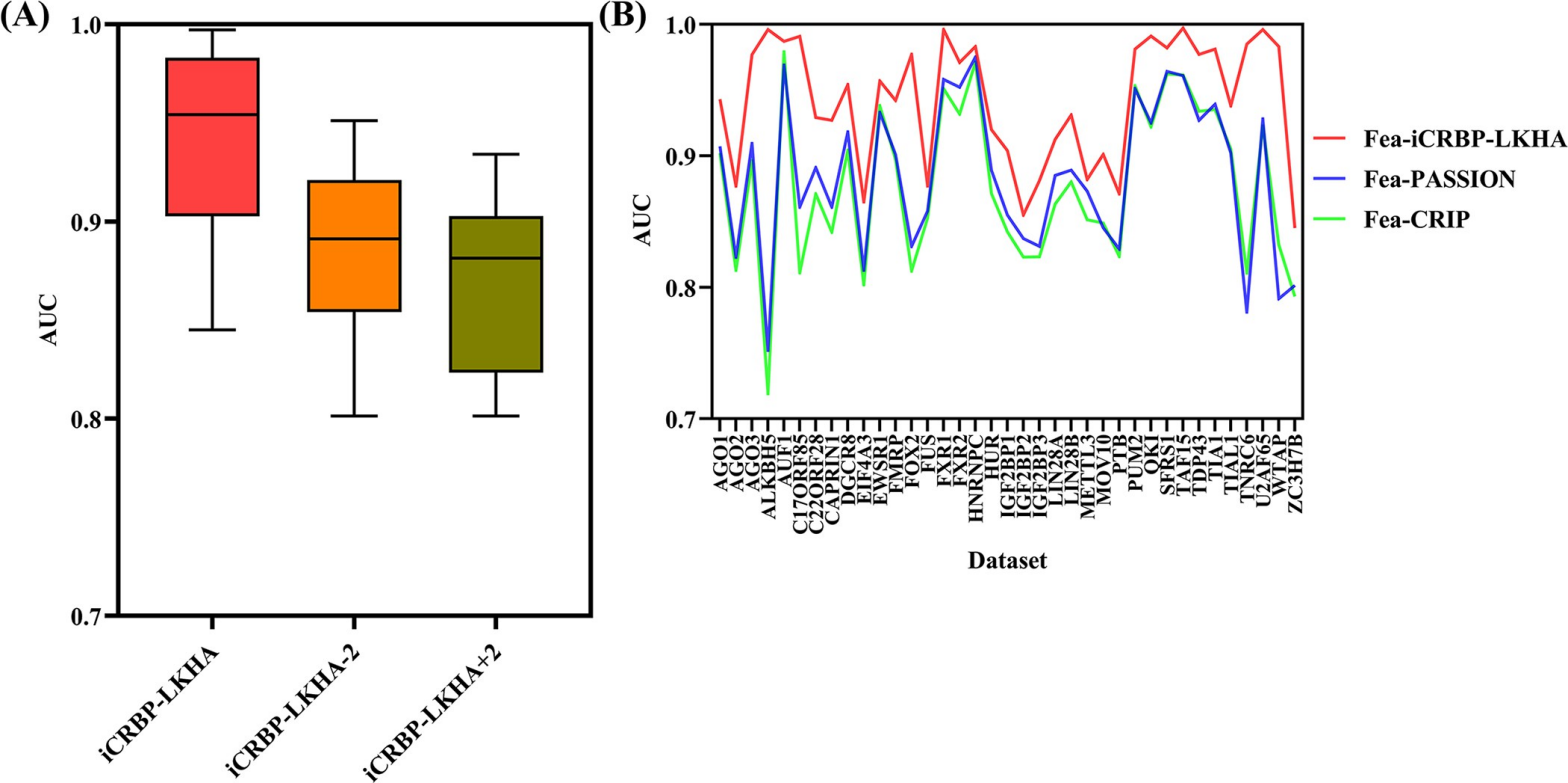
(A) Performance comparison of different network depths in terms of distribution of AUCs across 37 circRNAs datasets experiments. (B) Performance comparison of deep neural network architectures among multiple feature encoding schemes as visualized in line graph.

We compared the prediction performance of iCRBP-LKHA, iCRBP-LKHA+2 and iCRBP-LKHA-2 on 37 circRNAs datasets. As shown in Fig 2A, iCRBP-LKHA performs better than iCRBP-LKHA+2 and iCRBP-LKHA-2. As shown in S1 Table, the average AUC value of iCRBP-LKHA is 0.9423, which is higher than that of iCRBP-LKHA-2 (0.8878) and iCRBP-LKHA+2 (0.8694). iCRBP-LKHA outperforms other competing methods in 29 of the 37 datasets.

### Model performance under different feature encoding schemes

The performance of neural networks is affected by feature encoding scheme. To evaluate the contribution of the feature encoding scheme we used (named Fea-iCRBP-LKHA), under the same neural network architecture of iCRBP-LKHA, we replaced the original feature encoding scheme with the encoding scheme of PASSION (named Fea-PASSION) [18] and the encoding scheme of CRIP (named Fea-CRIP) [16], which are two widely used encoding schemes. The AUC line graphs of the three methods on 37 circRNAs datasets were shown in Fig 2B. The AUC values of the three methods on 37 circRNAs datasets were listed in S2 Table.

As shown in Fig 2B, Fea-iCRBP-LKHA performs better than Fea-PASSION and Fea-CRIP on all datasets. As shown in S2 Table, the average AUC of Fea-iCRBP-LKHA is 0.9423, which is higher than Fea-PASSION’s 0.8844 and Fea-CRIP’s 0.8772. The experimental results clearly demonstrate the effectiveness of the adopted feature encoding scheme.

### Contributions of different encoding schemes

To evaluate the contribution of each encoding scheme relative to all five encoding schemes together, we conducted leave-one-encoding-out experiments on 37 circRNAs datasets. We trained the iCRBP-LKHA models using merely four encoding schemes with the same hyper-parameters and compared the performances with the models trained with all encoding schemes together.

**Fig 3:**
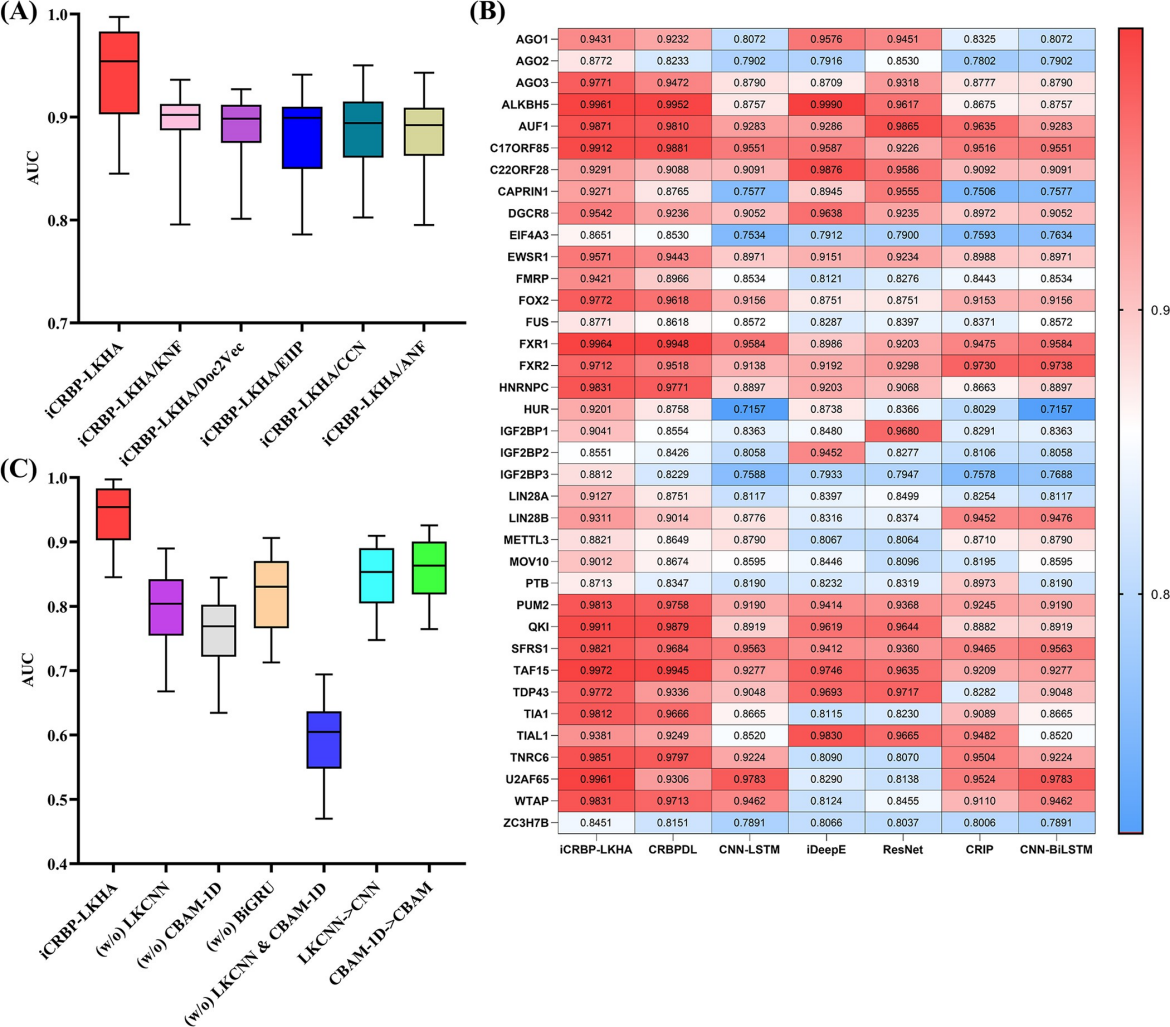
(A) Performance comparison of different encoding schemes combinations in terms of distribution of AUCs across 37 circRNA datasets experiments. iCRBP-LKHA means using all five encoding schemes. iCRBP-LKHA/KNF means using other four encoding schemes except KNF. (B) Determination of the suitable neural network architecture from multiple possible neural architectures and algorithms in terms of distribution of AUCs as shown in the heatmap. (C) Performance comparison of different neural network architectures in terms of distribution of AUCs in ablation experiments. iCRBP-LKHA means our proposed neural network architecture. (w/o)LKCNN means neural network architecture without LKCNN. LKCNN->CNN means LKCNN is replaced by CNN.

As shown in Fig 3A, the models suffer from performance drop when using different four encoding schemes. Among these five encoding schemes, ANF is the most important encoding scheme and CCN is the second most important encoding scheme. The results demonstrate the effectiveness of five encoding schemes used together. The detailed results were recorded in S3 Table.

### Performance of neural network architecture in iCRBP-LKHA

To evaluate the performance of the neural network architecture in iCRBP-LKHA, the same five features (see Section *Feature encoding* in Materials and methods) were fed to six CNN-based methods and compared the performance of these methods with iCRBP-LKHA. These methods are iDeepE [27], ResNet [28], CRIP [16], CRBPDL [20], CNN-BiLSTM and CNN-LSTM. iDeepE consists of two multi-channel CNN layers. ResNet is composed of multiple multi-channel CNN layers and residual building blocks (RBB). CRIP uses a CNN layer to learn high-level features, and then uses RNN layer to learn long dependency in the sequence. CRBPDL consists of deep MSRN and BiGRUs.

As shown in Fig 3B, our model iCRBP-LKHA achieved the highest AUC values in 26 of the 37 datasets. iCRBP-LKHA is slightly worse than iDeepE on dataset ALKBH5 and DGCR8, and slightly worse than CNN-BiLSTM on dataset FXR2. The average values of iCRBP-LKHA, CRBPDL, CNN-LSTM, iDeepE, ResNet, CRIP and CNN-BiLSTM are 0.9424, 0.9188, 0.8728, 0.8854, 0.8877, 0.8760 and 0.8733, respectively. The average AUC of iCRBP-LKHA is higher than competing methods. The results were recorded in S4 Table. These results demonstrate the effectiveness of the neural network architecture of iCRBP-LKHA.

### Contribution of LKCNN, CBAM-1D and BiGRU

In this section, we analyze the contributions of LKCNN, CBAM-1D and BiGRU. We constructed five different models, specifically, (i) iCRBP-LKHA without LKCNN; (ii) iCRBP-LKHA without CBAM-1D; (iii) iCRBP-LKHA without BiGRU; (IV) iCRBP-LKHA without LKCNN and CBAM-1D; (V) LKCNN replaced by CNN in iCRBP-LKHA; (VI) CBAM-1D replaced by CBAM in iCRBP-LKHA. We trained these models using the same hyper-parameters and compared the performances with iCRBP-LKHA. The AUC values of these six models on 37 datasets were listed in S5 Table.

As shown in Fig 3C, when we remove LKCNN, CBAM-1D or BiGRU, the model performance drops by 15.4%, 19.6% and 12.7% respectively, and after removing LKCNN and CBAM-1D, iCRBP-LKHA suffers performance degradation. After replacing LKCNN and CBAM-1D with CNN and CBAM respectively, the results of both models were worse than those of iCRBP-LKHA. Experimental results prove that LKCNN, CBAM-1D and BiGRU are all beneficial to circRNA-RBP interaction prediction, and iCRBP-LKHA outperforms traditional CNN and CBAM.

### Comparison with traditional machine learning methods

**Fig 4:**
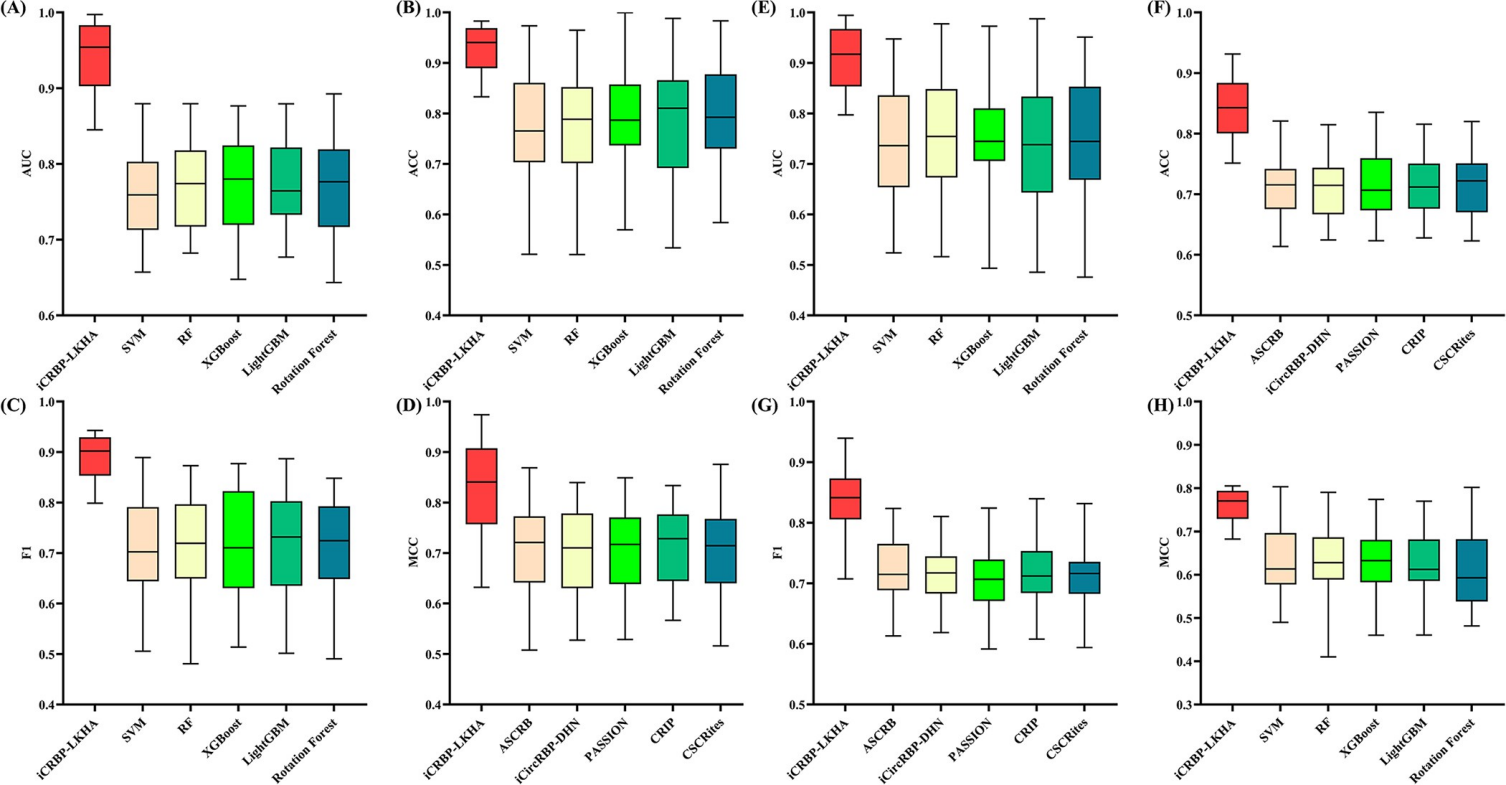
(A)-(D) Performance comparison of iCRBP-LKHA with shallow learning algorithms in terms of distribution of AUCs, ACCs, F1s and MCCs across 37 circRNAs datasets. (E)-(H) Performance comparison of iCRBP-LKHA with shallow learning algorithms in terms of distribution of AUCs, ACCs, F1s and MCCs across 37 circRNAs stringent datasets.

In this section, we compared iCRBP-LKHA with SVM (Support Vector Machine) [29], Random Forest (RF) [30], XGBoost [31], LightGBM [32] and Rotation Forest [33] to test the prediction performance of iCRBP-LKHA. We used the same feature sets and applied feature selection method PCA (Principal Component Analysis) [34] followed by application of these shallow learning algorithms. Here we implement these methods, which are trained and evaluated using the 37 circRNAs datasets and 37 circNRAs stringent datasets. The detailed parameters of these shallow learning algorithms were presented in Table 1. All experiments were done on an NVIDIA RTX 3090 GPU with 24 GB VRAM, and the evaluation metrics are AUC, ACC, F1 and MCC. The results were shown in Fig 4. The AUC of the 37 circRNAs datasets were listed in Table 2, and ACC, F1and MCC of the 37 circRNAs datasets were listed in S6, S7 and S8 Tables respectively. The AUC, ACC, F1 and MCC of the 37 circRNAs stringent datasets were listed in S9, S10, S11 and S12 Tables respectively.

**Table 1:** The detailed parameters of shallow learning algorithms.

Method	Parameter
SVM	Probability = True, kernel = ‘poly’
RF	Max_feature = 0.2, n_estimators = 100
XGBoost	penalty parameter = 0.1, max_depth = 8
LightGBM	learning_rate = 0.05, n_estimators = 100
Rotation Forest	set the number of feature subsets as 4, the number of decision trees as 3

**Table 2:** Comparison of AUC between iCRBP-LKHA and five shallow learning algorithms on 37 circRNA datasets. Bold data represent the best AUC values of experimental results.

Dataset37	iCRBP-LKHA	SVM	RF	XGBoost	LightGBM	Rotation Forest
AGO1	**0.9431±0.001**	0.7907	0.7681	0.7783	0.7469	0.7513
AGO2	**0.8772±0.001**	0.6768	0.6928	0.7168	0.6781	0.6623
AGO3	**0.9771±0.003**	0.7463	0.7803	0.7366	0.7393	0.8066
ALKBH5	**0.9961±0.001**	0.8032	0.8795	0.858	0.8572	0.8255
AUF1	**0.9871±0.001**	0.7963	0.8234	0.8767	0.8274	0.8302
C17ORF85	**0.9912±0.001**	0.8215	0.8449	0.7971	0.8657	0.8126
C22ORF28	**0.9291±0.003**	0.7491	0.7498	0.7817	0.7899	0.813
CAPRIN1	**0.9271±0.001**	0.7261	0.6958	0.7219	0.7386	0.7099
DGCR8	**0.9542±0.002**	0.7928	0.7767	0.7956	0.7496	0.8011
EIF4A3	**0.8651±0.005**	0.6882	0.7091	0.6476	0.6831	0.6434
EWSR1	**0.9571±0.003**	0.8151	0.8013	0.7875	0.8014	0.8019
FMRP	**0.9421±0.001**	0.7206	0.7433	0.7227	0.7522	0.7323
FOX2	**0.9772±0.003**	0.8028	0.7848	0.8223	0.7855	0.8545
FUS	**0.8771±0.001**	0.7691	0.7393	0.7229	0.727	0.7588
FXR1	**0.9964±0.003**	0.8795	0.8169	0.8712	0.8793	0.8926
FXR2	**0.9712±0.002**	0.7843	0.8336	0.8205	0.783	0.7932
HNRNPC	**0.9831±0.003**	0.7858	0.809	0.7833	0.8759	0.8351
HUR	**0.9201±0.013**	0.6965	0.7585	0.7723	0.747	0.7392
IGF2BP1	**0.9041±0.003**	0.705	0.7024	0.7171	0.7435	0.6797
IGF2BP2	**0.8551±0.003**	0.6698	0.7182	0.6713	0.6929	0.68
IGF2BP3	**0.8812±0.001**	0.6655	0.6971	0.6552	0.677	0.7007
LIN28A	**0.9127±0.004**	0.7325	0.7161	0.6939	0.6997	0.7236
LIN28B	**0.9311±0.012**	0.7484	0.7757	0.7626	0.7878	0.7498
METTL3	**0.8821±0.011**	0.7557	0.6822	0.7381	0.7473	0.7093
MOV10	**0.9012±0.022**	0.7032	0.6953	0.6923	0.717	0.733
PTB	**0.8713±0.015**	0.6722	0.735	0.7136	0.7209	0.6874
PUM2	**0.9813±0.012**	0.7786	0.8498	0.7782	0.8221	0.83
QKI	**0.9911±0.006**	0.8553	0.8606	0.7801	0.8231	0.8325
SFRS1	**0.9821±0.006**	0.8181	0.7741	0.8634	0.7793	0.8647
TAF15	**0.9972±0.002**	0.8339	0.831	0.8503	0.8353	0.8828
TDP43	**0.9772±0.003**	0.7506	0.7685	0.8265	0.7646	0.805
TIA1	**0.9812±0.009**	0.8383	0.8187	0.8549	0.779	0.8065
TIAL1	**0.9381±0.001**	0.7405	0.7441	0.8035	0.7623	0.7687
TNRC6	**0.9851±0.002**	0.801	0.8253	0.7959	0.8361	0.7766
U2AF65	**0.9961±0.002**	0.7593	0.7756	0.8319	0.8208	0.763
WTAP	**0.9831±0.004**	0.8184	0.8141	0.8341	0.8215	0.7861
ZC3H7B	**0.8451±0.008**	0.6571	0.7052	0.6867	0.6841	0.6685
**AVG**	**0.9424±0.003**	0.7608	0.7702	0.7720	0.7714	0.7706

The advantages of iCRBP-LKHA over the shallow learning algorithms are obvious. In 37 circRNAs datasets, in terms of AUC, iCRBP-LKHA achieves the best performance on all datasets. The average AUCs of iCRBP-LKHA, SVM, RF, XGBoost, LightGBM and Rotation Forest are 0.9424, 0.7596, 0.7571, 0.7541, 0.7429 and 0.7481 respectively. In 37 circNRAs stringent datasets, iCRBP-LKHA achieved the highest AUC values on all datasets. The average AUCs of iCRBP-LKHA, SVM, RF, XGBoost, LightGBM and Rotation Forest are 0.9078, 0.7496, 0.7571, 0.7541, 0.7429 and 0.7481 respectively. The same was found in ACC, F1 and MCC. The results demonstrate the advantages of iCRBP-LKHA over shallow learning algorithms.

### The prediction performance on 37 circRNAs datasets

**Fig 5:**
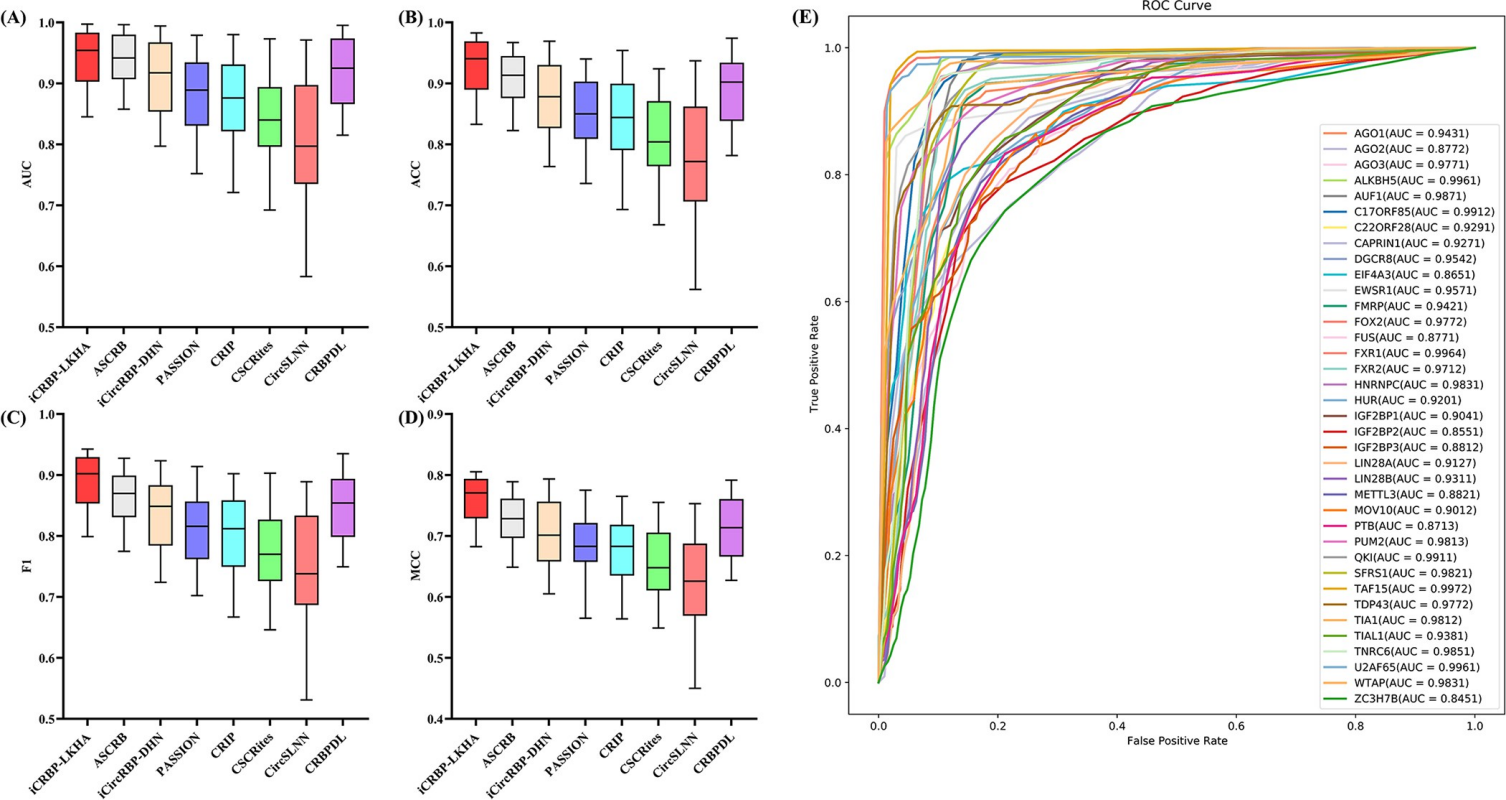
(A) Performance comparison of iCRBP-LKHA with state-of-the-art DL-based methods in terms of distribution of AUCs across 37 circRNAs datasets. (B) ACCs across 37 circRNAs datasets. (C) F1s across 37 circRNAs datasets. (D) MCCs across 37 circRNAs datasets. (E) The ROC curves of iCRBP-LKHA on 37 circRNAs datasets.

In this section, we compared iCRBP-LKHA with ASCRB [21], CRBPDL [20], iCircRBP-DHN [19], PASSION [18], CRIP [16], CSCRites [35] and CircSLNN [17] to test the prediction performance of iCRBP-LKHA. CSCRites uses multiple convolutional layers to identify cancer-specific circRNA-RBP binding sites. The eight methods were tested on the 37 benchmark datasets of circRNAs. The AUCs of ASCRB, CRBPDL, iCircRBP-DHN, PASSION, CRIP, CSCRites and CircSLNN are obtained from their references [16–21,35]. The AUCs of the competing methods are either retained to three decimal places or retained to four decimal places. The experimental results of iCRBP-LKHA are retained to four decimal places. Retaining three or four decimal places does not affect the comparison results. The results were shown in Fig 5A–5D. The ROC curves of iCRBP-LKHA were shown in Fig 5E. The AUC were listed in Table 3 and the last row is the average AUC. The ACC, F1 and MCC were listed in S13, S14 and S15 Tables respectively.

**Table 3:** Comparison of prediction performance of different methods on 37 circRNA datasets. Bold data represent the best AUC values of experimental results. The AUCs of the other seven methods are obtained from references [19–21].

Dataset37	iCRBP-LKHA	ASCRB	iCircRBP-DHN	PASSION	CRIP	CSCRites	CircSLNN	CRBPDL
AGO1	**0.9431±0.001**	0.9417	0.8981±0.003	0.909±0.003	0.905±0.002	0.851±0.002	0.844±0.002	0.9232
AGO2	**0.8772±0.001**	0.8601	0.7972±0.004	0.822±0.003	0.811±0.001	0.755±0.127	0.715±0.013	0.8233
AGO3	**0.9771±0.003**	0.969	0.9201±0.016	0.909±0.008	0.895±0.002	0.820±0.004	0.860±0.006	0.9472
ALKBH5	**0.9961±0.001**	0.9921	0.9791±0.004	0.752±0.030	0.721±0.008	0.798±0.009	0.583±0.014	0.9952
AUF1	0.9871±0.001	**0.9877**	0.9852±0.002	0.979±0.003	0.980±0.000	0.940±0.001	0.971±0.001	0.9810
C17ORF85	**0.9912±0.001**	0.9883	0.9873±0.002	0.860±0.021	0.813±0.007	0.815±0.012	0.721±0.010	0.9881
C22ORF28	0.9291±0.003	**0.9361**	0.9131±0.004	0.894±0.008	0.876±0.002	0.878±0.002	0.797±0.001	0.9088
CAPRIN1	**0.9271±0.001**	0.9171	0.8582±0.012	0.860±0.009	0.843±0.003	0.827±0.002	0.745±0.008	0.8765
DGCR8	**0.9542±0.002**	0.9307	0.9065±0.002	0.917±0.002	0.914±0.001	0.870±0.002	0.846±0.002	0.9236
EIF4A3	**0.8651±0.005**	0.8576	0.7994±0.003	0.823±0.004	0.812±0.001	0.820±0.001	0.717±0.005	0.853
EWSR1	**0.9571±0.003**	0.9563	0.9421±0.004	0.938±0.006	0.936±0.001	0.882±0.002	0.906±0.002	0.9443
FMRP	**0.9421±0.001**	0.9319	0.8922±0.002	0.900±0.002	0.898±0.001	0.890±0.001	0.826±0.003	0.8966
FOX2	**0.9772±0.003**	0.9675	0.9583±0.005	0.830±0.034	0.815±0.006	0.755±0.010	0.602±0.033	0.9618
FUS	0.8771±0.001	**0.8991**	0.8551±0.004	0.859±0.002	0.858±0.002	0.799±0.003	0.770±0.003	0.8618
FXR1	**0.9964±0.003**	0.9945	0.9942±0.001	0.959±0.009	0.952±0.003	0.871±0.003	0.942±0.004	0.9948
FXR2	**0.9712±0.002**	0.9688	0.9391±0.009	0.941±0.003	0.938±0.002	0.868±0.002	0.896±0.004	0.9518
HNRNPC	**0.9831±0.003**	0.9799	0.9775±0.001	0.976±0.001	0.972±0.000	0.973±0.001	0.970±0.001	0.9771
HUR	**0.9201±0.013**	0.9109	0.8674±0.005	0.879±0.006	0.874±0.001	0.850±0.001	0.796±0.009	0.8758
IGF2BP1	**0.9041±0.003**	0.9028	0.8431±0.002	0.845±0.003	0.843±0.001	0.835±0.003	0.760±0.009	0.8554
IGF2BP2	0.8551±0.003	**0.9254**	0.8314±0.004	0.827±0.009	0.821±0.002	0.752±0.126	0.740±0.004	0.8426
IGF2BP3	**0.8812±0.001**	0.9137	0.8163±0.004	0.831±0.003	0.822±0.002	0.754±0.122	0.706±0.003	0.8229
LIN28A	**0.9127±0.004**	0.8919	0.8571±0.007	0.875±0.005	0.865±0.001	0.840±0.002	0.777±0.003	0.8751
LIN28B	**0.9311±0.012**	0.8627	0.8922±0.004	0.889±0.005	0.882±0.001	0.758±0.129	0.822±0.003	0.9014
METTL3	0.8821±0.011	**0.9137**	0.8521±0.009	0.878±0.010	0.854±0.003	0.808±0.003	0.772±0.007	0.8649
MOV10	**0.9012±0.022**	0.8919	0.8381±0.006	0.845±0.005	0.849±0.001	0.778±0.004	0.777±0.008	0.8674
PTB	**0.8713±0.015**	0.8627	0.8221±0.006	0.829±0.004	0.826±0.001	0.692±0.157	0.738±0.007	0.8347
PUM2	0.9813±0.012	**0.9843**	0.9703±0.004	0.952±0.004	0.953±0.001	0.936±0.001	0.932±0.002	0.9758
QKI	**0.9911±0.006**	0.9883	0.9714±0.006	0.927±0.005	0.921±0.003	0.866±0.004	0.866±0.007	0.9879
SFRS1	**0.9821±0.006**	0.9799	0.9641±0.000	0.965±0.003	0.964±0.001	0.963±0.001	0.926±0.003	0.9684
TAF15	**0.9972±0.002**	0.9964	0.9921±0.002	0.967±0.002	0.965±0.001	0.941±0.002	0.968±0.002	0.9945
TDP43	**0.9772±0.003**	0.9443	0.9261±0.002	0.934±0.002	0.930±0.001	0.923±0.001	0.896±0.003	0.9336
TIA1	**0.9812±0.009**	0.9710	0.9612±0.004	0.935±0.006	0.932±0.003	0.915±0.009	0.901±0.003	0.9666
TIAL1	0.9381±0.001	**0.9401**	0.9173±0.003	0.906±0.003	0.902±0.001	0.898±0.002	0.871±0.005	0.9249
TNRC6	**0.9851±0.002**	0.9803	0.9674±0.002	0.785±0.010	0.741±0.007	0.729±0.010	0.662±0.015	0.9797
U2AF65	**0.9961±0.002**	0.9435	0.9261±0.002	0.930±0.002	0.928±0.001	0.911±0.001	0.899±0.004	0.9306
WTAP	**0.9831±0.004**	0.9723	0.9671±0.002	0.794±0.069	0.793±0.011	0.808±0.022	0.732±0.009	0.9713
ZC3H7B	0.8451±0.008	**0.8656**	0.8042±0.003	0.804±0.005	0.792±0.002	0.794±0.004	0.697±0.008	0.8151
**AVG**	**0.9424±0.003**	0.9385±0.044	0.9081±0.006	0.876±0.007	0.842±0.007	0.884±0.006	0.809±0.01	0.9188±0.057

As shown in Table 3, the average AUCs of iCRBP-LKHA, ASCRB, iCircRBP-DHN, PASSION, CRIP, CSCRites, CircSLNN and CRBPDL are 0.9424, 0.9385, 0.9081, 0.876, 0.842, 0.884, 0.809 and 0.9188, respectively. In terms of AUC, iCRBP-LKHA outperforms other competing methods. In 29 of the 37 datasets, our model iCRBP-LKHA achieved the highest AUC value, improving the performance of the prediction method. There is a small gap between iCRBP-LKHA and ASCRB on the other eight datasets, especially iCRBP-LKHA is slightly worse than ASCRB on four data sets (AUF1, C22ORF28, PUM2 and TIAL1). The possible reason is ASCRB obtains useful multi-view features on these eight datasets. Besides, predicting the location of binding sites can improve model performance. In ACC, F1 and MCC, iCRBP-LKHA outperforms other competing methods, demonstrating the advantages of iCRBP-LKHA over competing methods.

### The generalizability performance of methods

To evaluate the generalizability performance of methods, we trained these models (iCRBP-LKHA, ASCRB, iCircRBP-DHN, PASSION, CRIP, CSCRites, CircSLNN and CRBPDL) on one dataset and tested the capabilities of these models on the other dataset. We constructed a training dataset and an independent testing dataset using 37 circRNAs datasets. The training dataset consists of 26 circRNAs datasets, and the number of samples is 537698 (268849 positive samples and 268849 negative samples), which is about 80% of the total number of samples. The testing dataset includes the remaining 11 datasets (67127 positive samples and 67127 negative samples). The circRNA names in training and testing datasets were listed in S16 Table.

As shown in Table 4, in terms of four evaluation metrics, iCRBP-LKHA outperforms other competing methods. The results show that iCRBP-LKHA has excellent generalization capacity.

**Table 4:** Performance comparison of different methods on independent datasets. Bold data represent the best values of experimental results.

	iCRBP-LKHA	ASCRB	iCircRBP-DHN	PASSION	CRIP	CSCRites	CircSLNN	CRBPDL
AUC	**0.9048**	0.9018	0.8717	0.8492	0.8415	0.8087	0.7772	0.8822
ACC	**0.8917**	0.8716	0.8399	0.8189	0.8127	0.7817	0.7492	0.8522
F1	**0.8553**	0.8291	0.8027	0.7791	0.7726	0.7442	0.7149	0.8136
MCC	**0.7315**	0.6979	0.6747	0.6586	0.6515	0.6294	0.6026	0.6848

### The prediction performance on 37 circRNAs stringent datasets

**Fig 6:**
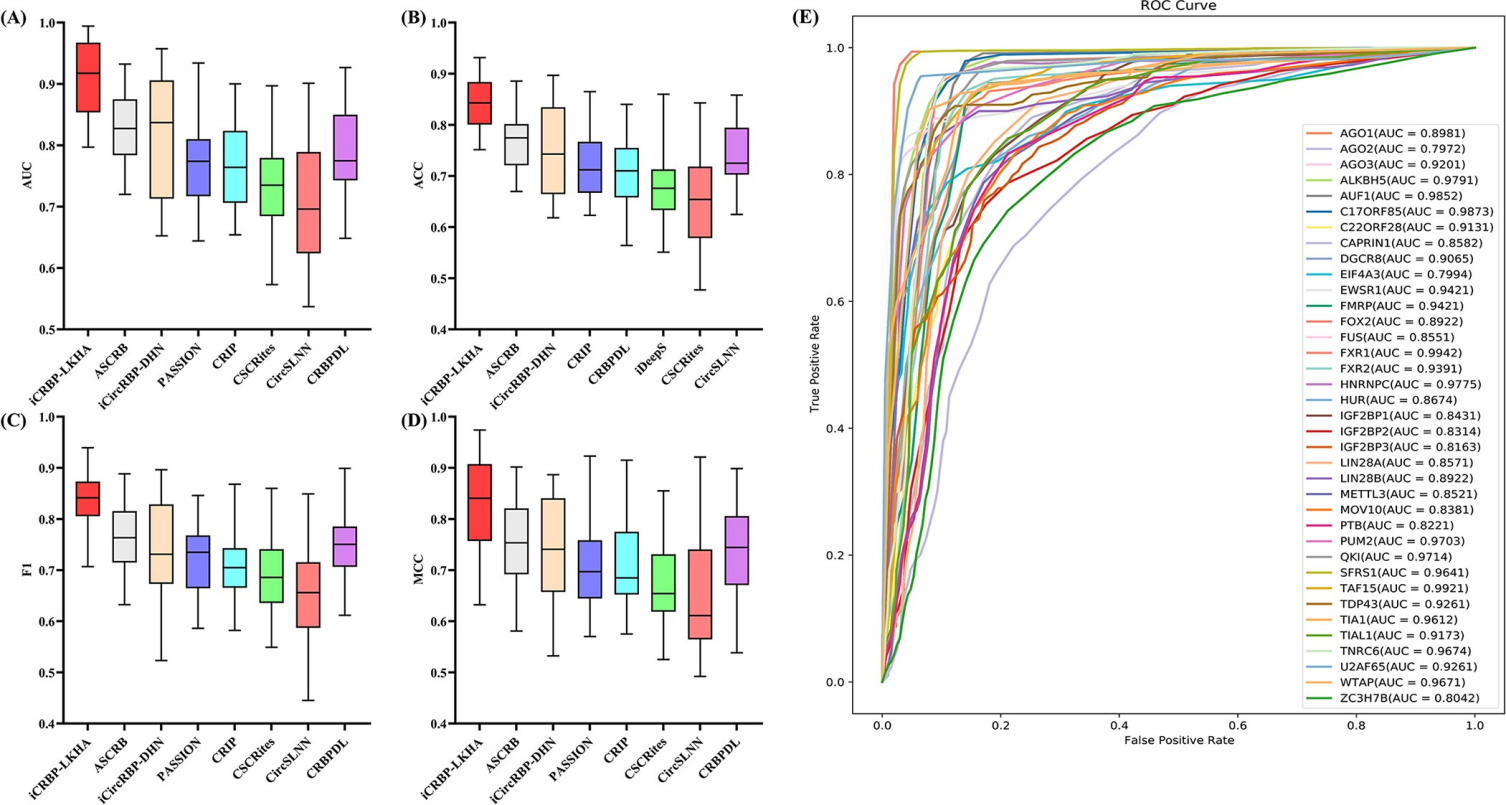
(A) Performance comparison of iCRBP-LKHA with state-of-the-art DL-based methods in terms of distribution of AUCs across 37 circRNAs stringent datasets. (B) ACCs across 37 circRNAs stringent datasets. (C) F1s across 37 circRNAs stringent datasets. (D) MCCs across 37 circRNAs stringent datasets. (E) The ROC curves of iCRBP-LKHA on 37 circRNAs stringent dataset.

To evaluate the performance of iCRBP-LKHA on a more stringent dataset, we compared the performance of iCRBP-LKHA with ASCRB, CRBPDL, iCircRBP-DHN, PASSION, CRIP, CSCRites and CircSLNN using the 37 circRNA stringent datasets. The results were shown in Fig 6A–6D. The ROC curves of iCRBP-LKHA were shown in Fig 6E. The AUC were listed in Table 5 and the last row is the average AUC. The ACC, F1 and MCC were listed in S17, S18 and S19 Tables respectively.

**Table 5:** Comparison of AUC of different methods on 37 circRNAs stringent datasets. Bold data represent the best AUC values of experimental results.

Dataset37	iCRBP-LKHA	ASCRB	iCircRBP-DHN	PASSION	CRIP	CSCRites	CircSLNN	CRBPDL
AGO1	**0.8981±0.004**	0.8356	0.8034±0.002	0.775±0.003	0.792	0.77	0.741±0.001	0.7809±0.001
AGO2	**0.7972±0.001**	0.7096	0.6276±0.004	0.65±0.003	0.638	0.592	0.576±0.002	0.6442±0.002
AGO3	**0.9201±0.001**	0.8489	0.8133±0.004	0.772±0.003	0.751	0.701	0.724±0.002	0.8306±0.002
ALKBH5	**0.9791±0.004**	0.8773	0.9095±0.001	0.654±0.002	0.677	0.719	0.499±0.001	0.921±0.004
AUF1	**0.9852±0.002**	0.8924	0.8736±0.004	0.936±0.003	0.858	0.885	0.887±0.004	0.8518±0.001
C17ORF85	**0.9873±0.002**	0.9045	0.9299±0.001	0.81±0.001	0.756	0.743	0.673±0.002	0.9334±0.001
C22ORF28	**0.9131±0.004**	0.8268	0.8154±0.003	0.802±0.003	0.75	0.768	0.717±0.004	0.8282±0.003
CAPRIN1	**0.8582±0.003**	0.7538	0.7343±0.004	0.716±0.001	0.736	0.707	0.622±0.001	0.7644±0.001
DGCR8	**0.9065±0.004**	0.7878	0.7787±0.003	0.788±0.002	0.781	0.762	0.735±0.003	0.8073±0.003
EIF4A3	**0.7994±0.004**	0.7294	0.6767±0.003	0.69±0.002	0.685	0.667	0.623±0.002	0.6884±0.002
EWSR1	**0.9421±0.004**	0.8202	0.8783±0.004	0.875±0.003	0.81	0.815	0.778±0.004	0.8436±0.003
FMRP	**0.8922±0.003**	0.7663	0.7301±0.004	0.787±0.003	0.793	0.797	0.734±0.004	0.7536±0.001
FOX2	**0.9583±0.001**	0.8903	0.8155±0.001	0.774±0.002	0.737	0.649	0.513±0.004	0.8502±0.002
FUS	**0.8551±0.003**	0.8293	0.7441±0.003	0.788±0.002	0.773	0.686	0.681±0.001	0.7979±0.002
FXR1	**0.9942±0.002**	0.9359	0.8738±0.001	0.826±0.002	0.89	0.826	0.837±0.001	0.8688±0.002
FXR2	**0.9391±0.002**	0.8458	0.8192±0.001	0.802±0.004	0.809	0.8	0.792±0.004	0.8652±0.002
HNRNPC	**0.9775±0.003**	0.9322	0.9242±0.003	0.884±0.003	0.842	0.909	0.879±0.002	0.8966±0.004
HUR	**0.8674±0.004**	0.7986	0.7067±0.001	0.77±0.004	0.783	0.747	0.665±0.002	0.7863±0.001
IGF2BP1	**0.8431±0.002**	0.7341	0.6887±0.003	0.706±0.002	0.746	0.712	0.61±0.004	0.7542±0.004
IGF2BP2	**0.8314±0.004**	0.7886	0.708±0.004	0.709±0.001	0.688	0.664	0.659±0.004	0.7523±0.003
IGF2BP3	**0.8163±0.002**	0.7842	0.7154±0.004	0.677±0.002	0.657	0.619	0.585±0.003	0.6743±0.002
LIN28A	**0.8571±0.002**	0.748	0.7066±0.002	0.727±0.002	0.776	0.739	0.696±0.002	0.7398±0.004
LIN28B	**0.8922±0.004**	0.793	0.7431±0.001	0.744±0.002	0.739	0.644	0.738±0.002	0.7486±0.001
METTL3	**0.8521±0.004**	0.7856	0.7718±0.001	0.742±0.004	0.763	0.7	0.675±0.003	0.7441±0.001
MOV10	**0.8381±0.003**	0.7881	0.7229±0.001	0.733±0.003	0.714	0.651	0.642±0.003	0.7664±0.002
PTB	**0.8221±0.004**	0.7177	0.7083±0.003	0.739±0.003	0.712	0.597	0.652±0.001	0.7±0.003
PUM2	**0.9703±0.003**	0.9286	0.9125±0.004	0.843±0.001	0.857	0.85	0.836±0.004	0.8484±0.001
QKI	**0.9714±0.002**	0.9275	0.9074±0.002	0.861±0.001	0.832	0.81	0.767±0.001	0.928±0.004
SFRS1	**0.9641±0.004**	0.8854	0.824±0.003	0.897±0.002	0.859	0.84	0.789±0.002	0.8512±0.003
TAF15	**0.9921±0.002**	0.9328	0.929±0.001	0.836±0.004	0.912	0.858	0.911±0.004	0.9412±0.003
TDP43	**0.9261±0.001**	0.8541	0.8281±0.001	0.77±0.001	0.836	0.788	0.809±0.002	0.8104±0.004
TIA1	**0.9612±0.003**	0.8193	0.8682±0.002	0.814±0.002	0.835	0.774	0.76±0.001	0.8267±0.001
TIAL1	**0.9173±0.001**	0.8217	0.8086±0.003	0.793±0.002	0.804	0.781	0.76±0.001	0.8397±0.002
TNRC6	**0.9674±0.003**	0.8541	0.8192±0.004	0.675±0.001	0.632	0.658	0.613±0.001	0.9163±0.003
U2AF65	**0.9261±0.002**	0.8067	0.7444±0.003	0.764±0.004	0.807	0.808	0.791±0.003	0.8036±0.002
WTAP	**0.9671±0.001**	0.834	0.8268±0.004	0.687±0.004	0.705	0.736	0.62±0.004	0.8634±0.002
ZC3H7B	**0.8042±0.004**	0.7305	0.6915±0.001	0.676±0.004	0.671	0.662	0.582±0.003	0.7338±0.001
**AVG**	**0.9079±0.003**	0.8259±0.044	0.8138±0.006	0.773±0.007	0.771±0.007	0.738±0.006	0.711±0.01	0.7928±0.057

As shown in Table 5, the average values of iCRBP-LKHA, ASCRB, iCircRBP-DHN, PASSION, CRIP, CSCRites, CircSLNN and CRBPDL are 0.9079, 0.8259, 0.8138, 0.773, 0.771, 0.738, 0.711 and 0.7928, respectively. All methods suffer from performance drop when encounter a stringent dataset. iCRBP-LKHA still outperforms other competing methods in a stringent dataset. iCRBP-LKHA achieved the highest AUC value on all datasets. The average ACC of iCRBP-LKHA is 0.8413, which is 9.4% higher than the highest value (ASCRB, 0.7686) among competing methods. Similarly, the average F1 of iCRBP-LKHA is 0.8401, which is 10.1% higher than 0.7632 (ASCRB), and 0.8335 (iCRBP-LKHA’s MCC) is 10.4% higher than 0.7549 (ASCRB), demonstrating the advantages of iCRBP-LKHA over competing methods on a stringent dataset.

### The prediction performance on 31 linear RNAs datasets

**Fig 7:**
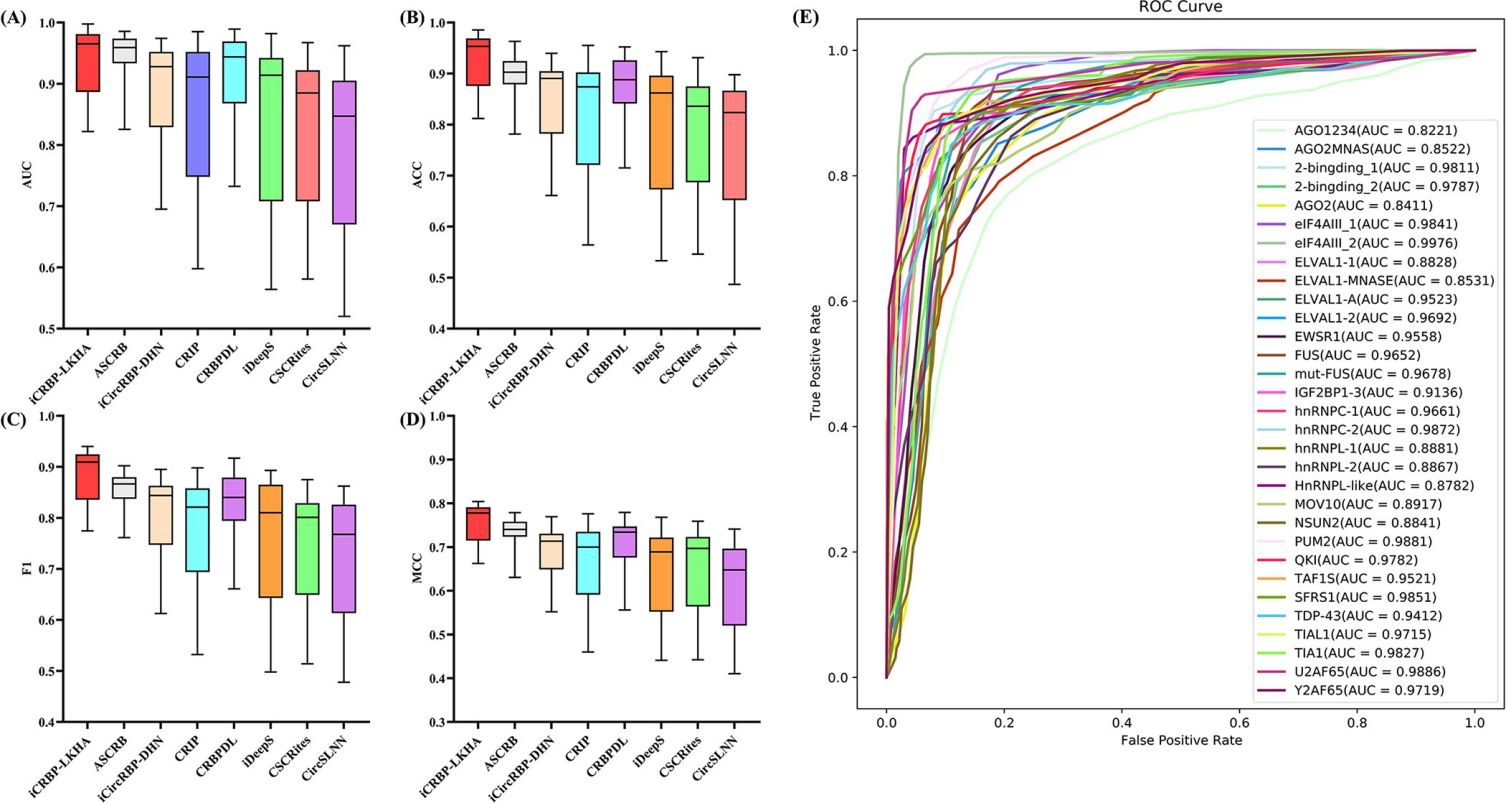
(A) Performance comparison of iCRBP-LKHA with state-of-the-art DL-based methods in terms of distribution of AUCs across 31 linear RNAs datasets. (B) ACCs across 31 linear RNAs datasets. (C) F1s across 31 linear RNAs datasets. (D) MCCs across 31 linear RNAs datasets. (E) The ROC curves of iCRBP-LKHA on 31 linear RNAs datasets.

CircRNA-RBP interaction identification methods are generally able to identify linear RNA-RBP interaction sites. To assess the effectiveness of iCRBP-LKHA in identifying linear RNA-RBP interaction sites, we compared iCRBP-LKHA with ASCRB [21], CRBPDL [20], iCircRBP-DHN [19], CRIP [16], CSCRites [35], CircSLNN [17] and iDeepS [36] using 31 benchmark datasets of linear RNAs. iDeepS is a linear RNAs-RBP interaction prediction method that integrates both sequence and secondary structure information. The results were shown in Fig 7A–7D. The ROC curves of iCRBP-LKHA were shown in Fig 7E. The AUC were listed in Table 6 and the last row is the average AUC. The ACC, F1 and MCC were listed in S20, S21 and S22 Tables respectively.

**Table 6:** Comparison of prediction performance of different methods on 31 linear RNAs datasets. Bold data represent the best AUC values of experimental results. The AUCs of the other seven methods are obtained from references [19–21].

Dataset31	iCRBP-LKHA	ASCRB	iCircRBP-DHN	CRIP	CRBPDL	iDeepS	CSCRites	CircSLNN
AGO1234	0.8221±0.005	**0.9334**	0.788±0.041	0.737±0.005	0.8178	0.708	0.708±0.004	0.662±0.015
AGO2MNAS	**0.8522±0.007**	0.8417	0.736±0.069	0.598±0.009	0.7995	0.564	0.583±0.005	0.557±0.007
2-bingding_1	**0.9811±0.006**	0.9737	0.925±0.011	0.862±0.004	0.9353	0.788	0.842±0.003	0.795±0.006
2-bingding_2	**0.9787±0.004**	0.9772	0.929±0.014	0.852±0.007	0.9439	0.831	0.828±0.003	0.754±0.004
AGO2	0.8411±0.007	0.8254	0.800±0.010	0.638±0.006	**0.9088**	0.639	0.636±0.005	0.562±0.017
eIF4AIII_1	**0.9841±0.005**	0.9803	0.963±0.004	0.952±0.001	0.9753	0.942	0.937±0.004	0.894±0.005
eIF4AIII_2	**0.9976±0.006**	0.9820	0.963±0.006	0.954±0.001	0.9851	0.945	0.944±0.002	0.897±0.006
ELVAL1-1	0.8828±0.005	**0.9598**	0.939±0.006	0.918±0.002	0.9482	0.914	0.910±0.001	0.882±0.005
ELVAL1-MNASE	**0.8531±0.013**	0.8428	0.695±0.050	0.604±0.007	0.7325	0.567	0.581±0.010	0.520±0.13
ELVAL1-A	**0.9523±0.006**	0.9486	0.922±0.006	0.898±0.002	0.9377	0.888	0.876±0.002	0.845±0.006
ELVAL1-2	**0.9692±0.002**	0.9665	0.943±0.002	0.926±0.001	0.9482	0.937	0.925±0.001	0.898±0.002
EWSR1	**0.9558±0.004**	0.9448	0.918±0.005	0.912±0.003	0.9217	0.919	0.884±0.001	0.851±0.004
FUS	**0.9652±0.007**	0.9591	0.947±0.007	0.941±0.001	0.9596	0.934	0.907±0.002	0.905±0.007
mut-FUS	**0.9678±0.003**	0.9643	0.946±0.006	0.939±0.001	0.9621	0.938	0.907±0.002	0.907±0.013
IGF2BP1-3	**0.9136±0.002**	0.8979	0.781±0.031	0.693±0.005	0.8156	0.691	0.703±0.005	0.597±0.013
hnRNPC-1	0.9661±0.001	**0.9723**	0.952±0.009	0.963±0.001	0.9514	0.966	0.936±0.004	0.935±0.004
hnRNPC-2	**0.9872±0.001**	0.9821	0.974±0.002	0.985±0.001	0.9891	0.982	0.967±0.002	0.962±0.001
hnRNPL-1	**0.8881±0.001**	0.8842	0.829±0.032	0.748±0.005	0.8583	0.659	0.650±0.007	0.670±0.011
hnRNPL-2	**0.8867±0.004**	0.8587	0.761±0.027	0.740±0.007	0.7995	0.671	0.636±0.004	0.654±0.014
HnRNPL-like	**0.8782±0.007**	0.8603	0.779±0.021	0.685±0.010	0.8008	0.644	0.632±0.010	0.636±0.014
MOV10	0.8917±0.008	**0.9339**	0.885±0.010	0.814±0.002	0.8971	0.807	0.803±0.003	0.764±0.010
NSUN2	0.8841±0.005	**0.9365**	0.832±0.008	0.865±0.003	0.8681	0.789	0.798±0.004	0.776±0.015
PUM2	**0.9881±0.004**	0.9801	0.969±0.003	0.963±0.003	0.9782	0.966	0.959±0.002	0.920±0.004
QKI	**0.9782±0.004**	0.9669	0.962±0.002	0.967±0.001	0.9786	0.972	0.956±0.001	0.929±0.004
SFRS1	**0.9521±0.001**	0.9471	0.912±0.007	0.886±0.004	0.9236	0.888	0.885±0.003	0.794±0.008
TAF1S	**0.9851±0.002**	0.9765	0.971±0.002	0.963±0.001	0.9336	0.961	0.922±0.004	0.925±0.002
TDP-43	0.9412±0.009	**0.9517**	0.928±0.013	0.911±0.002	0.946	0.914	0.913±0.003	0.841±0.009
TIA1	**0.9715±0.005**	0.9688	0.945±0.011	0.930±0.001	0.9636	0.916	0.891±0.001	0.894±0.005
TIAL1	**0.9827±0.009**	0.9469	0.915±0.012	0.898±0.002	0.9799	0.885	0.864±0.004	0.847±0.009
U2AF65	**0.9886±0.003**	0.9855	0.971±0.007	0.968±0.001	0.9782	0.965	0.918±0.002	0.932±0.003
Y2AF65	**0.9719±0.002**	0.9702	0.951±0.005	0.935±0.002	0.969	0.927	0.906±0.004	0.893±0.002
**Avg**	**0.9374±0.003**	0.9393±0.047	0.895±0.08	0.860±0.12	0.9163±0.068	0.842±0.13	0.833±0.12	0.803±0.13

As shown in Table 6, the average AUCs of iCRBP-LKHA, ASCRB, iCircRBP-DHN, CRIP, CRBPDL, iDeepS, CSCRites and CircSLNN are 0.9374, 0.9393, 0.895, 0.860, 0.9163, 0.842, 0.833 and 0.803, respectively. On the dataset AGO1234, the AUC of ASCRB is higher than that of iCRBP-LKHA, but in the remaining 30 datasets, the average AUC of iCRBP-LKHA is higher than that of ASCRB (0.9412 vs. 0.9395). In 24 of the 31 datasets, our model iCRBP-LKHA achieved the highest AUC value, improving the performance of state-of-the-art prediction methods. On the dataset hnRNPC-1 and TDP-43, iCRBP-LKHA is slightly worse than ASCRB. In terms of AUC, iCRBP-LKHA outperforms other competing methods, the same was found in ACC, F1 and MCC. The above results indicate that iCRBP-LKHA is better than competing methods in predicting linear RNA-RBP interaction sites.

## Discussion

In this paper, we proposed a novel DL-based model, iCRBP-LKHA, based on large convolutional kernel and hybrid channel-spatial attention for identifying circRNA-RBP interaction sites. To effectively extract features from sequences, we adopted five encoding schemes, including KNF, Doc2Vec, EIIP, CCN and ANF to extract comprehensive feature information. Meanwhile, the neural network architecture, which consists of LKCNN, CBAM-1D and BiGRU, was proposed to explore local information, global context information and multiple features interaction information automatically. By integrating multiple information, iCRBP-LKHA improved model performance as compared with several state-of-the-art methods. The experimental results on 37 circRNAs datasets, 37 circRNAs stringent datasets and 31 linear RNAs datasets not only demonstrate the effectiveness of iCRBP-LKHA but also demonstrate the potential of this model in identifying other RNA-RBP interaction sites.

## Materials and methods

### Data preparation

To evaluate the prediction performance of iCRBP-LKHA, we worked with the 37 circRNAs datasets (https://github.com/kavin525zhang/CRIP) that have been widely used by DL-based algorithms for benchmarking their performances [16,18–20]. CD-HIT was used to eliminate redundant sequences with the sequence identity threshold of 80% [37]. A total of 32,216 circRNAs were obtained from 37 circRNAs datasets. The wet lab-verified verified interaction sites were treated as positive samples and negative samples of equal size were randomly selected from the remaining fragments. 335,976 positive samples and 335,976 negative samples were used to evaluate the model performance. To observe performance of the method on a more stringent dataset, CD-HIT was used to eliminate redundant sequences with the sequence identity threshold of 60%, resulting in a total of 139,293 positive samples and 139,293 negative samples (https://github.com/nathanyl/iCRBP-LKHA). The dataset was named 37 circRNAs stringent datasets. The number of samples in the 37 circRNAs datasets and the 37 circNRAs stringent datasets were listed in Table 7. 80% of the samples were randomly selected as the training set, and the remaining 20% of the samples were used as the testing set. 10-flod cross validation were applied to optimize the parameters.

**Table 7:** The number of samples associated with each circRNA.

37 circRNAs datasets	Sample Number	37 circRNAs stringent datasets	Sample Number
AGO1	17318	AGO1	6718
AGO2	20000	AGO2	8211
AGO3	3124	AGO3	2211
ALKBHS	770	ALKBHS	670
AUF1	2896	AUF1	1399
C17ORF85	1016	C17ORF85	819
C22ORF28	5292	C22ORF28	3077
CAPRIN1	5298	CAPRIN1	3467
DGCR8	20000	DGCR8	7000
EIF4A3	20000	EIF4A3	7901
EWSR1	4695	EWSR1	2637
FMRP	20000	FMRP	6975
FOX2	605	FOX2	550
FUS	20000	FUS	7453
FXR1	927	FXR1	723
FXR2	5635	FXR2	3075
HNRNPC	14224	HNRNPC	1571
HUR	20000	HUR	7199
IGF2BP1	20000	IGF2BP1	7386
IGF2BP2	10000	IGF2BP2	5164
IGF2BP3	20000	IGF2BP3	7867
LIN28A	18277	LIN28A	7536
LIN28B	7888	LIN28B	4288
METTL3	2666	METTL3	1356
MOV10	5889	MOV10	3358
PTB	20000	PTB	7879
PUM2	2329	PUM2	1185
QKI	1033	QKI	738
SFRS1	8595	SFRS1	3450
TAF15	1467	TAF15	983
TDP43	5484	TDP43	1901
TIA1	2202	TIA1	1184
TIAL1	5456	TIAL1	2533
TNRC6	1101	TNRC6	913
U2AF65	8224	U2AF65	3034
WTAP	446	WTAP	308
ZC3H7B	13119	ZC3H7B	6574
**Total number**	**335976**		**139293**

Additionally, we compared the performance of iCRBP-LKHA with state-of-the-art linear RNA-RBP interaction sites identification methods. The benchmark human datasets of 31 linear RNAs were collected by iONMF [38] and downloaded from https://github.com/mstrazar/ionmf. Each dataset consists of 5000 training samples and 1000 testing samples.

### The framework of iCRBP-LKHA

Traditional CNNs usually use small-sized convolutional kernels, such as 1x1, 3x3 and 5x5. However, small convolutional kernels may not effectively capture long-distance dependencies in sequence data. Compared with small convolutional kernels, large convolutional kernels can increase the effective receptive field (ERF) [39] by increasing the kernel width and height, thereby better capturing long-distance dependencies [23]. Traditional attention mechanisms are usually implemented by learning weights, such as using the softmax function. In contrast, the hybrid attention mechanism can simultaneously consider multiple attention mechanisms and their combinations to obtain more comprehensive feature information from the data [40]. CBAM is a simple yet effective attention module [24].

Inspired by the large convolutional kernels and hybrid attention mechanism, we designed a novel DL-based method namely iCRBP-LKHA for predicting circRNA-RBP interaction sites. As shown in Fig 1, iCRBP-LKHA adopts five encoding schemes. The neural network architecture of iCRBP-LKHA mainly includes LKCNN, CBAM-1D and BiGRU.

### Feature encoding

In this section, all fragments are encoded into five different types of features, including KNF, Doc2Vec, EIIP, CCN and ANF. These encoding schemes can extract various feature information from sequences.

### k-nucleotide frequency (KNF)

KNF was used to extract local contextual features from circRNA sequences. KNF describes the frequency of all possible polynucleotides of *k* nucleotides occurring in the sequence. KNF integrates various local sequence information while preserving a large amount of original sequence information [41]. Compared with the traditional one-hot encoding [16], KNF can retain more effective information in the sequence. The *k* nucleotides refer to all of a sequence’s subsequences of length *k*, such that the sequence ACGU would have four 1-nucleotide (A, C, G and T), three 2-nucleotides (AC, CG, GT), two 3-nucleotides (ACG and CGT) and one 4-nucleotides (ACGT). In this paper, we set *k* = 1, 2, 3, which are called single-nucleotide composition frequency, dinucleotide composition frequency and trinucleotide composition frequency respectively. A sequence of length *L* will have *L*-*k*+1 *k*-nucleotides and 4^*k*^ total possible *k*-nucleotides.

### Doc2Vec

In order to extract more sequence context and high-order biological information, a continuous high-dimensional word embedding encoding method Doc2Vec was used to vectorize the sequence and train the vectorization model [42]. The 10-mer sequence fragments were input into the model, and the feature vectors were obtained through word embedding training. Doc2Vec captures the continuous distribution of global contextual features and semantic information to model long-term dependencies in sequences.

### Electron–ion interaction pseudopotential (EIIP)

EIIP calculates the characteristics of free electron energy. These free electron energy is consider to be related to the binding site interaction [20,22]. The EIIP values of sequence ATGC are 0.1260, 0.1335, 0.0806 and 0.1340 respectively. For example, TACCGAA is encoded as a numeric vector (0.1335, 0.1260, 0.1340, 0.1340, 0.0806, 0.1260, 0.1260). We used the EIIP encoding method to encode DNA sequences into digital vectors.

### Chemical characteristic of nucleotide (CCN)

Each nucleotide contains three chemical features (CCN), which are ring structure, chemical functions and hydrogen bonds. Research shows that these three chemical features are related to binding site interactions [43]. In ring structure, A and G are coded as 1, C and T are coded as 0. In chemical functions, A and C are coded as 1, G and T are coded as 0. In hydrogen bonds, A and T are coded as 1, C and G are coded as 0. For example, GTACCGA is encoded as (1, 0, 0, 0, 0, 0, 1, 1, 1, 0, 1, 0, 0, 1, 0, 1, 0, 0, 1, 1, 1).

### Accumulated nucleotide frequency (ANF)

ANF describes the occurrence frequency of the *i*-th nucleotide in a fragment composed of previous *i*-nucleotides and is widely used to represent the density feature of nucleotide sequences [44]. ANF can be used to identify sequence features [45]. The density *d*_*i*_ of any nucleotide *s*_*i*_ at position *i* can be defined by the following formula,

$$d_{i}=\frac{1}{\left|S_{i}\right|}\sum_{j=1}^{L}f(s_{j}),\;f(s_{j})=\{\begin{array}{l} 1,\;if\;s_{j}=q\\ 0,\;other\;cases \end{array}$$ (1)

where *L* is the sequence length, |*S*_*i*_| is the length of the *i*-th prefix string {*s*_*1*_, *s*_*2*_, …, *s*_*i*_} in the sequence, q∈{A, C, G, T}.

### Deep neural network architecture

A deep neural network architecture is proposed to extract important local and global information from five encoding schemes. The model architecture shown in Fig 1 mainly consists of three parts, namely LKCNN, CBAM-1D and BiGRU network.

### Large kernel convolutional neural network (LKCNN)

Compared with small convolutional kernels, large convolutional kernels can increase the ERF and capture more complex patterns and nonlinear relationships, thereby improving the performance of neural networks [23,46]. In this paper, for five feature matrices obtained by five encoding schemes, a large kernel CNN was used to reparametrize the feature matrices to help downstream feature extraction task.

Since the distributions of the five original feature matrices are different, we first applied 128 1D convolutional filters with kernel size 3 to the original feature matrices to obtain five feature matrices of the same size. The five feature matrices were concatenated to form a new feature map. Then the feature map was fed to a 1x1 convolutional layer, followed by a 2x2 average pooling operation with a stride of 2, and the convolution kernel is 512. Subsequently, we used a 1x1 convolutional layer with 256 kernels, followed by batch normalization (BN) operation. After that, we used a convolution layer with 256 7x7 convolution kernels. Then, a 5x5 convolutional layer with 256 convolution kernels was used, followed by a max pooling operation. Finally, a 3x3 convolutional layer with a max pooling operation was used and the convolution kernel is 128, and then the feature map was fed a 1x1 convolutional layer with a convolution kernel of 128.

### Convolutional block attention module with one-dimensional convolution (CBAM-1D)

The attention mechanism is a widely used method for improving the feature representation of the model [47]. Inspired by CBAM, we proposed CBAM-1D to extract the key information of feature matrices and the correlation information between the five features.

CBAM-1D module can generate attention maps in both channel and spatial dimensions, then the two attention maps are multiplied to the original feature map for adaptive feature refinement to generate the final feature map. CBAM-1D focuses on important features and suppresses the influence of noisy data and irrelevant information.

CBAM-1D consists of two modules, a 1D-channel attention module and a spatial attention module. In the channel attention module, first, the feature map passed through global max pooling and 1D-global average pooling respectively, and passed through multilayer perceptron (MLP) respectively. 1D-global average pooling means first performing a 1D convolution operation and then performing global average pooling, which can improve the feature representation ability of the model. Then the two feature maps were merged by element-wise summation, and passed through the ReLU to generate the final channel attention feature map. Finally, the channel attention feature map and the feature map were element-wise multiplied to generate the feature map required by the spatial attention module.

In the spatial attention module, first, the feature map passed through global max pooling and global average pooling, and the two results were concated. Then after a convolution operation with kernel size 7x7, the dimension was reduced to one channel. Next, feature map generated spatial attention feature through sigmoid function. Finally, the spatial attention feature and the feature map were multiplied to obtain the final feature.

### Bidirectional gating recurrent unit (BiGRU)

In this section, BiGRU was used to extract important information in the sequence [48]. BiGRU has two gates: reset gate and update gate. The reset gate enables the model to ignore previous state information, while the update gate allows the model to incorporate the previous state into the current state when processing the sequence. By updating previous state information to the current state, the model can capture important contextual information that contributes to the final prediction. In BiGRU, the hidden unit size is set to 128, the batch size is 1024, the learning rate is 0.003, and dropout is set to 0.8.

### Evaluation methods

In the experiment, AUC, ACC, F1 and MCC are used to evaluate the performance of these methods.


$$\begin{array}{c} TPR=\frac{TP}{TP+FN}\\ FPR=\frac{FP}{FP+TN}\\ ACC=\frac{TP+TN}{TP+TN+FP+FN}\\ Sen=\frac{TP}{TP+FN}\\ MCC=\frac{TP\times TN-FP\times FN}{\sqrt{\left(TP+FP)(TP+FN)(TN+FP)(TN+FN\right)}} \end{array}$$ (2)

## Supporting information

S1 Table Performance comparison of iCRBP-LKHA, iCRBP-LKHA+2 and iCRBP-LKHA-2 on 37 circRNAs datasets. Bold data represent the best AUC values of experimental results.
(DOCX)

S2 Table Performance comparison of Fea-iCRBP-LKHA, Fea-PASSION and Fea-CRIP on 37 circRNAs datasets. Bold data represent the best AUC values of experimental results.
(DOCX)

S3 Table Performance comparison of different encoding schemes on 37 circRNAs datasets. Bold data represent the best AUC values of experimental results.
(DOCX)

S4 Table Performance comparison of network architectures on 37 circRNAs datasets. Bold data represent the best AUC values of experimental results.
(DOCX)

S5 Table Ablation experiments on 37 circRNA datasets. (DOCX)

S6 Table Comparison of ACC between iCRBP-LKHA and five shallow learning algorithms on 37 circRNA datasets. Bold data represent the best ACC values of experimental results.
(DOCX)

S7 Table Comparison of F1 between iCRBP-LKHA and five shallow learning algorithms on 37 circRNA datasets. Bold data represent the best F1 values of experimental results.
(DOCX)

S8 Table Comparison of MCC between iCRBP-LKHA and five shallow learning algorithms on 37 circRNAs datasets. Bold data represent the best MCC values of experimental results.
(DOCX)

S9 Table Comparison of AUC between iCRBP-LKHA and five shallow learning algorithms on 37 circRNAs stringent datasets. Bold data represent the best AUC values of experimental results.
(DOCX)

S10 Table Comparison of ACC between iCRBP-LKHA and five shallow learning algorithms on 37 circRNAs stringent datasets. Bold data represent the best ACC values of experimental results.
(DOCX)

S11 Table Comparison of F1 between iCRBP-LKHA and five shallow learning algorithms on 37 circRNAs stringent datasets. Bold data represent the best F1 values of experimental results.
(DOCX)

S12 Table Comparison of MCC between iCRBP-LKHA and five shallow learning algorithms on 37 circRNAs stringent datasets. Bold data represent the best MCC values of experimental results.
(DOCX)

S13 Table Comparison of ACC of different methods on 37 circRNA datasets. Bold data represent the best ACC values of experimental results.
(DOCX)

S14 Table Comparison of F1 of different methods on 37 circRNA datasets. Bold data represent the best F1 values of experimental results.
(DOCX)

S15 Table Comparison of MCC of different methods on 37 circRNA datasets. Bold data represent the best MCC values of experimental results.
(DOCX)

S16 Table The circRNA names in training and testing datasets. (DOCX)

S17 Table Comparison of ACC of different methods on 37 circRNAs stringent datasets. Bold data represent the best ACC values of experimental results.
(DOCX)

S18 Table Comparison of F1 of different methods on 37 circRNAs stringent datasets. Bold data represent the best F1 values of experimental results.
(DOCX)

S19 Table Comparison of MCC of different methods on 37 circRNAs stringent datasets. Bold data represent the best MCC values of experimental results.
(DOCX)

S20 Table Comparison of ACC of different methods on 31 linear RNAs datasets. Bold data represent the best ACC values of experimental results.
(DOCX)

S21 Table Comparison of F1 of different methods on 31 linear RNAs datasets. Bold data represent the best F1 values of experimental results.
(DOCX)

S22 Table Comparison of MCC of different methods on 31 linear RNAs datasets. Bold data represent the best MCC values of experimental results.
(DOCX)
