# Supplementary material for: iCRBP-LKHA: Large convolutional kernel and hybrid channel-spatial attention for identifying circRNA-RBP interaction sites
Source: PLoS Comput Biol. 2024 Aug 22;20(8):e1012399. doi: 10.1371/journal.pcbi.1012399 (PMC11373821; doi:10.1371/journal.pcbi.1012399)
Supplement: S1 Table — Bold data represent the best AUC values of experimental results. (DOCX) [file pcbi.1012399.s001.docx]

**Supplementary Table 1.** Performance comparison of iCRBP-LKHA, iCRBP-LKHA+2 and iCRBP-LKHA-2 on 37 circRNAs datasets. Bold data represent the best AUC values of experimental results.

|  | **iCRBP-LKHA** | **iCRBP-LKHA-2** | **iCRBP-LKHA+2** |
| --- | --- | --- | --- |
| AGO1 | **0.9431** | 0.922 | 0.9121 |
| AGO2 | **0.8772** | 0.8221 | 0.8231 |
| AGO3 | **0.9771** | 0.9311 | 0.9341 |
| ALKBH5 | **0.9961** | 0.9211 | 0.9215 |
| AUF1 | **0.9871** | 0.9512 | 0.9112 |
| C17ORF85 | **0.9910** | 0.8113 | 0.8113 |
| C22ORF28 | **0.9291** | 0.9102 | 0.9012 |
| CAPRIN1 | **0.9271** | 0.8912 | 0.8612 |
| DGCR8 | **0.9542** | 0.8541 | 0.8341 |
| EIF4A3 | **0.8651** | 0.8211 | 0.8234 |
| EWSR1 | **0.9571** | 0.9512 | 0.9215 |
| FMRP | **0.9421** | 0.8113 | 0.8912 |
| FOX2 | **0.9772** | 0.9102 | 0.8113 |
| FUS | 0.8771 | 0.8912 | **0.9001** |
| FXR1 | **0.9961** | 0.8541 | 0.9041 |
| FXR2 | **0.9712** | 0.9211 | 0.8901 |
| HNRNPC | **0.9831** | 0.9512 | 0.8234 |
| HUR | 0.9201 | 0.8113 | **0.9215** |
| IGF2BP1 | **0.9141** | 0.9102 | 0.8893 |
| IGF2BP2 | 0.8551 | **0.8912** | 0.8013 |
| IGF2BP3 | **0.8812** | 0.8541 | 0.8793 |
| LIN28A | 0.9127 | **0.9211** | 0.8612 |
| LIN28B | 0.9311 | **0.9512** | 0.8341 |
| METTL3 | **0.8821** | 0.8111 | 0.8234 |
| MOV10 | 0.9012 | 0.9102 | **0.9215** |
| PTB | 0.8713 | **0.8912** | 0.8814 |
| PUM2 | **0.9813** | 0.9211 | 0.8113 |
| QKI | **0.9911** | 0.9211 | 0.9014 |
| SFRS1 | **0.9821** | 0.9512 | 0.8612 |
| TAF15 | **0.9972** | 0.8013 | 0.8341 |
| TDP43 | **0.9772** | 0.9102 | 0.8234 |
| TIA1 | **0.9812** | 0.8912 | 0.9215 |
| TIAL1 | **0.9381** | 0.8541 | 0.8956 |
| TNRC6 | **0.9851** | 0.8641 | 0.8213 |
| U2AF65 | **0.9961** | 0.9102 | 0.8903 |
| WTAP | **0.9831** | 0.8912 | 0.8875 |
| ZC3H7B | 0.8451 | **0.8541** | 0.8341 |
| **AVG** | **0.9423** | 0.8878 | 0.8694 |
