# Supplementary material for: iCRBP-LKHA: Large convolutional kernel and hybrid channel-spatial attention for identifying circRNA-RBP interaction sites
Source: PLoS Comput Biol. 2024 Aug 22;20(8):e1012399. doi: 10.1371/journal.pcbi.1012399 (PMC11373821; doi:10.1371/journal.pcbi.1012399)
Supplement: S2 Table — Bold data represent the best AUC values of experimental results. (DOCX) [file pcbi.1012399.s002.docx]

**Supplementary Table 2.** Performance comparison of Fea-iCRBP-LKHA, Fea-PASSION and Fea-CRIP on 37 circRNAs datasets. Bold data represent the best AUC values of experimental results.

|  | **Fea-iCRBP-LKHA** | **Fea-PASSION** | **Fea-CRIP** |
| --- | --- | --- | --- |
| AGO1 | **0.9431** | 0.9072 | 0.9021 |
| AGO2 | **0.8772** | 0.8231 | 0.8131 |
| AGO3 | **0.9771** | 0.9093 | 0.8962 |
| ALKBH5 | **0.9961** | 0.7521 | 0.7191 |
| AUF1 | **0.9871** | 0.9691 | 0.9791 |
| C17ORF85 | **0.991** | 0.8612 | 0.8112 |
| C22ORF28 | **0.9291** | 0.8911 | 0.8711 |
| CAPRIN1 | **0.9271** | 0.8612 | 0.8421 |
| DGCR8 | **0.9542** | 0.9181 | 0.9042 |
| EIF4A3 | **0.8651** | 0.8131 | 0.8021 |
| EWSR1 | **0.9571** | 0.9331 | 0.9381 |
| FMRP | **0.9421** | 0.901 | 0.8971 |
| FOX2 | **0.9772** | 0.8313 | 0.8125 |
| FUS | **0.8771** | 0.8581 | 0.8528 |
| FXR1 | **0.9961** | 0.9582 | 0.9512 |
| FXR2 | **0.9712** | 0.9522 | 0.9318 |
| HNRNPC | **0.9831** | 0.9751 | 0.9712 |
| HUR | **0.9201** | 0.8891 | 0.8714 |
| IGF2BP1 | **0.9041** | 0.8551 | 0.8423 |
| IGF2BP2 | **0.8551** | 0.8371 | 0.8231 |
| IGF2BP3 | **0.8812** | 0.8312 | 0.8232 |
| LIN28A | **0.9127** | 0.8852 | 0.8635 |
| LIN28B | **0.9311** | 0.8891 | 0.8802 |
| METTL3 | **0.8821** | 0.8732 | 0.8514 |
| MOV10 | **0.9012** | 0.8454 | 0.8489 |
| PTB | **0.8713** | 0.8292 | 0.8236 |
| PUM2 | **0.9813** | 0.9511 | 0.9533 |
| QKI | **0.9911** | 0.9252 | 0.9221 |
| SFRS1 | **0.9821** | 0.9643 | 0.9624 |
| TAF15 | **0.9972** | 0.9611 | 0.9615 |
| TDP43 | **0.9772** | 0.9271 | 0.934 |
| TIA1 | **0.9812** | 0.9392 | 0.9352 |
| TIAL1 | **0.9381** | 0.9021 | 0.9052 |
| TNRC6 | **0.9851** | 0.7812 | 0.8111 |
| U2AF65 | **0.9961** | 0.9281 | 0.9228 |
| WTAP | **0.9831** | 0.7913 | 0.8323 |
| ZC3H7B | **0.8451** | 0.8015 | 0.7932 |
| **AVG** | **0.9423** | 0.8844 | 0.8772 |
