# Supplementary material for: iCRBP-LKHA: Large convolutional kernel and hybrid channel-spatial attention for identifying circRNA-RBP interaction sites
Source: PLoS Comput Biol. 2024 Aug 22;20(8):e1012399. doi: 10.1371/journal.pcbi.1012399 (PMC11373821; doi:10.1371/journal.pcbi.1012399)
Supplement: S3 Table — Bold data represent the best AUC values of experimental results. (DOCX) [file pcbi.1012399.s003.docx]

**Supplementary Table 3.** Performance comparison of different encoding schemes on 37 circRNAs datasets. Bold data represent the best AUC values of experimental results.

|  | **iCRBP-LKHA** | **iCRBP-LKHA/KNF** | **iCRBP-LKHA/Doc2Vec** | **iCRBP-LKHA/EIIP** | **iCRBP-LKHA/CCN** | **iCRBP-LKHA/ANF** |
| --- | --- | --- | --- | --- | --- | --- |
| AGO1 | **0.9431** | 0.9131 | 0.9136 | 0.9031 | 0.9141 | 0.9041 |
| AGO2 | **0.8772** | 0.8274 | 0.8226 | 0.8372 | 0.8232 | 0.8232 |
| AGO3 | **0.9771** | 0.9073 | 0.9271 | 0.9141 | 0.9174 | 0.9074 |
| ALKBH5 | **0.9961** | 0.9061 | 0.9166 | 0.9024 | 0.9263 | 0.916 |
| AUF1 | **0.9871** | 0.9176 | 0.9073 | 0.9223 | 0.9121 | 0.9074 |
| C17ORF85 | **0.9912** | 0.9133 | 0.9014 | 0.9115 | 0.9216 | 0.9411 |
| C22ORF28 | **0.9291** | 0.9012 | 0.8923 | 0.9002 | 0.8909 | 0.8921 |
| CAPRIN1 | **0.9271** | 0.8972 | 0.8927 | 0.8623 | 0.8901 | 0.8831 |
| DGCR8 | **0.9542** | 0.9146 | 0.9042 | 0.9141 | 0.9242 | 0.9125 |
| EIF4A3 | **0.8651** | 0.8121 | 0.8254 | 0.8052 | 0.8213 | 0.7951 |
| EWSR1 | **0.9571** | 0.9073 | 0.915 | 0.9079 | 0.9173 | 0.9141 |
| FMRP | **0.9421** | 0.8922 | 0.902 | 0.9014 | 0.9022 | 0.8923 |
| FOX2 | **0.9772** | 0.9136 | 0.9247 | 0.9123 | 0.9012 | 0.9251 |
| FUS | **0.8771** | 0.8233 | 0.8345 | 0.8275 | 0.8371 | 0.8022 |
| FXR1 | **0.9964** | 0.9133 | 0.9235 | 0.9411 | 0.9162 | 0.9041 |
| FXR2 | **0.9712** | 0.9125 | 0.9103 | 0.9211 | 0.9047 | 0.9321 |
| HNRNPC | **0.9831** | 0.9342 | 0.9146 | 0.9235 | 0.9237 | 0.9431 |
| HUR | **0.9201** | 0.8923 | 0.8897 | 0.8872 | 0.8794 | 0.9081 |
| IGF2BP1 | **0.9041** | 0.8842 | 0.8904 | 0.8781 | 0.8872 | 0.8924 |
| IGF2BP2 | **0.8551** | 0.8163 | 0.8248 | 0.8134 | 0.8216 | 0.8154 |
| IGF2BP3 | **0.8812** | 0.8321 | 0.8202 | 0.8314 | 0.8212 | 0.8435 |
| LIN28A | **0.9127** | 0.9022 | 0.8917 | 0.8911 | 0.8891 | 0.8915 |
| LIN28B | **0.9311** | 0.9014 | 0.8933 | 0.8961 | 0.8942 | 0.8846 |
| METTL3 | **0.8821** | 0.8224 | 0.8026 | 0.8028 | 0.8123 | 0.8012 |
| MOV10 | **0.9012** | 0.8917 | 0.8732 | 0.8622 | 0.8551 | 0.8512 |
| PTB | **0.8713** | 0.821 | 0.8013 | 0.8241 | 0.8132 | 0.8022 |
| PUM2 | **0.9813** | 0.9021 | 0.9101 | 0.9018 | 0.9022 | 0.8912 |
| QKI | **0.9911** | 0.9101 | 0.8931 | 0.8874 | 0.8921 | 0.8891 |
| SFRS1 | **0.9821** | 0.8923 | 0.8766 | 0.9029 | 0.8951 | 0.8991 |
| TAF15 | **0.9972** | 0.9092 | 0.9032 | 0.8993 | 0.8901 | 0.8896 |
| TDP43 | **0.9772** | 0.9173 | 0.9013 | 0.9312 | 0.9502 | 0.8902 |
| TIA1 | **0.9812** | 0.9035 | 0.8916 | 0.9002 | 0.8803 | 0.9102 |
| TIAL1 | **0.9381** | 0.8902 | 0.8984 | 0.8705 | 0.8662 | 0.8737 |
| TNRC6 | **0.9851** | 0.9043 | 0.9154 | 0.8352 | 0.901 | 0.9024 |
| U2AF65 | **0.9961** | 0.9363 | 0.916 | 0.8862 | 0.8985 | 0.9066 |
| WTAP | **0.9831** | 0.8935 | 0.9033 | 0.9086 | 0.9332 | 0.923 |
| ZC3H7B | **0.8451** | 0.7956 | 0.8052 | 0.7858 | 0.8025 | 0.815 |
| AVG | **0.9423** | 0.8871 | 0.8846 | 0.8811 | 0.8845 | 0.8831 |
