# Supplementary material for: iCRBP-LKHA: Large convolutional kernel and hybrid channel-spatial attention for identifying circRNA-RBP interaction sites
Source: PLoS Comput Biol. 2024 Aug 22;20(8):e1012399. doi: 10.1371/journal.pcbi.1012399 (PMC11373821; doi:10.1371/journal.pcbi.1012399)
Supplement: S4 Table — Bold data represent the best AUC values of experimental results. (DOCX) [file pcbi.1012399.s004.docx]

**Supplementary Table 4.** Performance comparison of network architectures on 37 circRNAs datasets. Bold data represent the best AUC values of experimental results.

|  | **iCRBP-LKCA** | **CRBPDL** | **CNN-LSTM** | **iDeepE** | **ResNet** | **CRIP** | **CNN-BiLSTM** |
| --- | --- | --- | --- | --- | --- | --- | --- |
| AGO1 | 0.9431 | 0.9232 | 0.8072 | **0.9576** | 0.9451 | 0.8325 | 0.8072 |
| AGO2 | **0.8772** | 0.8233 | 0.7902 | 0.7916 | 0.853 | 0.7802 | 0.7902 |
| AGO3 | **0.9771** | 0.9472 | 0.879 | 0.8709 | 0.9318 | 0.8777 | 0.879 |
| ALKBH5 | **0.9961** | 0.9952 | 0.8757 | 0.999 | 0.9617 | 0.8675 | 0.8757 |
| AUF1 | **0.9871** | 0.981 | 0.9283 | 0.9286 | 0.9865 | 0.9635 | 0.9283 |
| C17ORF85 | **0.9912** | 0.9881 | 0.9551 | 0.9587 | 0.9226 | 0.9516 | 0.9551 |
| C22ORF28 | 0.9291 | 0.9088 | 0.9091 | **0.9876** | 0.9586 | 0.9092 | 0.9091 |
| CAPRIN1 | **0.9271** | 0.8765 | 0.7577 | 0.8945 | 0.9555 | 0.7506 | 0.7577 |
| DGCR8 | 0.9542 | 0.9236 | 0.9052 | **0.9638** | 0.9235 | 0.8972 | 0.9052 |
| EIF4A3 | **0.8651** | 0.853 | 0.7534 | 0.7912 | 0.79 | 0.7593 | 0.7634 |
| EWSR1 | **0.9571** | 0.9443 | 0.8971 | 0.9151 | 0.9234 | 0.8988 | 0.8971 |
| FMRP | **0.9421** | 0.8966 | 0.8534 | 0.8121 | 0.8276 | 0.8443 | 0.8534 |
| FOX2 | **0.9772** | 0.9618 | 0.9156 | 0.8751 | 0.8751 | 0.9153 | 0.9156 |
| FUS | **0.8771** | 0.8618 | 0.8572 | 0.8287 | 0.8397 | 0.8371 | 0.8572 |
| FXR1 | **0.9964** | 0.9948 | 0.9584 | 0.8986 | 0.9203 | 0.9475 | 0.9584 |
| FXR2 | **0.9712** | 0.9518 | 0.9738 | 0.9192 | 0.9298 | 0.973 | 0.9738 |
| HNRNPC | **0.9831** | 0.9771 | 0.8897 | 0.9203 | 0.9068 | 0.8663 | 0.8897 |
| HUR | **0.9201** | 0.8758 | 0.7157 | 0.8738 | 0.8366 | 0.8029 | 0.7157 |
| IGF2BP1 | 0.9041 | 0.8554 | 0.8363 | 0.848 | **0.968** | 0.8291 | 0.8363 |
| IGF2BP2 | 0.8551 | 0.8426 | 0.8058 | **0.9452** | 0.8277 | 0.8106 | 0.8058 |
| IGF2BP3 | **0.8812** | 0.8229 | 0.7588 | 0.7933 | 0.7947 | 0.7578 | 0.7688 |
| LIN28A | **0.9127** | 0.8751 | 0.8117 | 0.8397 | 0.8499 | 0.8254 | 0.8117 |
| LIN28B | 0.9311 | 0.9014 | 0.9476 | 0.8316 | 0.8374 | 0.9452 | **0.9476** |
| METTL3 | **0.8821** | 0.8649 | 0.879 | 0.8067 | 0.8064 | 0.871 | 0.879 |
| MOV10 | **0.9012** | 0.8674 | 0.8595 | 0.8446 | 0.8096 | 0.8195 | 0.8595 |
| PTB | **0.8713** | 0.8347 | 0.819 | 0.8232 | 0.8319 | 0.8973 | 0.819 |
| PUM2 | **0.9813** | 0.9758 | 0.919 | 0.9414 | 0.9368 | 0.9245 | 0.919 |
| QKI | **0.9911** | 0.9879 | 0.8919 | 0.9619 | 0.9644 | 0.882 | 0.8919 |
| SFRS1 | **0.9821** | 0.9684 | 0.9563 | 0.9412 | 0.936 | 0.9465 | 0.9563 |
| TAF15 | **0.9972** | 0.9945 | 0.9277 | 0.9746 | 0.9635 | 0.9209 | 0.9277 |
| TDP43 | **0.9772** | 0.9336 | 0.9048 | 0.9693 | 0.9717 | 0.8282 | 0.9048 |
| TIA1 | **0.9812** | 0.9666 | 0.8665 | 0.8115 | 0.823 | 0.9089 | 0.8665 |
| TIAL1 | 0.9381 | 0.9249 | 0.852 | **0.983** | 0.9665 | 0.9482 | 0.852 |
| TNRC6 | **0.9851** | 0.9797 | 0.9224 | 0.809 | 0.807 | 0.9504 | 0.9224 |
| U2AF65 | **0.9961** | 0.9306 | 0.9783 | 0.829 | 0.8138 | 0.9524 | 0.9783 |
| WTAP | **0.9831** | 0.9713 | 0.9462 | 0.8124 | 0.8455 | 0.911 | 0.9462 |
| ZC3H7B | **0.8451** | 0.8151 | 0.7891 | 0.8066 | 0.8037 | 0.8006 | 0.7891 |
| **AVG** | **0.9423** | 0.9188 | 0.8728 | 0.8854 | 0.8877 | 0.8758 | 0.8733 |
