# Supplementary material for: iCRBP-LKHA: Large convolutional kernel and hybrid channel-spatial attention for identifying circRNA-RBP interaction sites
Source: PLoS Comput Biol. 2024 Aug 22;20(8):e1012399. doi: 10.1371/journal.pcbi.1012399 (PMC11373821; doi:10.1371/journal.pcbi.1012399)
Supplement: S5 Table — (DOCX) [file pcbi.1012399.s005.docx]

**Supplementary Table 5.** Ablation experiments on 37 circRNA datasets.

|  | iCRBP-LKHA | (w/o) LKCNN | (w/o) CBAM-1D | (w/o) BiGRU | (w/o) LKCNN & CBAM-1D | LKCNN->CNN | CBAM-1D->CBAM |
| --- | --- | --- | --- | --- | --- | --- | --- |
| AGO1 | 0.9431 | 0.756 | 0.7897 | 0.8095 | 0.6038 | 0.8353 | 0.8509 |
| AGO2 | 0.8772 | 0.7007 | 0.7062 | 0.7354 | 0.5362 | 0.7947 | 0.7903 |
| AGO3 | 0.9771 | 0.8575 | 0.8259 | 0.8828 | 0.6291 | 0.8962 | 0.8991 |
| ALKBH5 | 0.9961 | 0.7983 | 0.7802 | 0.9062 | 0.6918 | 0.8958 | 0.9244 |
| AUF1 | 0.9871 | 0.8035 | 0.8166 | 0.8818 | 0.6158 | 0.8802 | 0.9118 |
| C17ORF85 | 0.9912 | 0.807 | 0.8072 | 0.9014 | 0.6304 | 0.908 | 0.9132 |
| C22ORF28 | 0.9291 | 0.8271 | 0.7689 | 0.7982 | 0.5846 | 0.8469 | 0.8568 |
| CAPRIN1 | 0.9271 | 0.804 | 0.7188 | 0.8149 | 0.6203 | 0.8259 | 0.8374 |
| DGCR8 | 0.9542 | 0.8395 | 0.7364 | 0.874 | 0.6311 | 0.8532 | 0.8565 |
| EIF4A3 | 0.8651 | 0.7531 | 0.6644 | 0.7367 | 0.5577 | 0.7626 | 0.7837 |
| EWSR1 | 0.9571 | 0.8027 | 0.7434 | 0.8389 | 0.6391 | 0.8501 | 0.8815 |
| FMRP | 0.9421 | 0.8127 | 0.7263 | 0.7961 | 0.5478 | 0.8566 | 0.8442 |
| FOX2 | 0.9772 | 0.8668 | 0.8181 | 0.8579 | 0.6701 | 0.8802 | 0.8887 |
| FUS | 0.8771 | 0.7012 | 0.7143 | 0.7368 | 0.5124 | 0.7895 | 0.7905 |
| FXR1 | 0.9964 | 0.8645 | 0.8246 | 0.8668 | 0.669 | 0.9095 | 0.9047 |
| FXR2 | 0.9712 | 0.8339 | 0.7937 | 0.8664 | 0.6345 | 0.8906 | 0.8912 |
| HNRNPC | 0.9831 | 0.8497 | 0.7918 | 0.8624 | 0.6235 | 0.891 | 0.8975 |
| HUR | 0.9201 | 0.8119 | 0.7457 | 0.8166 | 0.5622 | 0.8318 | 0.8443 |
| IGF2BP1 | 0.9041 | 0.7496 | 0.7373 | 0.7586 | 0.5097 | 0.8235 | 0.8217 |
| IGF2BP2 | 0.8551 | 0.6678 | 0.696 | 0.7477 | 0.4898 | 0.75 | 0.7656 |
| IGF2BP3 | 0.8812 | 0.6956 | 0.6706 | 0.7677 | 0.5779 | 0.7761 | 0.8036 |
| LIN28A | 0.9127 | 0.731 | 0.7254 | 0.7698 | 0.5475 | 0.8035 | 0.8217 |
| LIN28B | 0.931 | 0.7988 | 0.7343 | 0.8293 | 0.5761 | 0.8276 | 0.852 |
| METTL3 | 0.8821 | 0.7695 | 0.6715 | 0.7331 | 0.5077 | 0.7968 | 0.8111 |
| MOV10 | 0.9012 | 0.749 | 0.7422 | 0.7643 | 0.5318 | 0.8054 | 0.8153 |
| PTB | 0.8713 | 0.7568 | 0.7122 | 0.7245 | 0.4961 | 0.7684 | 0.7913 |
| PUM2 | 0.9813 | 0.814 | 0.8271 | 0.8881 | 0.6397 | 0.8877 | 0.8815 |
| QKI | 0.9911 | 0.8171 | 0.7789 | 0.865 | 0.6425 | 0.8843 | 0.9018 |
| SFRS1 | 0.9821 | 0.8493 | 0.8 | 0.8543 | 0.675 | 0.8978 | 0.8868 |
| TAF15 | 0.9972 | 0.8899 | 0.8048 | 0.9024 | 0.6757 | 0.89 | 0.9078 |
| TDP43 | 0.9772 | 0.87 | 0.7893 | 0.8305 | 0.6019 | 0.8835 | 0.8831 |
| TIA1 | 0.9812 | 0.8044 | 0.8058 | 0.9011 | 0.6047 | 0.8859 | 0.9029 |
| TIAL1 | 0.9381 | 0.7938 | 0.7245 | 0.8 | 0.5633 | 0.8409 | 0.8631 |
| TNRC6 | 0.9851 | 0.7913 | 0.7698 | 0.8559 | 0.6285 | 0.898 | 0.9088 |
| U2AF65 | 0.9961 | 0.8445 | 0.8446 | 0.9019 | 0.6944 | 0.9018 | 0.9256 |
| WTAP | 0.9831 | 0.8651 | 0.7853 | 0.8483 | 0.6332 | 0.8747 | 0.8882 |
| ZC3H7B | 0.8451 | 0.7391 | 0.6347 | 0.7129 | 0.47 | 0.7477 | 0.7647 |
