# Supplementary material for: iCRBP-LKHA: Large convolutional kernel and hybrid channel-spatial attention for identifying circRNA-RBP interaction sites
Source: PLoS Comput Biol. 2024 Aug 22;20(8):e1012399. doi: 10.1371/journal.pcbi.1012399 (PMC11373821; doi:10.1371/journal.pcbi.1012399)
Supplement: S9 Table — Bold data represent the best AUC values of experimental results. (DOCX) [file pcbi.1012399.s009.docx]

| **Dataset37** | **iCRBP-LKHA** | **SVM** | **RF** | **XGBoost** | **LightGBM** | **Rotation Forest** |
| --- | --- | --- | --- | --- | --- | --- |
| AGO1 | **0.9431±0.001** | 0.7907 | 0.7681 | 0.7783 | 0.7469 | 0.7513 |
| AGO2 | **0.8772±0.001** | 0.6768 | 0.6928 | 0.7168 | 0.6781 | 0.6623 |
| AGO3 | **0.9771±0.003** | 0.7463 | 0.7803 | 0.7366 | 0.7393 | 0.8066 |
| ALKBH5 | **0.9961±0.001** | 0.8032 | 0.8795 | 0.858 | 0.8572 | 0.8255 |
| AUF1 | 0.9871±0.001 | 0.7963 | 0.8234 | 0.8767 | 0.8274 | 0.8302 |
| C17ORF85 | **0.9912±0.001** | 0.8215 | 0.8449 | 0.7971 | 0.8657 | 0.8126 |
| C22ORF28 | **0.9291±0.003** | 0.7491 | 0.7498 | 0.7817 | 0.7899 | 0.813 |
| CAPRIN1 | **0.9271±0.001** | 0.7261 | 0.6958 | 0.7219 | 0.7386 | 0.7099 |
| DGCR8 | **0.9542±0.002** | 0.7928 | 0.7767 | 0.7956 | 0.7496 | 0.8011 |
| EIF4A3 | **0.8651±0.005** | 0.6882 | 0.7091 | 0.6476 | 0.6831 | 0.6434 |
| EWSR1 | **0.9571±0.003** | 0.8151 | 0.8013 | 0.7875 | 0.8014 | 0.8019 |
| FMRP | **0.9421±0.001** | 0.7206 | 0.7433 | 0.7227 | 0.7522 | 0.7323 |
| FOX2 | **0.9772±0.003** | 0.8028 | 0.7848 | 0.8223 | 0.7855 | 0.8545 |
| FUS | **0.8771±0.001** | 0.7691 | 0.7393 | 0.7229 | 0.727 | 0.7588 |
| FXR1 | **0.9964±0.003** | 0.8795 | 0.8169 | 0.8712 | 0.8793 | 0.8926 |
| FXR2 | **0.9712±0.002** | 0.7843 | 0.8336 | 0.8205 | 0.783 | 0.7932 |
| HNRNPC | **0.9831±0.003** | 0.7858 | 0.809 | 0.7833 | 0.8759 | 0.8351 |
| HUR | **0.9201±0.013** | 0.6965 | 0.7585 | 0.7723 | 0.747 | 0.7392 |
| IGF2BP1 | **0.9041±0.003** | 0.705 | 0.7024 | 0.7171 | 0.7435 | 0.6797 |
| IGF2BP2 | **0.8551±0.003** | 0.6698 | 0.7182 | 0.6713 | 0.6929 | 0.68 |
| IGF2BP3 | **0.8812±0.001** | 0.6655 | 0.6971 | 0.6552 | 0.677 | 0.7007 |
| LIN28A | **0.9127±0.004** | 0.7325 | 0.7161 | 0.6939 | 0.6997 | 0.7236 |
| LIN28B | **0.9311±0.012** | 0.7484 | 0.7757 | 0.7626 | 0.7878 | 0.7498 |
| METTL3 | **0.8821±0.011** | 0.7557 | 0.6822 | 0.7381 | 0.7473 | 0.7093 |
| MOV10 | **0.9012±0.022** | 0.7032 | 0.6953 | 0.6923 | 0.717 | 0.733 |
| PTB | **0.8713±0.015** | 0.6722 | 0.735 | 0.7136 | 0.7209 | 0.6874 |
| PUM2 | **0.9813±0.012** | 0.7786 | 0.8498 | 0.7782 | 0.8221 | 0.83 |
| QKI | **0.9911±0.006** | 0.8553 | 0.8606 | 0.7801 | 0.8231 | 0.8325 |
| SFRS1 | **0.9821±0.006** | 0.8181 | 0.7741 | 0.8634 | 0.7793 | 0.8647 |
| TAF15 | **0.9972±0.002** | 0.8339 | 0.831 | 0.8503 | 0.8353 | 0.8828 |
| TDP43 | **0.9772±0.003** | 0.7506 | 0.7685 | 0.8265 | 0.7646 | 0.805 |
| TIA1 | **0.9812±0.009** | 0.8383 | 0.8187 | 0.8549 | 0.779 | 0.8065 |
| TIAL1 | **0.9381±0.001** | 0.7405 | 0.7441 | 0.8035 | 0.7623 | 0.7687 |
| TNRC6 | **0.9851±0.002** | 0.801 | 0.8253 | 0.7959 | 0.8361 | 0.7766 |
| U2AF65 | **0.9961±0.002** | 0.7593 | 0.7756 | 0.8319 | 0.8208 | 0.763 |
| WTAP | **0.9831±0.004** | 0.8184 | 0.8141 | 0.8341 | 0.8215 | 0.7861 |
| ZC3H7B | **0.8451±0.008** | 0.6571 | 0.7052 | 0.6867 | 0.6841 | 0.6685 |
| **AVG** | **0.9424±0.003** | 0.7608 | 0.7702 | 0.7720 | 0.7714 | 0.7706 |

**Supplementary Table 9.** Comparison of AUC between iCRBP-LKHA and five shallow learning algorithms on 37 circRNAs stringent datasets. Bold data represent the best AUC values of experimental results.
