# Supplementary material for: iCRBP-LKHA: Large convolutional kernel and hybrid channel-spatial attention for identifying circRNA-RBP interaction sites
Source: PLoS Comput Biol. 2024 Aug 22;20(8):e1012399. doi: 10.1371/journal.pcbi.1012399 (PMC11373821; doi:10.1371/journal.pcbi.1012399)
Supplement: S10 Table — Bold data represent the best ACC values of experimental results. (DOCX) [file pcbi.1012399.s010.docx]

| **Dataset37** | **iCRBP-LKHA** | **SVM** | **RF** | **XGBoost** | **LightGBM** | **Rotation Forest** |
| --- | --- | --- | --- | --- | --- | --- |
| AGO1 | **0.9295±0.004** | 0.8653 | 0.8075 | 0.7517 | 0.8358 | 0.8612 |
| AGO2 | **0.8646±0.002** | 0.7122 | 0.6996 | 0.7018 | 0.6863 | 0.7212 |
| AGO3 | **0.963±0.003** | 0.7582 | 0.8031 | 0.7922 | 0.8554 | 0.7684 |
| ALKBH5 | **0.9817±0.004** | 0.521 | 0.5206 | 0.5703 | 0.5338 | 0.6027 |
| AUF1 | 0.9729±0.002 | 0.8819 | 0.9027 | 0.9226 | **0.9882** | 0.9833 |
| C17ORF85 | **0.9769±0.003** | 0.6942 | 0.6878 | 0.689 | 0.6466 | 0.7233 |
| C22ORF28 | **0.9157±0.004** | 0.6952 | 0.7736 | 0.7289 | 0.6979 | 0.7518 |
| CAPRIN1 | **0.9137±0.003** | 0.7334 | 0.7039 | 0.7555 | 0.7751 | 0.6966 |
| DGCR8 | **0.9404±0.001** | 0.7333 | 0.8432 | 0.8112 | 0.8839 | 0.8247 |
| EIF4A3 | **0.8526±0.004** | 0.7655 | 0.6811 | 0.7576 | 0.6657 | 0.7533 |
| EWSR1 | 0.9433±0.003 | 0.9361 | 0.8573 | 0.8489 | 0.8145 | **0.9507** |
| FMRP | **0.9285±0.004** | 0.7487 | 0.8472 | 0.8582 | 0.854 | 0.8616 |
| FOX2 | **0.9631±0.004** | 0.5275 | 0.5765 | 0.5696 | 0.6187 | 0.6048 |
| FUS | **0.8645±0.004** | 0.6937 | 0.8067 | 0.7426 | 0.7286 | 0.7435 |
| FXR1 | 0.982±0.004 | 0.9307 | 0.8832 | **0.9996** | 0.9253 | 0.9593 |
| FXR2 | **0.9572±0.001** | 0.9062 | 0.9355 | 0.7975 | 0.8124 | 0.8216 |
| HNRNPC | 0.9689±0.003 | **0.9734** | 0.9649 | 0.9613 | 0.8663 | 0.9309 |
| HUR | **0.9068±0.001** | 0.7279 | 0.6946 | 0.7998 | 0.8105 | 0.8347 |
| IGF2BP1 | **0.8911±0.004** | 0.7115 | 0.7886 | 0.7481 | 0.8103 | 0.8171 |
| IGF2BP2 | **0.8428±0.002** | 0.7866 | 0.7543 | 0.7307 | 0.7779 | 0.6593 |
| IGF2BP3 | **0.8685±0.003** | 0.6367 | 0.7206 | 0.7238 | 0.6778 | 0.7427 |
| LIN28A | **0.8995±0.001** | 0.7858 | 0.6983 | 0.7868 | 0.7534 | 0.7926 |
| LIN28B | **0.9177±0.003** | 0.8121 | 0.8172 | 0.8237 | 0.745 | 0.8379 |
| METTL3 | **0.8694±0.004** | 0.7992 | 0.7755 | 0.7694 | 0.7275 | 0.7374 |
| MOV10 | **0.8882±0.001** | 0.6943 | 0.7294 | 0.8071 | 0.7935 | 0.7412 |
| PTB | **0.8587±0.004** | 0.7229 | 0.7632 | 0.7808 | 0.779 | 0.6625 |
| PUM2 | **0.9672±0.004** | 0.8559 | 0.8199 | 0.9473 | 0.8422 | 0.8774 |
| QKI | **0.9768±0.003** | 0.849 | 0.7852 | 0.7868 | 0.8991 | 0.8773 |
| SFRS1 | **0.9679±0.003** | 0.9023 | 0.9555 | 0.9425 | 0.9671 | 0.9617 |
| TAF15 | **0.9828±0.002** | 0.8688 | 0.9615 | 0.9191 | 0.9121 | 0.9734 |
| TDP43 | **0.9631±0.003** | 0.8011 | 0.9285 | 0.9339 | 0.8902 | 0.883 |
| TIA1 | **0.9671±0.003** | 0.7944 | 0.8373 | 0.8153 | 0.8707 | 0.8859 |
| TIAL1 | **0.9246±0.002** | 0.8119 | 0.9092 | 0.8565 | 0.865 | 0.7637 |
| TNRC6 | **0.9709±0.003** | 0.6935 | 0.6142 | 0.6969 | 0.6127 | 0.5839 |
| U2AF65 | **0.9817±0.002** | 0.9142 | 0.8317 | 0.8691 | 0.8418 | 0.8507 |
| WTAP | **0.9689±0.002** | 0.7444 | 0.7116 | 0.7602 | 0.6612 | 0.6389 |
| ZC3H7B | **0.8329±0.002** | 0.6453 | 0.6297 | 0.6167 | 0.6537 | 0.7416 |
| **AVG** | 0.9288±0.003 | 0.7739 | 0.7843 | 0.7939 | 0.7859 | 0.7952 |

**Supplementary Table 10.** Comparison of ACC between iCRBP-LKHA and five shallow learning algorithms on 37 circRNAs stringent datasets. Bold data represent the best ACC values of experimental results.
