# Supplementary material for: iCRBP-LKHA: Large convolutional kernel and hybrid channel-spatial attention for identifying circRNA-RBP interaction sites
Source: PLoS Comput Biol. 2024 Aug 22;20(8):e1012399. doi: 10.1371/journal.pcbi.1012399 (PMC11373821; doi:10.1371/journal.pcbi.1012399)
Supplement: S11 Table — Bold data represent the best F1 values of experimental results. (DOCX) [file pcbi.1012399.s011.docx]

| **Dataset37** | **iCRBP-LKHA** | **SVM** | **RF** | **XGBoost** | **LightGBM** | **Rotation Forest** |
| --- | --- | --- | --- | --- | --- | --- |
| AGO1 | **0.8915±0.002** | 0.736 | 0.7309 | 0.7631 | 0.7781 | 0.7407 |
| AGO2 | **0.8292±0.002** | 0.6016 | 0.6599 | 0.6278 | 0.6358 | 0.6396 |
| AGO3 | **0.9236±0.002** | 0.7855 | 0.7448 | 0.7252 | 0.7831 | 0.7689 |
| ALKBH5 | **0.9416±0.003** | 0.5055 | 0.4807 | 0.5249 | 0.5015 | 0.5379 |
| AUF1 | **0.9331±0.004** | 0.889 | 0.8571 | 0.8205 | 0.8057 | 0.8435 |
| C17ORF85 | **0.937±0.004** | 0.6759 | 0.6763 | 0.601 | 0.6056 | 0.6651 |
| C22ORF28 | **0.8783±0.002** | 0.7199 | 0.7403 | 0.7121 | 0.7269 | 0.7245 |
| CAPRIN1 | **0.8764±0.001** | 0.6879 | 0.6705 | 0.7119 | 0.651 | 0.6519 |
| DGCR8 | **0.902±0.002** | 0.697 | 0.7257 | 0.7409 | 0.732 | 0.7686 |
| EIF4A3 | **0.8178±0.004** | 0.6008 | 0.6621 | 0.6003 | 0.6252 | 0.6318 |
| EWSR1 | **0.9047±0.002** | 0.8183 | 0.8167 | 0.8574 | 0.7868 | 0.7897 |
| FMRP | **0.8906±0.004** | 0.7614 | 0.7166 | 0.6944 | 0.7705 | 0.7283 |
| FOX2 | **0.9237±0.003** | 0.5534 | 0.5481 | 0.5137 | 0.5164 | 0.4905 |
| FUS | **0.8291±0.002** | 0.7239 | 0.6807 | 0.6795 | 0.733 | 0.6547 |
| FXR1 | **0.9419±0.001** | 0.7951 | 0.8464 | 0.8695 | 0.8869 | 0.8459 |
| FXR2 | **0.9181±0.001** | 0.8145 | 0.7873 | 0.8421 | 0.7999 | 0.8085 |
| HNRNPC | **0.9293±0.004** | 0.8809 | 0.8732 | 0.8186 | 0.8626 | 0.8482 |
| HUR | **0.8698±0.001** | 0.6721 | 0.7351 | 0.6964 | 0.7538 | 0.7507 |
| IGF2BP1 | **0.8546±0.003** | 0.6907 | 0.6389 | 0.6788 | 0.6777 | 0.6439 |
| IGF2BP2 | **0.8083±0.001** | 0.6818 | 0.6392 | 0.6201 | 0.6351 | 0.6981 |
| IGF2BP3 | **0.833±0.004** | 0.6374 | 0.6032 | 0.5959 | 0.6285 | 0.5846 |
| LIN28A | **0.8628±0.004** | 0.6694 | 0.6393 | 0.6387 | 0.6552 | 0.6451 |
| LIN28B | **0.8802±0.002** | 0.7024 | 0.7 | 0.7106 | 0.7357 | 0.6848 |
| METTL3 | **0.8338±0.002** | 0.6313 | 0.6351 | 0.6331 | 0.6412 | 0.6537 |
| MOV10 | **0.8519±0.001** | 0.6889 | 0.7195 | 0.6789 | 0.6714 | 0.681 |
| PTB | **0.8236±0.003** | 0.6388 | 0.6897 | 0.6554 | 0.6345 | 0.7088 |
| PUM2 | **0.9276±0.001** | 0.8601 | 0.8605 | 0.8244 | 0.8646 | 0.7866 |
| QKI | **0.9369±0.004** | 0.7365 | 0.7839 | 0.8246 | 0.8262 | 0.7955 |
| SFRS1 | **0.9284±0.002** | 0.787 | 0.7813 | 0.8541 | 0.826 | 0.8448 |
| TAF15 | **0.9426±0.001** | 0.8438 | 0.8291 | 0.8772 | 0.841 | 0.8024 |
| TDP43 | **0.9237±0.002** | 0.755 | 0.8213 | 0.8356 | 0.8339 | 0.7976 |
| TIA1 | **0.9275±0.003** | 0.8164 | 0.806 | 0.859 | 0.7961 | 0.8101 |
| TIAL1 | **0.8868±0.003** | 0.7823 | 0.7725 | 0.7858 | 0.7133 | 0.7563 |
| TNRC6 | **0.9312±0.001** | 0.5563 | 0.5837 | 0.6167 | 0.6146 | 0.5632 |
| U2AF65 | **0.9416±0.001** | 0.7997 | 0.8462 | 0.7644 | 0.8459 | 0.7511 |
| WTAP | **0.9293±0.002** | 0.6499 | 0.6888 | 0.6518 | 0.6217 | 0.6528 |
| ZC3H7B | **0.7989±0.003** | 0.6323 | 0.6332 | 0.611 | 0.6531 | 0.6192 |
| **AVG** | **0.8908±0.003** | 0.7156 | 0.7196 | 0.7166 | 0.7208 | 0.7127 |

**Supplementary Table 11.** Comparison of F1 between iCRBP-LKHA and five shallow learning algorithms on 37 circRNAs stringent datasets. Bold data represent the best F1 values of experimental results.
