# Supplementary material for: iCRBP-LKHA: Large convolutional kernel and hybrid channel-spatial attention for identifying circRNA-RBP interaction sites
Source: PLoS Comput Biol. 2024 Aug 22;20(8):e1012399. doi: 10.1371/journal.pcbi.1012399 (PMC11373821; doi:10.1371/journal.pcbi.1012399)
Supplement: S12 Table — Bold data represent the best MCC values of experimental results. (DOCX) [file pcbi.1012399.s012.docx]

| **Dataset37** | **iCRBP-LKHA** | **SVM** | **RF** | **XGBoost** | **LightGBM** | **Rotation Forest** |
| --- | --- | --- | --- | --- | --- | --- |
| AGO1 | **0.7615±0.004** | 0.6068 | 0.6563 | 0.5963 | 0.7081 | 0.645 |
| AGO2 | **0.7083±0.001** | 0.6018 | 0.5512 | 0.5767 | 0.5294 | 0.5196 |
| AGO3 | **0.789±0.004** | 0.62 | 0.6718 | 0.6697 | 0.6978 | 0.6222 |
| ALKBH5 | **0.8043±0.002** | 0.4897 | 0.4101 | 0.4899 | 0.4851 | 0.4814 |
| AUF1 | **0.7971±0.002** | 0.747 | 0.7481 | 0.721 | 0.7004 | 0.7496 |
| C17ORF85 | **0.8004±0.002** | 0.5395 | 0.6057 | 0.6228 | 0.6124 | 0.6028 |
| C22ORF28 | **0.7502±0.002** | 0.5589 | 0.6425 | 0.6446 | 0.5981 | 0.5543 |
| CAPRIN1 | **0.7486±0.001** | 0.5933 | 0.5837 | 0.5785 | 0.5876 | 0.5333 |
| DGCR8 | **0.7705±0.003** | 0.6895 | 0.593 | 0.6757 | 0.6766 | 0.6319 |
| EIF4A3 | **0.6985±0.004** | 0.6092 | 0.5473 | 0.5516 | 0.5993 | 0.5278 |
| EWSR1 | **0.7728±0.001** | 0.7331 | 0.6373 | 0.6607 | 0.668 | 0.6883 |
| FMRP | **0.7607±0.004** | 0.6337 | 0.6175 | 0.6619 | 0.5887 | 0.6404 |
| FOX2 | **0.7891±0.002** | 0.5118 | 0.479 | 0.4603 | 0.4604 | 0.4847 |
| FUS | **0.7082±0.003** | 0.5562 | 0.6278 | 0.5824 | 0.6082 | 0.5738 |
| FXR1 | **0.8046±0.003** | 0.8035 | 0.7619 | 0.7311 | 0.7615 | 0.6618 |
| FXR2 | **0.7842±0.002** | 0.6916 | 0.747 | 0.6393 | 0.6766 | 0.7234 |
| HNRNPC | **0.7938±0.002** | 0.7452 | 0.7903 | 0.6717 | 0.678 | 0.744 |
| HUR | **0.743±0.002** | 0.5734 | 0.6639 | 0.6855 | 0.654 | 0.5728 |
| IGF2BP1 | **0.73±0.002** | 0.5955 | 0.5251 | 0.5827 | 0.6251 | 0.5482 |
| IGF2BP2 | **0.6905±0.002** | 0.6134 | 0.5859 | 0.6181 | 0.5931 | 0.5201 |
| IGF2BP3 | **0.7115±0.002** | 0.5002 | 0.598 | 0.5203 | 0.5265 | 0.5077 |
| LIN28A | **0.737±0.002** | 0.5835 | 0.5917 | 0.5914 | 0.5834 | 0.5381 |
| LIN28B | **0.7518±0.002** | 0.7086 | 0.6848 | 0.6409 | 0.6062 | 0.5929 |
| METTL3 | **0.7123±0.002** | 0.6131 | 0.6596 | 0.5648 | 0.6013 | 0.5455 |
| MOV10 | **0.7277±0.003** | 0.6137 | 0.537 | 0.6126 | 0.531 | 0.5517 |
| PTB | **0.7035±0.004** | 0.5986 | 0.6251 | 0.5874 | 0.5749 | 0.538 |
| PUM2 | **0.7924±0.002** | 0.7012 | 0.7362 | 0.7607 | 0.6669 | 0.7629 |
| QKI | **0.8003±0.002** | 0.7316 | 0.6451 | 0.7018 | 0.733 | 0.6063 |
| SFRS1 | 0.793±0.001 | 0.6765 | 0.7031 | 0.7554 | 0.7699 | **0.8018** |
| TAF15 | **0.8052±0.002** | 0.7657 | 0.6853 | 0.7739 | 0.7498 | 0.6775 |
| TDP43 | **0.7891±0.001** | 0.625 | 0.693 | 0.7012 | 0.6793 | 0.725 |
| TIA1 | **0.7923±0.002** | 0.7059 | 0.7404 | 0.6641 | 0.6809 | 0.6715 |
| TIAL1 | **0.7575±0.001** | 0.6772 | 0.6879 | 0.6328 | 0.6824 | 0.6997 |
| TNRC6 | **0.7954±0.001** | 0.5272 | 0.5707 | 0.5745 | 0.5006 | 0.5533 |
| U2AF65 | **0.8043±0.001** | 0.6807 | 0.6235 | 0.7202 | 0.712 | 0.687 |
| WTAP | **0.7938±0.002** | 0.5295 | 0.5994 | 0.5951 | 0.6036 | 0.5594 |
| ZC3H7B | **0.6824±0.001** | 0.5816 | 0.6025 | 0.5997 | 0.5565 | 0.5068 |
| **AVG** | 0.7609±0.003 | 0.6306 | 0.6332 | 0.6329 | 0.6288 | 0.6095 |

**Supplementary Table 12.** Comparison of MCC between iCRBP-LKHA and five shallow learning algorithms on 37 circRNAs stringent datasets. Bold data represent the best MCC values of experimental results.
