# Supplementary material for: iCRBP-LKHA: Large convolutional kernel and hybrid channel-spatial attention for identifying circRNA-RBP interaction sites
Source: PLoS Comput Biol. 2024 Aug 22;20(8):e1012399. doi: 10.1371/journal.pcbi.1012399 (PMC11373821; doi:10.1371/journal.pcbi.1012399)
Supplement: S13 Table — Bold data represent the best ACC values of experimental results. (DOCX) [file pcbi.1012399.s013.docx]

| **Dataset37** | **iCRBP-LKHA** | **ASCRB** | **iCircRBP-DHN** | **PASSION** | **CRIP** | **CSCRites** | \| **CircSLNN** \| \| --- \| | **CRBPDL** |
| --- | --- | --- | --- | --- | --- | --- | --- | --- | --- |
| AGO1 | **0.9295±0.004** | 0.9226 | 0.8596±0.002 | 0.877±0.002 | 0.882±0.001 | 0.815±0.001 | 0.808±0.003 | 0.9047±0.004 |
| AGO2 | **0.8646±0.002** | 0.8232 | 0.7637±0.003 | 0.782±0.001 | 0.774±0.001 | 0.734±0.003 | 0.69±0.003 | 0.8028±0.001 |
| AGO3 | **0.963±0.003** | 0.9434 | 0.8782±0.002 | 0.874±0.001 | 0.873±0.003 | 0.796±0.001 | 0.819±0.003 | 0.9274±0.001 |
| ALKBH5 | **0.9817±0.004** | 0.948 | 0.9354±0.001 | 0.736±0.004 | 0.693±0.003 | 0.764±0.004 | 0.562±0.001 | 0.974±0.003 |
| AUF1 | **0.9729±0.002** | 0.951 | 0.9416±0.002 | 0.938±0.001 | 0.954±0.004 | 0.903±0.004 | 0.937±0.002 | 0.9372±0.003 |
| C17ORF85 | **0.9769±0.003** | 0.9504 | 0.947±0.002 | 0.818±0.001 | 0.781±0.001 | 0.797±0.003 | 0.695±0.004 | 0.9566±0.004 |
| C22ORF28 | **0.9157±0.004** | 0.9116 | 0.8935±0.001 | 0.869±0.004 | 0.835±0.001 | 0.837±0.003 | 0.772±0.004 | 0.8702±0.002 |
| CAPRIN1 | **0.9137±0.003** | 0.8921 | 0.8363±0.003 | 0.833±0.001 | 0.804±0.004 | 0.798±0.001 | 0.728±0.004 | 0.8446±0.001 |
| DGCR8 | **0.9404±0.001** | 0.9098 | 0.863±0.004 | 0.898±0.004 | 0.89±0.002 | 0.83±0.001 | 0.805±0.003 | 0.9032±0.004 |
| EIF4A3 | **0.8526±0.004** | 0.8228 | 0.7816±0.001 | 0.795±0.002 | 0.791±0.003 | 0.786±0.001 | 0.7±0.002 | 0.831±0.002 |
| EWSR1 | **0.9433±0.003** | 0.9162 | 0.9086±0.003 | 0.908±0.004 | 0.899±0.002 | 0.859±0.003 | 0.872±0.004 | 0.9062±0.004 |
| FMRP | **0.9285±0.004** | 0.9083 | 0.8602±0.001 | 0.879±0.002 | 0.865±0.002 | 0.87±0.004 | 0.792±0.002 | 0.8776±0.001 |
| FOX2 | **0.9631±0.004** | 0.9465 | 0.9145±0.004 | 0.813±0.003 | 0.79±0.003 | 0.733±0.002 | 0.585±0.001 | 0.9334±0.001 |
| FUS | 0.8645±0.004 | **0.8793** | 0.8285±0.002 | 0.816±0.003 | 0.831±0.001 | 0.782±0.004 | 0.753±0.001 | 0.8403±0.002 |
| FXR1 | **0.982±0.004** | 0.9546 | 0.9643±0.004 | 0.934±0.002 | 0.914±0.003 | 0.847±0.003 | 0.909±0.004 | 0.9633±0.002 |
| FXR2 | **0.9572±0.001** | 0.9406 | 0.895±0.001 | 0.898±0.001 | 0.9±0.003 | 0.826±0.001 | 0.86±0.002 | 0.9131±0.002 |
| HNRNPC | **0.9689±0.003** | 0.9516 | 0.9316±0.003 | 0.928±0.002 | 0.935±0.002 | 0.924±0.001 | 0.934±0.001 | 0.9319±0.001 |
| HUR | **0.9068±0.001** | 0.8727 | 0.8244±0.003 | 0.85±0.003 | 0.848±0.004 | 0.831±0.002 | 0.762±0.004 | 0.8366±0.001 |
| IGF2BP1 | **0.8911±0.004** | 0.8832 | 0.8227±0.002 | 0.816±0.001 | 0.822±0.002 | 0.799±0.002 | 0.743±0.003 | 0.8357±0.001 |
| IGF2BP2 | 0.8428±0.002 | **0.9002** | 0.8009±0.003 | 0.789±0.002 | 0.804±0.002 | 0.727±0.003 | 0.721±0.003 | 0.8141±0.004 |
| IGF2BP3 | 0.8685±0.003 | **0.8731** | 0.781±0.004 | 0.807±0.002 | 0.789±0.001 | 0.737±0.004 | 0.683±0.001 | 0.7873±0.003 |
| LIN28A | **0.8995±0.001** | 0.8579 | 0.8331±0.001 | 0.843±0.001 | 0.844±0.003 | 0.804±0.001 | 0.745±0.002 | 0.8488±0.002 |
| LIN28B | **0.9177±0.003** | 0.8359 | 0.8578±0.004 | 0.846±0.004 | 0.843±0.003 | 0.741±0.003 | 0.788±0.003 | 0.8794±0.003 |
| METTL3 | 0.8694±0.004 | **0.8786** | 0.8332±0.004 | 0.843±0.004 | 0.834±0.004 | 0.786±0.003 | 0.734±0.003 | 0.8439±0.002 |
| MOV10 | **0.8882±0.001** | 0.8626 | 0.8156±0.003 | 0.811±0.004 | 0.816±0.003 | 0.751±0.002 | 0.758±0.002 | 0.8299±0.004 |
| PTB | **0.8587±0.004** | 0.8264 | 0.7925±0.002 | 0.807±0.001 | 0.789±0.001 | 0.668±0.002 | 0.712±0.001 | 0.8151±0.002 |
| PUM2 | **0.9672±0.004** | 0.9505 | 0.9249±0.001 | 0.932±0.002 | 0.926±0.002 | 0.902±0.004 | 0.899±0.002 | 0.9349±0.004 |
| QKI | **0.9768±0.003** | 0.9415 | 0.9288±0.002 | 0.891±0.004 | 0.885±0.003 | 0.833±0.002 | 0.832±0.003 | 0.9624±0.003 |
| SFRS1 | **0.9679±0.003** | 0.9484 | 0.9439±0.001 | 0.94±0.003 | 0.94±0.002 | 0.916±0.002 | 0.898±0.004 | 0.9213±0.001 |
| TAF15 | **0.9828±0.002** | 0.9669 | 0.9692±0.002 | 0.926±0.001 | 0.937±0.001 | 0.894±0.001 | 0.929±0.003 | 0.9472±0.004 |
| TDP43 | **0.9631±0.003** | 0.924 | 0.9013±0.003 | 0.908±0.002 | 0.905±0.003 | 0.892±0.003 | 0.862±0.003 | 0.9021±0.001 |
| TIA1 | **0.9671±0.003** | 0.9431 | 0.9167±0.004 | 0.908±0.003 | 0.91±0.001 | 0.889±0.002 | 0.868±0.002 | 0.9238±0.004 |
| TIAL1 | **0.9246±0.002** | 0.9133 | 0.8744±0.003 | 0.872±0.004 | 0.877±0.002 | 0.879±0.002 | 0.846±0.001 | 0.8893±0.002 |
| TNRC6 | **0.9709±0.003** | 0.9351 | 0.9378±0.002 | 0.756±0.003 | 0.707±0.003 | 0.713±0.001 | 0.642±0.004 | 0.9393±0.002 |
| U2AF65 | **0.9817±0.002** | 0.9003 | 0.9011±0.004 | 0.888±0.004 | 0.892±0.003 | 0.872±0.004 | 0.862±0.003 | 0.8951±0.004 |
| WTAP | **0.9689±0.002** | 0.9392 | 0.9366±0.001 | 0.768±0.002 | 0.772±0.004 | 0.79±0.002 | 0.697±0.002 | 0.9442±0.002 |
| ZC3H7B | **0.8329±0.002** | 0.8302 | 0.7733±0.004 | 0.766±0.004 | 0.766±0.001 | 0.764±0.004 | 0.682±0.003 | 0.7816±0.001 |
| **AVG** | **0.9288±0.003** | 0.9069±0.044 | 0.8749±0.006 | 0.8531±0.007 | 0.8464±0.007 | 0.8132±0.006 | 0.7806±0.01 | 0.8878±0.057 |

**Supplementary Table 13.** Comparison of ACC of different methods on 37 circRNA datasets. Bold data represent the best ACC values of experimental results.
