# Supplementary material for: iCRBP-LKHA: Large convolutional kernel and hybrid channel-spatial attention for identifying circRNA-RBP interaction sites
Source: PLoS Comput Biol. 2024 Aug 22;20(8):e1012399. doi: 10.1371/journal.pcbi.1012399 (PMC11373821; doi:10.1371/journal.pcbi.1012399)
Supplement: S14 Table — Bold data represent the best F1 values of experimental results. (DOCX) [file pcbi.1012399.s014.docx]

| **Dataset37** | **iCRBP-LKHA** | **ASCRB** | **iCircRBP-DHN** | **PASSION** | **CRIP** | **CSCRites** | \| **CircSLNN** \| \| --- \| | **CRBPDL** |
| --- | --- | --- | --- | --- | --- | --- | --- | --- | --- |
| AGO1 | **0.8915±0.002** | 0.8677 | 0.8151±0.004 | 0.851±0.003 | 0.821±0.003 | 0.776±0.004 | 0.789±0.003 | 0.8321 |
| AGO2 | **0.8292±0.002** | 0.7749 | 0.7244±0.004 | 0.75±0.004 | 0.748±0.002 | 0.699±0.001 | 0.65±0.001 | 0.7585 |
| AGO3 | **0.9236±0.002** | 0.9091 | 0.8485±0.001 | 0.826±0.004 | 0.822±0.003 | 0.751±0.001 | 0.777±0.003 | 0.8541 |
| ALKBH5 | **0.9416±0.003** | 0.903 | 0.9186±0.001 | 0.702±0.003 | 0.667±0.001 | 0.738±0.001 | 0.531±0.003 | 0.935 |
| AUF1 | **0.9331±0.004** | 0.9064 | 0.903±0.004 | 0.914±0.004 | 0.902±0.004 | 0.88±0.004 | 0.881±0.001 | 0.915 |
| C17ORF85 | **0.937±0.004** | 0.8978 | 0.9228±0.004 | 0.787±0.002 | 0.751±0.004 | 0.742±0.002 | 0.661±0.003 | 0.9097 |
| C22ORF28 | **0.8783±0.002** | 0.865 | 0.8509±0.003 | 0.819±0.001 | 0.821±0.004 | 0.799±0.001 | 0.721±0.004 | 0.8342 |
| CAPRIN1 | **0.8764±0.001** | 0.8364 | 0.8043±0.001 | 0.806±0.004 | 0.776±0.003 | 0.751±0.003 | 0.696±0.002 | 0.8057 |
| DGCR8 | **0.902±0.002** | 0.8698 | 0.8215±0.003 | 0.837±0.001 | 0.853±0.004 | 0.79±0.003 | 0.764±0.001 | 0.8334 |
| EIF4A3 | **0.8178±0.004** | 0.8056 | 0.724±0.001 | 0.745±0.002 | 0.745±0.004 | 0.765±0.001 | 0.657±0.002 | 0.7777 |
| EWSR1 | **0.9047±0.002** | 0.8813 | 0.8675±0.002 | 0.874±0.004 | 0.857±0.002 | 0.827±0.004 | 0.846±0.002 | 0.876 |
| FMRP | **0.8906±0.004** | 0.8729 | 0.8189±0.002 | 0.828±0.004 | 0.814±0.003 | 0.818±0.002 | 0.752±0.002 | 0.839 |
| FOX2 | **0.9237±0.003** | 0.8939 | 0.882±0.001 | 0.778±0.004 | 0.738±0.004 | 0.698±0.003 | 0.543±0.003 | 0.8769 |
| FUS | 0.8291±0.002 | **0.8421** | 0.7699±0.003 | 0.79±0.001 | 0.789±0.002 | 0.72±0.004 | 0.72±0.003 | 0.7761 |
| FXR1 | **0.9419±0.001** | 0.9193 | 0.9233±0.002 | 0.872±0.004 | 0.861±0.003 | 0.811±0.003 | 0.866±0.001 | 0.9224 |
| FXR2 | **0.9181±0.001** | 0.8982 | 0.8718±0.002 | 0.856±0.001 | 0.866±0.002 | 0.795±0.003 | 0.834±0.002 | 0.8903 |
| HNRNPC | **0.9293±0.004** | 0.9165 | 0.9133±0.003 | 0.89±0.002 | 0.898±0.003 | 0.901±0.003 | 0.881±0.002 | 0.8972 |
| HUR | **0.8698±0.001** | 0.831 | 0.8098±0.004 | 0.8±0.004 | 0.794±0.004 | 0.776±0.004 | 0.738±0.002 | 0.8205 |
| IGF2BP1 | **0.8546±0.003** | 0.8306 | 0.773±0.002 | 0.762±0.001 | 0.772±0.002 | 0.766±0.001 | 0.7±0.001 | 0.7718 |
| IGF2BP2 | 0.8083±0.001 | **0.8595** | 0.7519±0.001 | 0.762±0.003 | 0.768±0.001 | 0.697±0.003 | 0.687±0.001 | 0.7896 |
| IGF2BP3 | 0.833±0.004 | **0.8377** | 0.7449±0.002 | 0.761±0.002 | 0.743±0.001 | 0.699±0.004 | 0.64±0.003 | 0.7494 |
| LIN28A | **0.8628±0.004** | 0.8115 | 0.7998±0.001 | 0.79±0.002 | 0.812±0.004 | 0.77±0.001 | 0.7±0.002 | 0.8138 |
| LIN28B | **0.8802±0.002** | 0.8005 | 0.8215±0.002 | 0.833±0.001 | 0.805±0.003 | 0.69±0.001 | 0.754±0.002 | 0.8164 |
| METTL3 | **0.8338±0.002** | 0.8227 | 0.7946±0.004 | 0.808±0.001 | 0.785±0.003 | 0.742±0.002 | 0.695±0.001 | 0.7911 |
| MOV10 | **0.8519±0.001** | 0.818 | 0.7736±0.002 | 0.762±0.001 | 0.775±0.004 | 0.719±0.002 | 0.719±0.004 | 0.8117 |
| PTB | **0.8236±0.003** | 0.8007 | 0.7545±0.001 | 0.756±0.004 | 0.748±0.004 | 0.646±0.002 | 0.69±0.001 | 0.7814 |
| PUM2 | **0.9276±0.001** | 0.9002 | 0.9081±0.002 | 0.885±0.002 | 0.86±0.003 | 0.853±0.004 | 0.846±0.004 | 0.9091 |
| QKI | **0.9369±0.004** | 0.9095 | 0.8845±0.003 | 0.857±0.001 | 0.841±0.002 | 0.794±0.004 | 0.809±0.003 | 0.9146 |
| SFRS1 | **0.9284±0.002** | 0.9045 | 0.8678±0.001 | 0.878±0.002 | 0.888±0.001 | 0.903±0.002 | 0.857±0.001 | 0.8841 |
| TAF15 | **0.9426±0.001** | 0.9274 | 0.9185±0.004 | 0.884±0.003 | 0.898±0.001 | 0.855±0.003 | 0.889±0.001 | 0.9284 |
| TDP43 | **0.9237±0.002** | 0.8672 | 0.8619±0.004 | 0.875±0.003 | 0.865±0.004 | 0.845±0.001 | 0.824±0.004 | 0.8595 |
| TIA1 | **0.9275±0.003** | 0.8908 | 0.8653±0.002 | 0.846±0.002 | 0.846±0.004 | 0.856±0.001 | 0.84±0.003 | 0.8826 |
| TIAL1 | **0.8868±0.003** | 0.8526 | 0.8313±0.004 | 0.816±0.003 | 0.817±0.004 | 0.839±0.001 | 0.79±0.002 | 0.8626 |
| TNRC6 | **0.9312±0.001** | 0.8951 | 0.8998±0.001 | 0.72±0.001 | 0.67±0.003 | 0.661±0.003 | 0.601±0.004 | 0.8985 |
| U2AF65 | **0.9416±0.001** | 0.8734 | 0.8697±0.002 | 0.845±0.001 | 0.863±0.001 | 0.827±0.004 | 0.833±0.003 | 0.866 |
| WTAP | **0.9293±0.002** | 0.8766 | 0.8763±0.004 | 0.731±0.004 | 0.72±0.001 | 0.748±0.002 | 0.686±0.003 | 0.8768 |
| ZC3H7B | **0.7989±0.003** | 0.7819 | 0.7478±0.001 | 0.727±0.001 | 0.741±0.003 | 0.732±0.003 | 0.646±0.004 | 0.7574 |
| **AVG** | **0.8908±0.003** | 0.8635±0.044 | 0.8361±0.006 | 0.8114±0.007 | 0.8038±0.007 | 0.7751±0.006 | 0.7436±0.01 | 0.8464±0.057 |

**Supplementary Table 14.** Comparison of F1 of different methods on 37 circRNA datasets. Bold data represent the best F1 values of experimental results.
