# Supplementary material for: iCRBP-LKHA: Large convolutional kernel and hybrid channel-spatial attention for identifying circRNA-RBP interaction sites
Source: PLoS Comput Biol. 2024 Aug 22;20(8):e1012399. doi: 10.1371/journal.pcbi.1012399 (PMC11373821; doi:10.1371/journal.pcbi.1012399)
Supplement: S15 Table — Bold data represent the best MCC values of experimental results. (DOCX) [file pcbi.1012399.s015.docx]

| **Dataset37** | **iCRBP-LKHA** | **ASCRB** | **iCircRBP-DHN** | **PASSION** | **CRIP** | **CSCRites** | \| **CircSLNN** \| \| --- \| | **CRBPDL** |
| --- | --- | --- | --- | --- | --- | --- | --- | --- | --- |
| AGO1 | **0.7615±0.004** | 0.7194 | 0.7014±0.003 | 0.715±0.003 | 0.684±0.004 | 0.648±0.003 | 0.649±0.002 | 0.7137 |
| AGO2 | **0.7083±0.001** | 0.6705 | 0.6214±0.003 | 0.633±0.003 | 0.634±0.002 | 0.599±0.003 | 0.566±0.003 | 0.6357 |
| AGO3 | **0.789±0.004** | 0.7305 | 0.7178±0.002 | 0.683±0.001 | 0.691±0.001 | 0.649±0.004 | 0.648±0.002 | 0.7168 |
| ALKBH5 | **0.8043±0.002** | 0.7701 | 0.7717±0.004 | 0.565±0.003 | 0.564±0.001 | 0.613±0.001 | 0.45±0.001 | 0.7903 |
| AUF1 | **0.7971±0.002** | 0.789 | 0.7622±0.002 | 0.775±0.003 | 0.761±0.001 | 0.742±0.004 | 0.753±0.004 | 0.7609 |
| C17ORF85 | **0.8004±0.002** | 0.7636 | 0.7842±0.001 | 0.665±0.004 | 0.617±0.002 | 0.635±0.004 | 0.569±0.002 | 0.7664 |
| C22ORF28 | **0.7502±0.002** | 0.704 | 0.6865±0.001 | 0.684±0.002 | 0.69±0.001 | 0.682±0.002 | 0.612±0.002 | 0.723 |
| CAPRIN1 | **0.7486±0.001** | 0.7273 | 0.6577±0.001 | 0.687±0.001 | 0.636±0.003 | 0.641±0.004 | 0.586±0.003 | 0.6673 |
| DGCR8 | **0.7705±0.003** | 0.7178 | 0.6887±0.002 | 0.706±0.002 | 0.697±0.004 | 0.658±0.002 | 0.638±0.004 | 0.6966 |
| EIF4A3 | **0.6985±0.004** | 0.6804 | 0.605±0.003 | 0.65±0.003 | 0.624±0.003 | 0.634±0.002 | 0.569±0.004 | 0.6415 |
| EWSR1 | **0.7728±0.001** | 0.7604 | 0.7193±0.002 | 0.721±0.002 | 0.71±0.003 | 0.676±0.002 | 0.689±0.004 | 0.7494 |
| FMRP | **0.7607±0.004** | 0.7242 | 0.6909±0.004 | 0.715±0.002 | 0.683±0.004 | 0.707±0.002 | 0.654±0.004 | 0.6851 |
| FOX2 | **0.7891±0.002** | 0.7619 | 0.7338±0.003 | 0.644±0.001 | 0.641±0.002 | 0.598±0.002 | 0.47±0.004 | 0.7651 |
| FUS | **0.7082±0.003** | 0.7038 | 0.6442±0.003 | 0.657±0.003 | 0.66±0.004 | 0.635±0.001 | 0.615±0.001 | 0.6545 |
| FXR1 | **0.8046±0.003** | 0.7876 | 0.7542±0.003 | 0.761±0.004 | 0.757±0.003 | 0.666±0.003 | 0.731±0.004 | 0.7916 |
| FXR2 | **0.7842±0.002** | 0.763 | 0.7299±0.001 | 0.718±0.004 | 0.725±0.002 | 0.686±0.001 | 0.704±0.003 | 0.7587 |
| HNRNPC | **0.7938±0.002** | 0.7375 | 0.7773±0.003 | 0.737±0.004 | 0.762±0.001 | 0.755±0.003 | 0.746±0.003 | 0.7638 |
| HUR | **0.743±0.002** | 0.6915 | 0.6811±0.004 | 0.672±0.004 | 0.678±0.004 | 0.652±0.001 | 0.626±0.003 | 0.6982 |
| IGF2BP1 | **0.73±0.002** | 0.7156 | 0.6645±0.001 | 0.667±0.001 | 0.637±0.002 | 0.647±0.004 | 0.576±0.002 | 0.6649 |
| IGF2BP2 | 0.6905±0.002 | **0.6986** | 0.6543±0.004 | 0.625±0.004 | 0.619±0.004 | 0.592±0.002 | 0.566±0.002 | 0.6351 |
| IGF2BP3 | **0.7115±0.002** | 0.6995 | 0.622±0.002 | 0.662±0.002 | 0.65±0.003 | 0.588±0.001 | 0.553±0.004 | 0.6501 |
| LIN28A | **0.737±0.002** | 0.6785 | 0.6498±0.003 | 0.677±0.004 | 0.674±0.001 | 0.635±0.004 | 0.596±0.003 | 0.6775 |
| LIN28B | **0.7518±0.002** | 0.6488 | 0.6693±0.002 | 0.674±0.004 | 0.694±0.004 | 0.582±0.004 | 0.647±0.002 | 0.6811 |
| METTL3 | **0.7123±0.002** | 0.6947 | 0.6615±0.002 | 0.671±0.002 | 0.677±0.001 | 0.644±0.002 | 0.601±0.002 | 0.6881 |
| MOV10 | **0.7277±0.003** | 0.6786 | 0.6586±0.002 | 0.664±0.002 | 0.662±0.002 | 0.608±0.003 | 0.589±0.003 | 0.6599 |
| PTB | **0.7035±0.004** | 0.6838 | 0.6367±0.002 | 0.658±0.003 | 0.624±0.002 | 0.549±0.001 | 0.584±0.004 | 0.6607 |
| PUM2 | **0.7924±0.002** | 0.7855 | 0.7413±0.001 | 0.75±0.001 | 0.747±0.002 | 0.745±0.004 | 0.735±0.002 | 0.7602 |
| QKI | **0.8003±0.002** | 0.7691 | 0.7615±0.002 | 0.704±0.002 | 0.734±0.002 | 0.656±0.004 | 0.671±0.002 | 0.7898 |
| SFRS1 | **0.793±0.001** | 0.7407 | 0.7378±0.003 | 0.731±0.002 | 0.765±0.003 | 0.73±0.003 | 0.734±0.001 | 0.7278 |
| TAF15 | **0.8052±0.002** | 0.7785 | 0.7934±0.003 | 0.739±0.002 | 0.732±0.003 | 0.728±0.002 | 0.748±0.004 | 0.7845 |
| TDP43 | **0.7891±0.001** | 0.7511 | 0.7141±0.002 | 0.747±0.004 | 0.704±0.002 | 0.725±0.001 | 0.688±0.001 | 0.7119 |
| TIA1 | **0.7923±0.002** | 0.7284 | 0.7617±0.001 | 0.739±0.002 | 0.722±0.004 | 0.72±0.003 | 0.68±0.001 | 0.772 |
| TIAL1 | **0.7575±0.001** | 0.7312 | 0.6987±0.003 | 0.722±0.003 | 0.715±0.003 | 0.704±0.004 | 0.656±0.004 | 0.7337 |
| TNRC6 | **0.7954±0.001** | 0.7365 | 0.7585±0.003 | 0.619±0.001 | 0.57±0.001 | 0.572±0.003 | 0.525±0.001 | 0.7376 |
| U2AF65 | **0.8043±0.001** | 0.7391 | 0.7139±0.003 | 0.714±0.003 | 0.697±0.002 | 0.726±0.003 | 0.687±0.001 | 0.7077 |
| WTAP | **0.7938±0.002** | 0.7478 | 0.7675±0.002 | 0.616±0.003 | 0.606±0.001 | 0.63±0.004 | 0.56±0.001 | 0.7462 |
| ZC3H7B | 0.6824±0.001 | **0.6898** | 0.6069±0.004 | 0.615±0.001 | 0.627±0.004 | 0.596±0.003 | 0.55±0.001 | 0.6272 |
| **AVG** | **0.7609±0.003** | 0.7270 | 0.7027±0.006 | 0.686±0.007 | 0.678±0.007 | 0.655±0.006 | 0.6276±0.01 | 0.7134±0.057 |

**Supplementary Table 15.** Comparison of MCC of different methods on 37 circRNA datasets. Bold data represent the best MCC values of experimental results.
