# Supplementary material for: iCRBP-LKHA: Large convolutional kernel and hybrid channel-spatial attention for identifying circRNA-RBP interaction sites
Source: PLoS Comput Biol. 2024 Aug 22;20(8):e1012399. doi: 10.1371/journal.pcbi.1012399 (PMC11373821; doi:10.1371/journal.pcbi.1012399)
Supplement: S16 Table — (DOCX) [file pcbi.1012399.s016.docx]

|  | **Training Dataset** | **Testing Dataset** |
| --- | --- | --- |
| **Total number of samples** | **268849** | **67127** |
|  | AGO1 | PTB |
|  | AGO2 | QKI |
|  | AGO3 | SFRS1 |
|  | ALKBHS | TAF15 |
|  | AUF1 | TDP43 |
|  | C17ORF85 | TIA1 |
|  | C22ORF28 | TIAL1 |
|  | CAPRIN1 | TNRC6 |
|  | DGCR8 | U2AF65 |
|  | EIF4A3 | WTAP |
|  | EWSR1 | ZC3H7B |
|  | FMRP |  |
|  | FOX2 |  |
|  | FUS |  |
|  | FXR1 |  |
|  | FXR2 |  |
|  | HNRNPC |  |
|  | HUR |  |
|  | IGF2BP1 |  |
|  | IGF2BP2 |  |
|  | IGF2BP3 |  |
|  | LIN28A |  |
|  | LIN28B |  |
|  | METTL3 |  |
|  | MOV10 |  |
|  | PUM2 |  |

**Supplementary Table 16.** The circRNA names in training and testing datasets
