# Supplementary material for: iCRBP-LKHA: Large convolutional kernel and hybrid channel-spatial attention for identifying circRNA-RBP interaction sites
Source: PLoS Comput Biol. 2024 Aug 22;20(8):e1012399. doi: 10.1371/journal.pcbi.1012399 (PMC11373821; doi:10.1371/journal.pcbi.1012399)
Supplement: S17 Table — Bold data represent the best ACC values of experimental results. (DOCX) [file pcbi.1012399.s017.docx]

| **Dataset37** | **iCRBP-LKHA** | **ASCRB** | **iCircRBP-DHN** | **PASSION** | **CRIP** | **CSCRites** | \| **CircSLNN** \| \| --- \| | **CRBPDL** |
| --- | --- | --- | --- | --- | --- | --- | --- | --- | --- |
| AGO1 | **0.838±0.001** | 0.8024 | 0.6525±0.001 | 0.7±0.004 | 0.724±0.002 | 0.701±0.003 | 0.706±0.001 | 0.7395 |
| AGO2 | **0.763±0.002** | 0.6829 | 0.6181±0.003 | 0.623±0.001 | 0.624±0.003 | 0.58±0.004 | 0.564±0.004 | 0.6282 |
| AGO3 | **0.8495±0.001** | 0.7993 | 0.7785±0.004 | 0.758±0.002 | 0.697±0.002 | 0.662±0.003 | 0.644±0.003 | 0.7437 |
| ALKBH5 | **0.8887±0.004** | 0.7885 | 0.8187±0.004 | 0.626±0.003 | 0.564±0.003 | 0.65±0.003 | 0.477±0.002 | 0.8108 |
| AUF1 | **0.9254±0.002** | 0.8237 | 0.8504±0.003 | 0.808±0.003 | 0.82±0.003 | 0.775±0.003 | 0.843±0.003 | 0.8035 |
| C17ORF85 | **0.9105±0.002** | 0.8501 | 0.7927±0.002 | 0.704±0.004 | 0.705±0.002 | 0.714±0.003 | 0.572±0.004 | 0.8581 |
| C22ORF28 | **0.8493±0.001** | 0.7842 | 0.7646±0.003 | 0.713±0.001 | 0.767±0.004 | 0.712±0.003 | 0.675±0.003 | 0.7957 |
| CAPRIN1 | **0.7941±0.003** | 0.6798 | 0.7099±0.002 | 0.703±0.003 | 0.66±0.001 | 0.671±0.002 | 0.558±0.003 | 0.7153 |
| DGCR8 | **0.8571±0.003** | 0.7746 | 0.7178±0.003 | 0.76±0.004 | 0.738±0.003 | 0.675±0.004 | 0.669±0.003 | 0.7214 |
| EIF4A3 | **0.7559±0.001** | 0.7092 | 0.6362±0.003 | 0.64±0.004 | 0.649±0.004 | 0.638±0.002 | 0.543±0.002 | 0.6643 |
| EWSR1 | **0.8434±0.002** | 0.7994 | 0.7384±0.004 | 0.777±0.001 | 0.758±0.004 | 0.716±0.002 | 0.758±0.004 | 0.7851 |
| FMRP | **0.8362±0.001** | 0.76 | 0.7206±0.004 | 0.712±0.003 | 0.749±0.003 | 0.71±0.002 | 0.654±0.004 | 0.6886 |
| FOX2 | **0.8547±0.003** | 0.8005 | 0.7427±0.003 | 0.653±0.004 | 0.628±0.004 | 0.62±0.003 | 0.489±0.001 | 0.725 |
| FUS | **0.8123±0.004** | 0.783 | 0.6626±0.001 | 0.713±0.003 | 0.687±0.002 | 0.669±0.002 | 0.626±0.003 | 0.7052 |
| FXR1 | **0.9179±0.002** | 0.8591 | 0.8692±0.001 | 0.773±0.003 | 0.773±0.001 | 0.691±0.002 | 0.779±0.004 | 0.7961 |
| FXR2 | **0.862±0.003** | 0.7538 | 0.7787±0.002 | 0.766±0.001 | 0.721±0.002 | 0.667±0.004 | 0.709±0.002 | 0.8091 |
| HNRNPC | **0.9053±0.001** | 0.7898 | 0.8529±0.004 | 0.833±0.001 | 0.775±0.001 | 0.86±0.003 | 0.834±0.002 | 0.8111 |
| HUR | **0.8167±0.002** | 0.7277 | 0.6326±0.004 | 0.683±0.001 | 0.73±0.002 | 0.654±0.002 | 0.614±0.003 | 0.7108 |
| IGF2BP1 | **0.7893±0.003** | 0.7385 | 0.6714±0.004 | 0.671±0.001 | 0.637±0.002 | 0.645±0.003 | 0.588±0.002 | 0.6782 |
| IGF2BP2 | 0.7986±0.002 | **0.8286** | 0.6665±0.001 | 0.697±0.003 | 0.71±0.002 | 0.621±0.004 | 0.661±0.004 | 0.7176 |
| IGF2BP3 | **0.7738±0.002** | 0.7221 | 0.6552±0.003 | 0.663±0.002 | 0.675±0.004 | 0.628±0.002 | 0.578±0.002 | 0.6602 |
| LIN28A | **0.8148±0.003** | 0.7086 | 0.6468±0.001 | 0.701±0.002 | 0.71±0.002 | 0.682±0.002 | 0.608±0.002 | 0.7086 |
| LIN28B | **0.8195±0.001** | 0.7198 | 0.6827±0.002 | 0.707±0.002 | 0.682±0.004 | 0.612±0.003 | 0.64±0.002 | 0.7223 |
| METTL3 | 0.8023±0.001 | 0.7543 | **0.8531±0.001** | 0.737±0.004 | 0.686±0.001 | 0.695±0.003 | 0.655±0.002 | 0.7001 |
| MOV10 | **0.7682±0.001** | 0.6987 | 0.6792±0.001 | 0.629±0.004 | 0.641±0.004 | 0.578±0.002 | 0.579±0.004 | 0.6884 |
| PTB | **0.7689±0.003** | 0.6699 | 0.6227±0.001 | 0.631±0.002 | 0.631±0.002 | 0.551±0.002 | 0.594±0.001 | 0.6641 |
| PUM2 | **0.8918±0.002** | 0.7857 | 0.7979±0.004 | 0.796±0.002 | 0.79±0.003 | 0.785±0.001 | 0.774±0.001 | 0.793 |
| QKI | **0.8809±0.002** | 0.8056 | 0.8603±0.001 | 0.768±0.004 | 0.783±0.001 | 0.681±0.004 | 0.738±0.004 | 0.7806 |
| SFRS1 | **0.8834±0.002** | 0.8382 | 0.8052±0.002 | 0.827±0.003 | 0.798±0.003 | 0.796±0.003 | 0.716±0.003 | 0.8096 |
| TAF15 | **0.9314±0.004** | 0.8855 | 0.8967±0.002 | 0.865±0.002 | 0.84±0.002 | 0.838±0.001 | 0.825±0.004 | 0.8368 |
| TDP43 | **0.8428±0.001** | 0.7604 | 0.7858±0.003 | 0.756±0.004 | 0.734±0.001 | 0.696±0.001 | 0.721±0.003 | 0.7226 |
| TIA1 | **0.8817±0.003** | 0.7691 | 0.8608±0.002 | 0.78±0.003 | 0.752±0.001 | 0.767±0.004 | 0.746±0.004 | 0.7687 |
| TIAL1 | **0.8387±0.003** | 0.7967 | 0.7791±0.002 | 0.754±0.002 | 0.708±0.004 | 0.707±0.003 | 0.686±0.004 | 0.7295 |
| TNRC6 | **0.8841±0.001** | 0.7634 | 0.8526±0.002 | 0.626±0.002 | 0.587±0.003 | 0.625±0.004 | 0.527±0.001 | 0.7923 |
| U2AF65 | **0.8385±0.002** | 0.7076 | 0.6875±0.002 | 0.731±0.002 | 0.731±0.004 | 0.724±0.004 | 0.702±0.001 | 0.7237 |
| WTAP | **0.8898±0.001** | 0.8406 | 0.8504±0.001 | 0.656±0.002 | 0.679±0.003 | 0.676±0.004 | 0.601±0.003 | 0.7897 |
| ZC3H7B | **0.7514±0.003** | 0.6781 | 0.6603±0.004 | 0.674±0.003 | 0.656±0.001 | 0.626±0.003 | 0.534±0.002 | 0.6248 |
| **AVG** | **0.8413±0.003** | 0.7686±0.044 | 0.7473±0.006 | 0.719±0.007 | 0.708±0.007 | 0.6837±0.006 | 0.6537±0.01 | 0.7411±0.057 |

**Supplementary Table 17.** Comparison of ACC of different methods on 37 circRNAs stringent datasets. Bold data represent the best ACC values of experimental results.
