# Supplementary material for: iCRBP-LKHA: Large convolutional kernel and hybrid channel-spatial attention for identifying circRNA-RBP interaction sites
Source: PLoS Comput Biol. 2024 Aug 22;20(8):e1012399. doi: 10.1371/journal.pcbi.1012399 (PMC11373821; doi:10.1371/journal.pcbi.1012399)
Supplement: S18 Table — Bold data represent the best F1 values of experimental results. (DOCX) [file pcbi.1012399.s018.docx]

| **Dataset37** | **iCRBP-LKHA** | **ASCRB** | **iCircRBP-DHN** | **PASSION** | **CRIP** | **CSCRites** | \| **CircSLNN** \| \| --- \| | **CRBPDL** |
| --- | --- | --- | --- | --- | --- | --- | --- | --- | --- |
| AGO1 | **0.8413±0.004** | 0.7374 | 0.6558±0.003 | 0.746±0.003 | 0.703±0.003 | 0.706±0.003 | 0.716±0.003 | 0.7403 |
| AGO2 | **0.7746±0.004** | 0.6948 | 0.6297±0.002 | 0.63±0.003 | 0.665±0.004 | 0.618±0.001 | 0.566±0.001 | 0.679 |
| AGO3 | **0.8506±0.004** | 0.7634 | 0.7796±0.003 | 0.746±0.002 | 0.716±0.001 | 0.618±0.002 | 0.715±0.004 | 0.7786 |
| ALKBH5 | **0.9017±0.003** | 0.8115 | 0.8317±0.004 | 0.586±0.002 | 0.582±0.002 | 0.662±0.002 | 0.506±0.003 | 0.8237 |
| AUF1 | **0.9338±0.002** | 0.8363 | 0.8588±0.001 | 0.825±0.002 | 0.868±0.003 | 0.813±0.001 | 0.849±0.004 | 0.8445 |
| C17ORF85 | **0.9395±0.002** | 0.8464 | 0.8217±0.002 | 0.743±0.001 | 0.664±0.001 | 0.687±0.001 | 0.65±0.004 | 0.899 |
| C22ORF28 | **0.8732±0.003** | 0.8155 | 0.7885±0.004 | 0.759±0.003 | 0.774±0.002 | 0.713±0.003 | 0.698±0.003 | 0.8183 |
| CAPRIN1 | **0.8124±0.002** | 0.7031 | 0.7282±0.004 | 0.699±0.001 | 0.685±0.001 | 0.664±0.004 | 0.585±0.003 | 0.6616 |
| DGCR8 | **0.8626±0.002** | 0.7891 | 0.7233±0.001 | 0.787±0.004 | 0.731±0.003 | 0.746±0.002 | 0.697±0.002 | 0.7187 |
| EIF4A3 | **0.783±0.003** | 0.7101 | 0.6633±0.001 | 0.675±0.001 | 0.694±0.003 | 0.703±0.004 | 0.571±0.001 | 0.7213 |
| EWSR1 | **0.8092±0.001** | 0.7162 | 0.7042±0.002 | 0.728±0.001 | 0.705±0.004 | 0.659±0.002 | 0.704±0.002 | 0.6875 |
| FMRP | **0.8467±0.001** | 0.7498 | 0.7311±0.001 | 0.77±0.001 | 0.746±0.002 | 0.764±0.003 | 0.654±0.003 | 0.7718 |
| FOX2 | **0.8232±0.001** | 0.7717 | 0.7112±0.004 | 0.646±0.003 | 0.633±0.001 | 0.598±0.002 | 0.445±0.003 | 0.7606 |
| FUS | **0.8156±0.003** | 0.7635 | 0.6659±0.001 | 0.766±0.002 | 0.715±0.001 | 0.704±0.001 | 0.642±0.002 | 0.7128 |
| FXR1 | **0.9315±0.001** | 0.8303 | 0.8828±0.002 | 0.846±0.004 | 0.777±0.002 | 0.755±0.001 | 0.811±0.003 | 0.8924 |
| FXR2 | **0.8703±0.004** | 0.8088 | 0.787±0.001 | 0.793±0.002 | 0.733±0.003 | 0.686±0.001 | 0.726±0.001 | 0.8029 |
| HNRNPC | **0.9091±0.003** | 0.8185 | 0.8567±0.003 | 0.809±0.002 | 0.824±0.002 | 0.86±0.004 | 0.818±0.004 | 0.7921 |
| HUR | **0.7071±0.002** | 0.6323 | 0.523±0.002 | 0.606±0.003 | 0.603±0.003 | 0.603±0.003 | 0.542±0.001 | 0.629 |
| IGF2BP1 | **0.7992±0.004** | 0.7518 | 0.6813±0.003 | 0.666±0.003 | 0.676±0.001 | 0.666±0.004 | 0.618±0.004 | 0.679 |
| IGF2BP2 | **0.8381±0.003** | 0.8388 | 0.706±0.001 | 0.723±0.004 | 0.751±0.002 | 0.7±0.003 | 0.629±0.001 | 0.7634 |
| IGF2BP3 | **0.8115±0.001** | 0.7637 | 0.6929±0.001 | 0.682±0.003 | 0.674±0.002 | 0.6±0.002 | 0.613±0.001 | 0.7187 |
| LIN28A | **0.8412±0.004** | 0.7369 | 0.6732±0.004 | 0.726±0.002 | 0.688±0.004 | 0.706±0.002 | 0.679±0.001 | 0.7205 |
| LIN28B | **0.8285±0.004** | 0.7133 | 0.6917±0.004 | 0.746±0.001 | 0.705±0.003 | 0.596±0.002 | 0.683±0.001 | 0.7392 |
| METTL3 | 0.8009±0.003 | 0.7321 | **0.8517±0.003** | 0.742±0.003 | 0.68±0.003 | 0.673±0.001 | 0.619±0.002 | 0.7505 |
| MOV10 | **0.7574±0.003** | 0.654 | 0.6684±0.002 | 0.679±0.004 | 0.633±0.001 | 0.575±0.003 | 0.589±0.004 | 0.6456 |
| PTB | **0.7229±0.001** | 0.6786 | 0.5767±0.004 | 0.647±0.002 | 0.623±0.004 | 0.549±0.003 | 0.542±0.004 | 0.6116 |
| PUM2 | **0.894±0.001** | 0.7742 | 0.8001±0.001 | 0.795±0.001 | 0.778±0.003 | 0.784±0.001 | 0.742±0.002 | 0.7703 |
| QKI | **0.8722±0.004** | 0.7787 | 0.8516±0.003 | 0.735±0.003 | 0.718±0.002 | 0.705±0.002 | 0.722±0.003 | 0.7496 |
| SFRS1 | **0.863±0.003** | 0.8251 | 0.7848±0.004 | 0.813±0.001 | 0.737±0.001 | 0.77±0.003 | 0.708±0.003 | 0.7602 |
| TAF15 | **0.9309±0.004** | 0.8884 | 0.8962±0.002 | 0.824±0.001 | 0.834±0.002 | 0.839±0.001 | 0.78±0.004 | 0.8359 |
| TDP43 | **0.8388±0.001** | 0.7657 | 0.7818±0.003 | 0.746±0.002 | 0.721±0.004 | 0.684±0.003 | 0.668±0.002 | 0.7389 |
| TIA1 | **0.8728±0.003** | 0.7549 | 0.8519±0.003 | 0.762±0.002 | 0.77±0.003 | 0.76±0.003 | 0.739±0.001 | 0.8199 |
| TIAL1 | **0.8498±0.002** | 0.7528 | 0.7902±0.004 | 0.718±0.004 | 0.74±0.003 | 0.736±0.004 | 0.684±0.004 | 0.7678 |
| TNRC6 | **0.8902±0.003** | 0.8288 | 0.8587±0.001 | 0.663±0.003 | 0.588±0.002 | 0.625±0.003 | 0.537±0.002 | 0.7631 |
| U2AF65 | **0.802±0.002** | 0.7049 | 0.651±0.004 | 0.657±0.003 | 0.657±0.004 | 0.646±0.004 | 0.656±0.002 | 0.7003 |
| WTAP | **0.8649±0.003** | 0.8153 | 0.8255±0.003 | 0.661±0.003 | 0.667±0.004 | 0.658±0.004 | 0.61±0.001 | 0.7572 |
| ZC3H7B | **0.7641±0.001** | 0.681 | 0.673±0.003 | 0.654±0.002 | 0.666±0.004 | 0.65±0.002 | 0.578±0.004 | 0.6667 |
| **AVG** | **0.8401±0.003** | 0.7632±0.044 | 0.7473±0.006 | 0.724±0.007 | 0.7061±0.007 | 0.689±0.006 | 0.657±0.01 | 0.7484±0.057 |

**Supplementary Table 18.** Comparison of F1 of different methods on 37 circRNAs stringent datasets. Bold data represent the best F1 values of experimental results.
