# Supplementary material for: iCRBP-LKHA: Large convolutional kernel and hybrid channel-spatial attention for identifying circRNA-RBP interaction sites
Source: PLoS Comput Biol. 2024 Aug 22;20(8):e1012399. doi: 10.1371/journal.pcbi.1012399 (PMC11373821; doi:10.1371/journal.pcbi.1012399)
Supplement: S19 Table — Bold data represent the best MCC values of experimental results. (DOCX) [file pcbi.1012399.s019.docx]

| **Dataset37** | **iCRBP-LKHA** | **ASCRB** | **iCircRBP-DHN** | **PASSION** | **CRIP** | **CSCRites** | \| **CircSLNN** \| \| --- \| | **CRBPDL** |
| --- | --- | --- | --- | --- | --- | --- | --- | --- | --- |
| AGO1 | **0.7541±0.001** | 0.6894 | 0.6541±0.004 | 0.685±0.004 | 0.657±0.004 | 0.601±0.003 | 0.645±0.003 | 0.6649 |
| AGO2 | **0.7032±0.002** | 0.6536 | 0.6032±0.004 | 0.613±0.004 | 0.605±0.002 | 0.559±0.004 | 0.496±0.003 | 0.6207 |
| AGO3 | **0.8573±0.003** | 0.7837 | 0.7573±0.001 | 0.752±0.003 | 0.698±0.002 | 0.654±0.003 | 0.681±0.001 | 0.7266 |
| ALKBH5 | **0.9459±0.004** | 0.8796 | 0.8459±0.003 | 0.67±0.002 | 0.594±0.004 | 0.66±0.004 | 0.508±0.001 | 0.8178 |
| AUF1 | **0.9084±0.004** | 0.7843 | 0.8084±0.001 | 0.781±0.003 | 0.828±0.002 | 0.768±0.004 | 0.835±0.004 | 0.8269 |
| C17ORF85 | **0.8838±0.004** | 0.7738 | 0.7838±0.003 | 0.712±0.001 | 0.66±0.003 | 0.647±0.003 | 0.588±0.003 | 0.7808 |
| C22ORF28 | **0.8222±0.002** | 0.7535 | 0.7222±0.002 | 0.759±0.001 | 0.69±0.003 | 0.701±0.001 | 0.607±0.004 | 0.7533 |
| CAPRIN1 | **0.7595±0.004** | 0.686 | 0.6595±0.003 | 0.612±0.001 | 0.639±0.004 | 0.647±0.001 | 0.528±0.001 | 0.6861 |
| DGCR8 | **0.8407±0.001** | 0.7835 | 0.7407±0.003 | 0.697±0.002 | 0.764±0.001 | 0.676±0.004 | 0.642±0.004 | 0.7488 |
| EIF4A3 | **0.722±0.004** | 0.6458 | 0.622±0.003 | 0.631±0.003 | 0.596±0.004 | 0.616±0.004 | 0.525±0.002 | 0.6774 |
| EWSR1 | **0.8747±0.004** | 0.7717 | 0.7747±0.001 | 0.772±0.003 | 0.756±0.002 | 0.695±0.003 | 0.739±0.002 | 0.7641 |
| FMRP | **0.8094±0.003** | 0.7261 | 0.7094±0.003 | 0.725±0.004 | 0.682±0.001 | 0.707±0.003 | 0.616±0.004 | 0.6715 |
| FOX2 | **0.9351±0.003** | 0.8024 | 0.8351±0.004 | 0.758±0.001 | 0.731±0.001 | 0.629±0.003 | 0.514±0.003 | 0.8569 |
| FUS | **0.7699±0.004** | 0.7296 | 0.6699±0.003 | 0.723±0.003 | 0.682±0.001 | 0.621±0.001 | 0.64±0.001 | 0.7215 |
| FXR1 | **0.9543±0.004** | 0.8262 | 0.8543±0.002 | 0.797±0.003 | 0.795±0.003 | 0.74±0.001 | 0.83±0.002 | 0.8713 |
| FXR2 | **0.8407±0.002** | 0.7378 | 0.7407±0.004 | 0.707±0.004 | 0.725±0.001 | 0.666±0.001 | 0.707±0.001 | 0.7444 |
| HNRNPC | **0.9728±0.003** | 0.87 | 0.8728±0.004 | 0.923±0.001 | 0.915±0.001 | 0.854±0.004 | 0.921±0.004 | 0.8985 |
| HUR | **0.6321±0.003** | 0.5807 | 0.5321±0.003 | 0.57±0.004 | 0.575±0.002 | 0.525±0.004 | 0.492±0.004 | 0.5382 |
| IGF2BP1 | **0.7531±0.004** | 0.6837 | 0.6531±0.001 | 0.633±0.001 | 0.641±0.004 | 0.635±0.004 | 0.597±0.002 | 0.6301 |
| IGF2BP2 | **0.7407±0.002** | 0.699 | 0.6407±0.003 | 0.628±0.002 | 0.662±0.004 | 0.568±0.004 | 0.561±0.003 | 0.699 |
| IGF2BP3 | **0.7268±0.003** | 0.6913 | 0.6268±0.003 | 0.652±0.002 | 0.639±0.001 | 0.58±0.004 | 0.53±0.004 | 0.6484 |
| LIN28A | **0.7292±0.001** | 0.6837 | 0.6292±0.001 | 0.615±0.002 | 0.606±0.004 | 0.641±0.003 | 0.554±0.002 | 0.6514 |
| LIN28B | **0.7706±0.003** | 0.6176 | 0.6706±0.004 | 0.692±0.001 | 0.675±0.002 | 0.574±0.003 | 0.603±0.003 | 0.6763 |
| METTL3 | 0.7652±0.002 | 0.7407 | **0.8652±0.001** | 0.674±0.002 | 0.705±0.003 | 0.668±0.001 | 0.587±0.004 | 0.7139 |
| MOV10 | **0.7616±0.002** | 0.7022 | 0.6616±0.002 | 0.618±0.001 | 0.661±0.003 | 0.58±0.004 | 0.569±0.003 | 0.6423 |
| PTB | **0.7644±0.003** | 0.6929 | 0.6644±0.004 | 0.658±0.003 | 0.685±0.001 | 0.578±0.004 | 0.582±0.001 | 0.6703 |
| PUM2 | **0.8964±0.003** | 0.8275 | 0.7964±0.004 | 0.759±0.004 | 0.831±0.001 | 0.762±0.002 | 0.774±0.002 | 0.7692 |
| QKI | **0.9469±0.003** | 0.9016 | 0.8469±0.003 | 0.775±0.003 | 0.797±0.002 | 0.775±0.004 | 0.742±0.003 | 0.8391 |
| SFRS1 | **0.9266±0.004** | 0.8605 | 0.8266±0.001 | 0.813±0.001 | 0.83±0.003 | 0.855±0.003 | 0.761±0.001 | 0.7937 |
| TAF15 | **0.974±0.001** | 0.8651 | 0.874±0.004 | 0.838±0.001 | 0.834±0.001 | 0.837±0.002 | 0.851±0.002 | 0.8483 |
| TDP43 | **0.8825±0.002** | 0.8175 | 0.7825±0.002 | 0.744±0.003 | 0.804±0.004 | 0.726±0.001 | 0.697±0.004 | 0.7974 |
| TIA1 | 0.8864±0.001 | 0.7695 | **0.8865±0.002** | 0.751±0.002 | 0.782±0.001 | 0.736±0.004 | 0.754±0.002 | 0.8058 |
| TIAL1 | **0.9066±0.004** | 0.8242 | 0.8066±0.003 | 0.758±0.002 | 0.768±0.003 | 0.804±0.003 | 0.763±0.002 | 0.8059 |
| TNRC6 | **0.9467±0.003** | 0.8653 | 0.8467±0.004 | 0.654±0.001 | 0.683±0.003 | 0.648±0.004 | 0.577±0.001 | 0.8247 |
| U2AF65 | **0.8402±0.001** | 0.7268 | 0.7402±0.003 | 0.686±0.001 | 0.689±0.001 | 0.702±0.002 | 0.703±0.003 | 0.7228 |
| WTAP | **0.8808±0.002** | 0.7794 | 0.8808±0.002 | 0.664±0.004 | 0.673±0.004 | 0.636±0.001 | 0.611±0.003 | 0.7608 |
| ZC3H7B | **0.7548±0.004** | 0.7054 | 0.6548±0.002 | 0.637±0.004 | 0.647±0.004 | 0.633±0.002 | 0.568±0.002 | 0.6578 |
| **AVG** | **0.8335±0.003** | 0.7549±0.044 | 0.7443±0.006 | 0.706±0.007 | 0.708±0.007 | 0.673±0.006 | 0.646±0.01 | 0.7386±0.057 |

**Supplementary Table 19.** Comparison of MCC of different methods on 37 circRNAs stringent datasets. Bold data represent the best MCC values of experimental results.
