# Supplementary material for: iCRBP-LKHA: Large convolutional kernel and hybrid channel-spatial attention for identifying circRNA-RBP interaction sites
Source: PLoS Comput Biol. 2024 Aug 22;20(8):e1012399. doi: 10.1371/journal.pcbi.1012399 (PMC11373821; doi:10.1371/journal.pcbi.1012399)
Supplement: S20 Table — Bold data represent the best ACC values of experimental results. (DOCX) [file pcbi.1012399.s020.docx]

**Supplementary Table 20.** Comparison of ACC of different methods on 31 linear RNAs datasets. Bold data represent the best ACC values of experimental results.

| **Dataset31** | **iCRBP-LKHA** | **ASCRB** | **iCircRBP-DHN** | **CRIP** | **CRBPDL** | **iDeepS** | **CSCRites** | **CircSLNN** |
| --- | --- | --- | --- | --- | --- | --- | --- | --- |
| AGO1234 | 0.8119±0.002 | **0.8788** | 0.7583±0.002 | 0.718±0.004 | 0.789 | 0.673 | 0.68±0.001 | 0.6235±0.001 |
| AGO2MNAS | **0.8417±0.003** | 0.7982 | 0.6989±0.001 | 0.564±0.004 | 0.771 | 0.533 | 0.557±0.002 | 0.5234±0.002 |
| 2-bingding_1 | **0.969±0.003** | 0.9081 | 0.9051±0.003 | 0.839±0.004 | 0.886 | 0.742 | 0.802±0.004 | 0.7402±0.001 |
| 2-bingding_2 | **0.9666±0.001** | 0.9426 | 0.9045±0.004 | 0.819±0.002 | 0.878 | 0.798 | 0.805±0.004 | 0.7236±0.003 |
| AGO2 | 0.8307±0.003 | 0.7815 | 0.781±0.004 | 0.616±0.004 | **0.85** | 0.624 | 0.597±0.001 | 0.5284±0.001 |
| eIF4AIII_1 | **0.9719±0.002** | 0.915 | 0.9044±0.004 | 0.909±0.001 | 0.946 | 0.893 | 0.897±0.001 | 0.8636±0.002 |
| eIF4AIII_2 | **0.9853±0.004** | 0.9222 | 0.9332±0.002 | 0.91±0.004 | 0.952 | 0.902 | 0.882±0.001 | 0.8472±0.002 |
| ELVAL1-1 | 0.8719±0.002 | 0.9027 | **0.9171±0.001** | 0.895±0.002 | 0.888 | 0.886 | 0.87±0.002 | 0.8237±0.002 |
| ELVAL1-MNASE | **0.8425±0.002** | 0.8247 | 0.6609±0.004 | 0.572±0.002 | 0.715 | 0.554 | 0.546±0.002 | 0.4868±0.002 |
| ELVAL1-A | **0.9405±0.001** | 0.9247 | 0.9006±0.004 | 0.874±0.001 | 0.901 | 0.862 | 0.817±0.002 | 0.8235±0.001 |
| ELVAL1-2 | **0.9572±0.002** | 0.9043 | 0.8937±0.003 | 0.864±0.004 | 0.883 | 0.896 | 0.903±0.003 | 0.8664±0.003 |
| EWSR1 | **0.944±0.004** | 0.8806 | 0.8608±0.002 | 0.891±0.003 | 0.883 | 0.89 | 0.83±0.004 | 0.8244±0.001 |
| FUS | **0.9533±0.002** | 0.9039 | 0.9245±0.002 | 0.906±0.002 | 0.908 | 0.893 | 0.849±0.001 | 0.8762±0.004 |
| mut-FUS | **0.9558±0.004** | 0.9245 | 0.9249±0.002 | 0.902±0.003 | 0.898 | 0.885 | 0.854±0.004 | 0.8818±0.001 |
| IGF2BP1-3 | **0.9023±0.003** | 0.8573 | 0.7333±0.002 | 0.648±0.001 | 0.767 | 0.645 | 0.687±0.004 | 0.573±0.001 |
| hnRNPC-1 | **0.9541±0.003** | 0.9473 | 0.8873±0.003 | 0.915±0.003 | 0.886 | 0.939 | 0.905±0.001 | 0.8841±0.004 |
| hnRNPC-2 | **0.975±0.002** | 0.961 | 0.9396±0.004 | 0.955±0.004 | 0.93 | 0.924 | 0.931±0.004 | 0.8958±0.002 |
| hnRNPL-1 | **0.8771±0.003** | 0.8247 | 0.7823±0.002 | 0.721±0.003 | 0.839 | 0.632 | 0.61±0.003 | 0.6518±0.001 |
| hnRNPL-2 | **0.8757±0.004** | 0.8046 | 0.738±0.003 | 0.689±0.004 | 0.748 | 0.649 | 0.596±0.002 | 0.6127±0.002 |
| HnRNPL-like | **0.8673±0.001** | 0.8295 | 0.7496±0.004 | 0.667±0.003 | 0.775 | 0.601 | 0.59±0.003 | 0.6172±0.001 |
| MOV10 | 0.8807±0.002 | **0.8867** | 0.84±0.001 | 0.764±0.004 | 0.854 | 0.765 | 0.771±0.003 | 0.7143±0.001 |
| NSUN2 | 0.8732±0.004 | **0.8994** | 0.7985±0.001 | 0.825±0.002 | 0.841 | 0.76 | 0.747±0.003 | 0.7405±0.002 |
| PUM2 | **0.9759±0.001** | 0.9384 | 0.9019±0.004 | 0.904±0.001 | 0.926 | 0.91 | 0.927±0.003 | 0.8813±0.002 |
| QKI | **0.9661±0.004** | 0.9 | 0.8961±0.002 | 0.924±0.002 | 0.945 | 0.943 | 0.903±0.003 | 0.8955±0.004 |
| SFRS1 | **0.9403±0.001** | 0.9001 | 0.8912±0.001 | 0.861±0.002 | 0.889 | 0.853 | 0.836±0.004 | 0.7561±0.002 |
| TAF1S | **0.9729±0.004** | 0.9209 | 0.9038±0.003 | 0.902±0.002 | 0.907 | 0.93 | 0.875±0.003 | 0.862±0.004 |
| TDP-43 | **0.9296±0.004** | 0.8948 | 0.8903±0.001 | 0.861±0.003 | 0.894 | 0.868 | 0.859±0.003 | 0.8215±0.004 |
| TIA1 | **0.9595±0.001** | 0.9036 | 0.8889±0.003 | 0.888±0.002 | 0.939 | 0.86 | 0.869±0.002 | 0.8522±0.004 |
| TIAL1 | **0.9705±0.003** | 0.8832 | 0.8848±0.004 | 0.877±0.002 | 0.936 | 0.854 | 0.822±0.004 | 0.7938±0.003 |
| U2AF65 | **0.9764±0.004** | 0.9631 | 0.9048±0.004 | 0.901±0.002 | 0.919 | 0.938 | 0.857±0.003 | 0.8977±0.004 |
| Y2AF65 | **0.9599±0.004** | 0.9502 | 0.8893±0.004 | 0.885±0.002 | 0.94 | 0.875 | 0.86±0.004 | 0.8637±0.002 |
| **Avg** | 0.9258±0.003 | **0.8928±0.047** | 0.8544±0.08 | 0.821±0.12 | 0.874±0.068 | 0.806±0.13 | 0.791±0.12 | 0.7660±0.13 |
