# Supplementary material for: iCRBP-LKHA: Large convolutional kernel and hybrid channel-spatial attention for identifying circRNA-RBP interaction sites
Source: PLoS Comput Biol. 2024 Aug 22;20(8):e1012399. doi: 10.1371/journal.pcbi.1012399 (PMC11373821; doi:10.1371/journal.pcbi.1012399)
Supplement: S21 Table — Bold data represent the best F1 values of experimental results. (DOCX) [file pcbi.1012399.s021.docx]

**Supplementary Table 21.** Comparison of F1 of different methods on 31 linear RNAs datasets. Bold data represent the best F1 values of experimental results.

| **Dataset31** | **iCRBP-LKHA** | **ASCRB** | **iCircRBP-DHN** | **CRIP** | **CRBPDL** | **iDeepS** | **CSCRites** | **CircSLNN** |
| --- | --- | --- | --- | --- | --- | --- | --- | --- |
| AGO1234 | 0.7746±0.004 | **0.8373** | 0.7076±0.003 | 0.659±0.004 | 0.744 | 0.643 | 0.649±0.001 | 0.5849±0.002 |
| AGO2MNAS | **0.803±0.004** | 0.7697 | 0.6775±0.003 | 0.532±0.004 | 0.713 | 0.498 | 0.514±0.004 | 0.4973±0.002 |
| 2-bingding_1 | **0.9244±0.002** | 0.9022 | 0.827±0.004 | 0.792±0.004 | 0.832 | 0.724 | 0.759±0.001 | 0.7139±0.001 |
| 2-bingding_2 | **0.9221±0.003** | 0.8798 | 0.8629±0.003 | 0.777±0.002 | 0.839 | 0.737 | 0.742±0.003 | 0.6978±0.001 |
| AGO2 | 0.7925±0.003 | 0.7625 | 0.7196±0.004 | 0.565±0.004 | **0.802** | 0.583 | 0.591±0.003 | 0.5201±0.004 |
| eIF4AIII_1 | **0.9272±0.004** | 0.8821 | 0.8528±0.001 | 0.843±0.003 | 0.863 | 0.869 | 0.86±0.001 | 0.8138±0.004 |
| eIF4AIII_2 | **0.94±0.002** | 0.8943 | 0.8801±0.004 | 0.873±0.002 | 0.883 | 0.872 | 0.869±0.004 | 0.8144±0.004 |
| ELVAL1-1 | 0.8318±0.004 | 0.8476 | **0.8715±0.003** | 0.824±0.004 | 0.844 | 0.822 | 0.801±0.004 | 0.7876±0.001 |
| ELVAL1-MNASE | **0.8038±0.001** | 0.7613 | 0.6126±0.003 | 0.538±0.002 | 0.661 | 0.515 | 0.526±0.002 | 0.4775±0.002 |
| ELVAL1-A | **0.8973±0.001** | 0.8742 | 0.8563±0.002 | 0.821±0.004 | 0.852 | 0.786 | 0.776±0.004 | 0.7855±0.001 |
| ELVAL1-2 | **0.9132±0.004** | 0.8836 | 0.8682±0.003 | 0.858±0.004 | 0.841 | 0.865 | 0.823±0.001 | 0.8341±0.003 |
| EWSR1 | **0.9006±0.001** | 0.8415 | 0.8436±0.001 | 0.817±0.003 | 0.83 | 0.812 | 0.802±0.002 | 0.7548±0.004 |
| FUS | **0.9094±0.002** | 0.8624 | 0.8591±0.002 | 0.854±0.003 | 0.879 | 0.848 | 0.829±0.003 | 0.8082±0.004 |
| mut-FUS | **0.9119±0.001** | 0.8778 | 0.8789±0.001 | 0.872±0.003 | 0.849 | 0.832 | 0.812±0.002 | 0.8416±0.001 |
| IGF2BP1-3 | **0.8608±0.001** | 0.8017 | 0.7213±0.003 | 0.632±0.003 | 0.718 | 0.638 | 0.638±0.002 | 0.533±0.002 |
| hnRNPC-1 | **0.9103±0.002** | 0.8878 | 0.8393±0.002 | 0.859±0.003 | 0.853 | 0.872 | 0.847±0.001 | 0.8462±0.002 |
| hnRNPC-2 | **0.9302±0.003** | 0.902 | 0.8591±0.004 | 0.898±0.003 | 0.917 | 0.893 | 0.869±0.001 | 0.8623±0.002 |
| hnRNPL-1 | **0.8368±0.001** | 0.7786 | 0.7471±0.002 | 0.694±0.002 | 0.768 | 0.602 | 0.602±0.001 | 0.6133±0.002 |
| hnRNPL-2 | **0.8355±0.004** | 0.7707 | 0.6779±0.004 | 0.668±0.004 | 0.733 | 0.616 | 0.563±0.002 | 0.5839±0.004 |
| HnRNPL-like | **0.8275±0.003** | 0.7635 | 0.7096±0.001 | 0.634±0.004 | 0.723 | 0.593 | 0.57±0.004 | 0.5906±0.001 |
| MOV10 | 0.8402±0.002 | **0.864** | 0.7954±0.004 | 0.723±0.004 | 0.794 | 0.748 | 0.718±0.001 | 0.691±0.003 |
| NSUN2 | 0.833±0.002 | **0.8682** | 0.7529±0.004 | 0.791±0.001 | 0.806 | 0.722 | 0.724±0.004 | 0.6938±0.001 |
| PUM2 | **0.931±0.001** | 0.876 | 0.8949±0.003 | 0.862±0.002 | 0.91 | 0.883 | 0.868±0.002 | 0.8487±0.004 |
| QKI | **0.9217±0.001** | 0.8711 | 0.8536±0.003 | 0.881±0.004 | 0.901 | 0.89 | 0.875±0.002 | 0.8259±0.004 |
| SFRS1 | **0.8971±0.002** | 0.8457 | 0.8202±0.003 | 0.782±0.003 | 0.824 | 0.808 | 0.803±0.001 | 0.7152±0.002 |
| TAF1S | **0.9282±0.004** | 0.8852 | 0.8899±0.002 | 0.851±0.002 | 0.84 | 0.851 | 0.848±0.003 | 0.8485±0.004 |
| TDP-43 | **0.8868±0.003** | 0.8479 | 0.8592±0.004 | 0.822±0.003 | 0.838 | 0.81 | 0.811±0.003 | 0.7629±0.002 |
| TIA1 | **0.9154±0.004** | 0.8676 | 0.8438±0.003 | 0.823±0.003 | 0.893 | 0.816 | 0.79±0.003 | 0.8212±0.004 |
| TIAL1 | **0.9259±0.004** | 0.8443 | 0.8266±0.002 | 0.818±0.004 | 0.9 | 0.794 | 0.798±0.003 | 0.7677±0.003 |
| U2AF65 | **0.9315±0.002** | 0.8775 | 0.8855±0.002 | 0.893±0.003 | 0.909 | 0.889 | 0.813±0.003 | 0.8384±0.003 |
| Y2AF65 | **0.9157±0.004** | 0.8666 | 0.8599±0.002 | 0.829±0.001 | 0.873 | 0.833 | 0.821±0.002 | 0.8252±0.004 |
| **Avg** | 0.8832±0.003 | **0.8482±0.047** | 0.8114±0.08 | 0.777±0.12 | 0.827±0.068 | 0.763±0.13 | 0.752±0.12 | 0.7290±0.13 |
