# Supplementary material for: iCRBP-LKHA: Large convolutional kernel and hybrid channel-spatial attention for identifying circRNA-RBP interaction sites
Source: PLoS Comput Biol. 2024 Aug 22;20(8):e1012399. doi: 10.1371/journal.pcbi.1012399 (PMC11373821; doi:10.1371/journal.pcbi.1012399)
Supplement: S22 Table — Bold data represent the best MCC values of experimental results. (DOCX) [file pcbi.1012399.s022.docx]

**Supplementary Table 22.** Comparison of MCC of different methods on 31 linear RNAs datasets. Bold data represent the best MCC values of experimental results.

| **Dataset31** | **iCRBP-LKHA** | **ASCRB** | **iCircRBP-DHN** | **CRIP** | **CRBPDL** | **iDeepS** | **CSCRites** | **CircSLNN** |
| --- | --- | --- | --- | --- | --- | --- | --- | --- |
| AGO1234 | 0.6626±0.001 | **0.7303** | 0.5931±0.002 | 0.588±0.002 | 0.636 | 0.552 | 0.564±0.002 | 0.5077±0.002 |
| AGO2MNAS | **0.6869±0.003** | 0.6423 | 0.5708±0.003 | 0.474±0.004 | 0.607 | 0.444 | 0.455±0.001 | 0.4332±0.001 |
| 2-bingding_1 | **0.7908±0.003** | 0.7506 | 0.7059±0.001 | 0.68±0.002 | 0.734 | 0.626 | 0.672±0.002 | 0.6306±0.002 |
| 2-bingding_2 | **0.7888±0.001** | 0.7788 | 0.6969±0.004 | 0.676±0.004 | 0.719 | 0.637 | 0.631±0.004 | 0.5916±0.004 |
| AGO2 | 0.6779±0.004 | 0.6305 | 0.6036±0.003 | 0.487±0.004 | **0.699** | 0.479 | 0.498±0.001 | 0.438±0.002 |
| eIF4AIII_1 | **0.7932±0.002** | 0.7763 | 0.7625±0.002 | 0.736±0.003 | 0.761 | 0.719 | 0.72±0.001 | 0.6864±0.004 |
| eIF4AIII_2 | **0.8041±0.004** | 0.7746 | 0.7486±0.004 | 0.74±0.003 | 0.764 | 0.72 | 0.753±0.001 | 0.7155±0.001 |
| ELVAL1-1 | 0.7115±0.003 | 0.7236 | 0.7089±0.001 | 0.69±0.003 | **0.727** | 0.702 | 0.72±0.004 | 0.6803±0.003 |
| ELVAL1-MNASE | **0.6876±0.004** | 0.642 | 0.5518±0.004 | 0.46±0.001 | 0.556 | 0.441 | 0.442±0.001 | 0.4105±0.004 |
| ELVAL1-A | **0.7675±0.003** | 0.7364 | 0.7235±0.003 | 0.709±0.004 | 0.74 | 0.67 | 0.662±0.004 | 0.6703±0.004 |
| ELVAL1-2 | **0.7812±0.003** | 0.7451 | 0.7245±0.003 | 0.707±0.004 | 0.737 | 0.703 | 0.736±0.004 | 0.6794±0.001 |
| EWSR1 | **0.7704±0.004** | 0.7235 | 0.7158±0.002 | 0.7±0.002 | 0.698 | 0.722 | 0.697±0.003 | 0.6477±0.002 |
| FUS | **0.7779±0.002** | 0.7401 | 0.7293±0.003 | 0.722±0.003 | 0.734 | 0.722 | 0.709±0.001 | 0.6826±0.002 |
| mut-FUS | **0.78±0.002** | 0.7246 | 0.7442±0.003 | 0.715±0.002 | 0.725 | 0.73 | 0.699±0.001 | 0.7184±0.004 |
| IGF2BP1-3 | **0.7364±0.001** | 0.6898 | 0.6143±0.002 | 0.523±0.002 | 0.635 | 0.53 | 0.54±0.003 | 0.4637±0.001 |
| hnRNPC-1 | **0.7787±0.003** | 0.7656 | 0.7244±0.001 | 0.735±0.002 | 0.748 | 0.759 | 0.72±0.001 | 0.715±0.001 |
| hnRNPC-2 | **0.7957±0.001** | 0.7574 | 0.7589±0.001 | 0.776±0.004 | 0.779 | 0.739 | 0.74±0.001 | 0.7412±0.003 |
| hnRNPL-1 | **0.7158±0.001** | 0.706 | 0.6579±0.002 | 0.591±0.003 | 0.676 | 0.524 | 0.504±0.003 | 0.5204±0.003 |
| hnRNPL-2 | **0.7147±0.003** | 0.6585 | 0.5944±0.001 | 0.566±0.003 | 0.604 | 0.526 | 0.482±0.004 | 0.5191±0.003 |
| HnRNPL-like | **0.7078±0.004** | 0.6517 | 0.6041±0.001 | 0.544±0.003 | 0.637 | 0.484 | 0.491±0.002 | 0.5063±0.003 |
| MOV10 | 0.7187±0.001 | **0.7417** | 0.7068±0.003 | 0.649±0.001 | 0.678 | 0.61 | 0.625±0.003 | 0.5785±0.003 |
| NSUN2 | 0.7126±0.002 | **0.7306** | 0.649±0.003 | 0.673±0.004 | 0.672 | 0.607 | 0.614±0.003 | 0.6156±0.002 |
| PUM2 | **0.7964±0.001** | 0.7749 | 0.7694±0.001 | 0.767±0.004 | 0.747 | 0.755 | 0.746±0.004 | 0.6963±0.002 |
| QKI | **0.7884±0.004** | 0.7727 | 0.7352±0.002 | 0.733±0.001 | 0.735 | 0.754 | 0.759±0.002 | 0.7028±0.004 |
| SFRS1 | **0.7674±0.002** | 0.7287 | 0.6923±0.002 | 0.674±0.004 | 0.725 | 0.671 | 0.671±0.001 | 0.6183±0.004 |
| TAF1S | **0.794±0.001** | 0.7385 | 0.7351±0.002 | 0.739±0.003 | 0.743 | 0.768 | 0.734±0.003 | 0.7364±0.001 |
| TDP-43 | **0.7586±0.003** | 0.7497 | 0.7122±0.004 | 0.693±0.002 | 0.741 | 0.689 | 0.723±0.001 | 0.6363±0.004 |
| TIA1 | **0.783±0.003** | 0.7587 | 0.7228±0.004 | 0.725±0.004 | 0.765 | 0.713 | 0.706±0.003 | 0.6795±0.003 |
| TIAL1 | **0.7921±0.001** | 0.7466 | 0.7271±0.003 | 0.718±0.004 | 0.747 | 0.673 | 0.675±0.003 | 0.6404±0.001 |
| U2AF65 | **0.7968±0.003** | 0.7581 | 0.7304±0.004 | 0.737±0.004 | 0.739 | 0.752 | 0.726±0.002 | 0.738±0.004 |
| Y2AF65 | **0.7833±0.004** | 0.7552 | 0.7135±0.003 | 0.746±0.001 | 0.773 | 0.718 | 0.719±0.003 | 0.6839±0.004 |
| **Avg** | 0.7555±0.003 | **0.7291±0.047** | 0.6912±0.08 | 0.667±0.12 | 0.709±0.068 | 0.650±0.13 | 0.649±0.12 | 0.6221±0.13 |
